# A multilayer multimodal detection and prediction model based on explainable artificial intelligence for Alzheimer’s disease

**DOI:** 10.1038/s41598-021-82098-3

**Published:** 2021-01-29

**Authors:** Shaker El-Sappagh, Jose M. Alonso, S. M. Riazul Islam, Ahmad M. Sultan, Kyung Sup Kwak

**Affiliations:** 1 Centro Singular de Investigación en Tecnoloxías Intelixentes (CiTIUS), Universidade de Santiago de Compostela, 15782 Santiago de Compostela, Spain; 2 Information Systems Department, Faculty of Computers and Artificial Intelligence, Benha University, Banha, 13518 Egypt; 3 Centro Singular de Investigación en Tecnoloxías Intelixentes, Universidade de Santiago de Compostela, 15703 Santiago, Spain; 4 Department of Computer Science and Engineering, Sejong University, 209 Neungdong-ro, Gwangjin-gu, Seoul, 05006 Korea; 5 Gastrointestinal Surgical Center, Faculty of Medicine, Mansoura University, Mansura, 35516 Egypt; 6 Department of Information and Communication Engineering, Inha University, Incheon, 22212 South Korea

**Keywords:** Classification and taxonomy, Computational neuroscience, Data mining, Data processing, Machine learning

## Abstract

Alzheimer’s disease (AD) is the most common type of dementia. Its diagnosis and progression detection have been intensively studied. Nevertheless, research studies often have little effect on clinical practice mainly due to the following reasons: (1) Most studies depend mainly on a single modality, especially neuroimaging; (2) diagnosis and progression detection are usually studied separately as two independent problems; and (3) current studies concentrate mainly on optimizing the performance of complex machine learning models, while disregarding their explainability. As a result, physicians struggle to interpret these models, and feel it is hard to trust them. In this paper, we carefully develop an accurate and interpretable AD diagnosis and progression detection model. This model provides physicians with accurate decisions along with a set of explanations for every decision. Specifically, the model integrates 11 modalities of 1048 subjects from the Alzheimer’s Disease Neuroimaging Initiative (ADNI) real-world dataset: 294 cognitively normal, 254 stable mild cognitive impairment (MCI), 232 progressive MCI, and 268 AD. It is actually a two-layer model with random forest (RF) as classifier algorithm. In the first layer, the model carries out a multi-class classification for the early diagnosis of AD patients. In the second layer, the model applies binary classification to detect possible MCI-to-AD progression within three years from a baseline diagnosis. The performance of the model is optimized with key markers selected from a large set of biological and clinical measures. Regarding explainability, we provide, for each layer, global and instance-based explanations of the RF classifier by using the SHapley Additive exPlanations (SHAP) feature attribution framework. In addition, we implement 22 explainers based on decision trees and fuzzy rule-based systems to provide complementary justifications for every RF decision in each layer. Furthermore, these explanations are represented in natural language form to help physicians understand the predictions. The designed model achieves a cross-validation accuracy of 93.95% and an F1-score of 93.94% in the first layer, while it achieves a cross-validation accuracy of 87.08% and an F1-Score of 87.09% in the second layer. The resulting system is not only accurate, but also trustworthy, accountable, and medically applicable, thanks to the provided explanations which are broadly consistent with each other and with the AD medical literature. The proposed system can help to enhance the clinical understanding of AD diagnosis and progression processes by providing detailed insights into the effect of different modalities on the disease risk.

## Introduction

Alzheimer’s disease (AD) is a chronic neurodegenerative disease. This irreversible disorder is characterized by abnormal accumulation of amyloid plaques and neurofibrillary tangles in the brain, resulting in progressive decline in memory, thinking and language skills, along with behavioral changes. With increased human life expectancy, 11 million to 16 million elderly people are likely to suffer from AD by 2050^1^. As far as we know, there is no effective recovery for this disease. However, early detection is of fundamental importance for timely treatment and progression delay^2–4^. Furthermore, prediction of the probable progression of the disease from mild cognitive impairment (MCI) to AD is of critical importance^5,6^. MCI is considered an intermediate stage between age-associated cognitive impairment and AD. For effective treatment, it is therefore essential to detect patients with MCI at high risk of progression to AD^7^. As a result, AD diagnosis and progression detection are multistage in nature. First, physicians determine the category of the patient (MCI or AD). Second, they deeply investigate patient biomarkers to determine progression status to AD from MCI. Most studies in the literature focus either on AD diagnosis^1,8–12^ or MCI progression, i.e., progressive MCI (pMCI) versus stable MCI (sMCI)^13–15^. Even if it is highly desirable to deal simultaneously with AD diagnosis and MCI progression, this task is extremely hard mainly due to the multimodality nature that also jeopardizes explainability.

AD symptomatology is multimodal in nature^4,16^ correlated with cognitive scores, neuropathology vital signs, symptoms, demographics, medical history, neuropsychological battery, lab tests, etc. Complementary information exists among the modalities, which can be exploited to build powerful classifiers^17^. Therefore, medically intuitive AD detection methods should not rely only on measurements of a unique domain, such as physiological or behavioral symptoms. Alberdi et al.^1^ surveyed the AD diagnosis studies based on multimodal data. The combination of multimodalities facilitates the detection of subtle changes in all modalities from the very beginning, which results in reliable diagnoses. Once in the hands of an expert, it is still a challenge to correctly diagnose AD. Usually, medical experts are not able to manually analyze all of these vast and diverse biomarkers, and recognize the so-small behavioral shifts in AD patients until it is too late^18^. AD could be diagnosed after two years of memory problems^19^. There is an emerging need for advanced AD detection and prediction models that can serve as a helping hand for medical practitioners to diagnose or detect the disease earlier and more accurately^5,19,20^. These models can be used to build the inference engines of an AD clinical decision support system (CDSS). However, as far as we know there is no CDSS for AD diagnosis and progression detection ready to use at primary care. In this context, two lines of research have been conducted to address the previous challenges: (1) deep learning (DL) techniques which are able to automatically learn complex, non-linear data transformations that optimize performance metrics^21–23^; and (2) regular machine learning (ML) techniques, especially support vector machine (SVM) and random forest (RF)^7,24–28^.

Unfortunately, all these previous studies focused mainly on improving the system performance while neglecting interpretability issues. Accordingly, although these studies achieved tremendous advances in prediction, they are not expected to be acceptable in the medical environment. There exists a significant gap between academic research outcomes and their effective utilization in medical practice due to several reasons^20^. The entire patient medical history must be considered to achieve intuitive, stable, and robust decisions^20^. Most DL-based methods only concentrate on analysis of neuroimaging, i.e., Magnetic Resonance Imaging (MRI) and Positron Emission Tomography (PET). Nevertheless, Oxtoby and Alexander^29^ asserted that neuroimaging is not sufficient for AD diagnosis and studying its progression. Furthermore, it is frequently the case that physicians do not rely on the latest technical approaches and methodologies (e.g., DL and RF), despite their high accuracy^18^, because complex model performance and explainability are in apparent conflict- i.e., the search for a good performance-explainability trade-off is required. Most of these approaches and schemes are inherently opaque, not understandable, and unable to easily answer the following straightforward questions. Why/how has it reached a specific decision, and why/how is it medically relevant^30^? The patterns learned from datasets by using complex ML algorithms do not necessarily carry correct and comprehensible knowledge. Thus, medical experts do not trust decisions provided by black-box models without comprehensive and easy-to-understand explanations^31^. For these reasons, the ML techniques employed in the clinical domain normally do not consider sophisticated models, resorting instead to simpler and interpretable (e.g., linear) models at the expense of accuracy^32^. Many studies have tried to open the black box of complex models and provide an explanation of their decisions, either by understanding how the models work or by explaining their decisions^33^. This new trend is called accountable, transparent, actionable, or explainable Artificial Intelligence (AI), or just XAI for short. Explainability is the ability of ML algorithms to (mathematically) explain or justify their results using terms which are understandable to humans.

A CDSS should be based only on ML models that provide a balance between accuracy and explainability. These models are expected to provide sufficient information about the relationship between input features and predictions, and to allow users to answer questions like the following. Which features are the key players in the prediction of interest? Why am I deemed as normal/MCI/AD in the medical diagnosis? For these reasons, the second line of research introduced above (i.e., a CDSS based on regular ML techniques) seems more intuitive and medically acceptable. Regular ML techniques involving linear models and rule-induction algorithms (e.g., a decision tree [DT]^34^ or a fuzzy rule-based system [FRBS]^35,36^) are usually preferred when the priority is to generate explainable models^18,37–39^. Unfortunately, these models are not always accurate enough^40^. One solution is to use an accurate algorithm as an oracle for the classification purpose, and a collection of carefully designed interpretable models (which behave as digital twins of the oracle, i.e., they imitate the classification behavior of the oracle) as candidates to generate explanations of the output provided by the oracle^41^. The other solution is to open the black box and collect the explanations from the opaque model itself. For example, some studies have extracted interpretable rules from black-box models such as neural networks and SVMs^42,43^. In the case of RF, Brieman^44^ asserted that it is an A + predictor for performance, but rates an F on interpretability. More recently, some authors have shown how the behavior of RF can be interpreted to some degree^31,45^. There exists no study in the literature, which use the RF algorithm in the core of an explainable CDSS system for AD diagnoses and progression detection.

Despite the current research effort, AD detection and progression prediction are still openly challenging problems, due to the limited accuracy and limited explainability of existing solutions. The medical domain requires both accurate and explainable AI models. In this paper, we therefore develop a new RF-based explainable AD detection and progression prediction model. Our contributions are as follows:

1. We demonstrate how to retain interpretability, even when a complex ensemble model like RF is used. The objective of this approach is two-fold: (1) To illustrate the development and validation process of a two-layer computational framework for diagnosing AD patients and predicting pMCI within three years from baseline diagnosis; and (2) to describe how to provide detailed and multiple explanations for the ML decisions. The resulting model provides physicians with a good balance between accuracy and explainability.
2. We build accurate ML ensemble classifiers based on RF for the two layers; utilizing multimodal AD datasets collected from the Alzheimer’s Disease Neuroimaging Initiative (ADNI). We employ a comprehensive list of modalities to diagnose AD and predict its progression, in agreement with a physician who was taken as domain expert.
3. We build 22 explainers, based on a set of interpretable ML techniques (i.e. DT and FRBS), ready to explain to physicians the outcome of the two-layer framework. This reverse engineering method is called a black-box outcome explanation^33^. All explainers’ decisions compatible with RF decisions are used to provide physicians with a pool of plausible explanations. In analogy with a panel of experts who may have different experience and background, each explanation comes from a different explainer which pays attention to the most relevant features for different modalities, and comes along with information about the reliability of the explainer in terms of its accuracy. The consistency and coherence of such explanations are validated by domain experts and ranked according to their explainability-accuracy trade-off. Moreover, they are mapped to a human-friendly language for easy understanding.
4. We provide physicians with some insights into driving factors of our prediction model from multiple points of view including natural language, visualization, and feature importance based on SHapley Additive exPlanations (SHAP).

The rest of this paper is organized as follows. Section 2 presents and discusses the main reported results. Section 3 introduces the datasets used and goes in depth with technical details of the proposed method. Section 4 concludes the paper.

## Results and discussion

### Identification of informative AD features

To reduce computational complexity that comes with the high dimensionality nature of the ADNI, we selected the most relevant feature set using automatic feature selection strategy. For each layer, the full dataset is stratified and randomly divided into a model development set [$S1$] and a testing set [$S2$]. $S1$ and $S2$ are filtered to create the best feature sets $MS1$ and $MS2$, respectively (see the Feature Selection and Modeling Approach Section; in Material and Methods). The new sets are used to tune, train, and tests the utilized ML models. Training and tuning of ML models is done with cross-validation over *MS1* while *MS2* is reserved to provide readers with final test evaluation, mainly regarding some illustrative examples of the explainability of the proposed framework.

**Figure 1:**
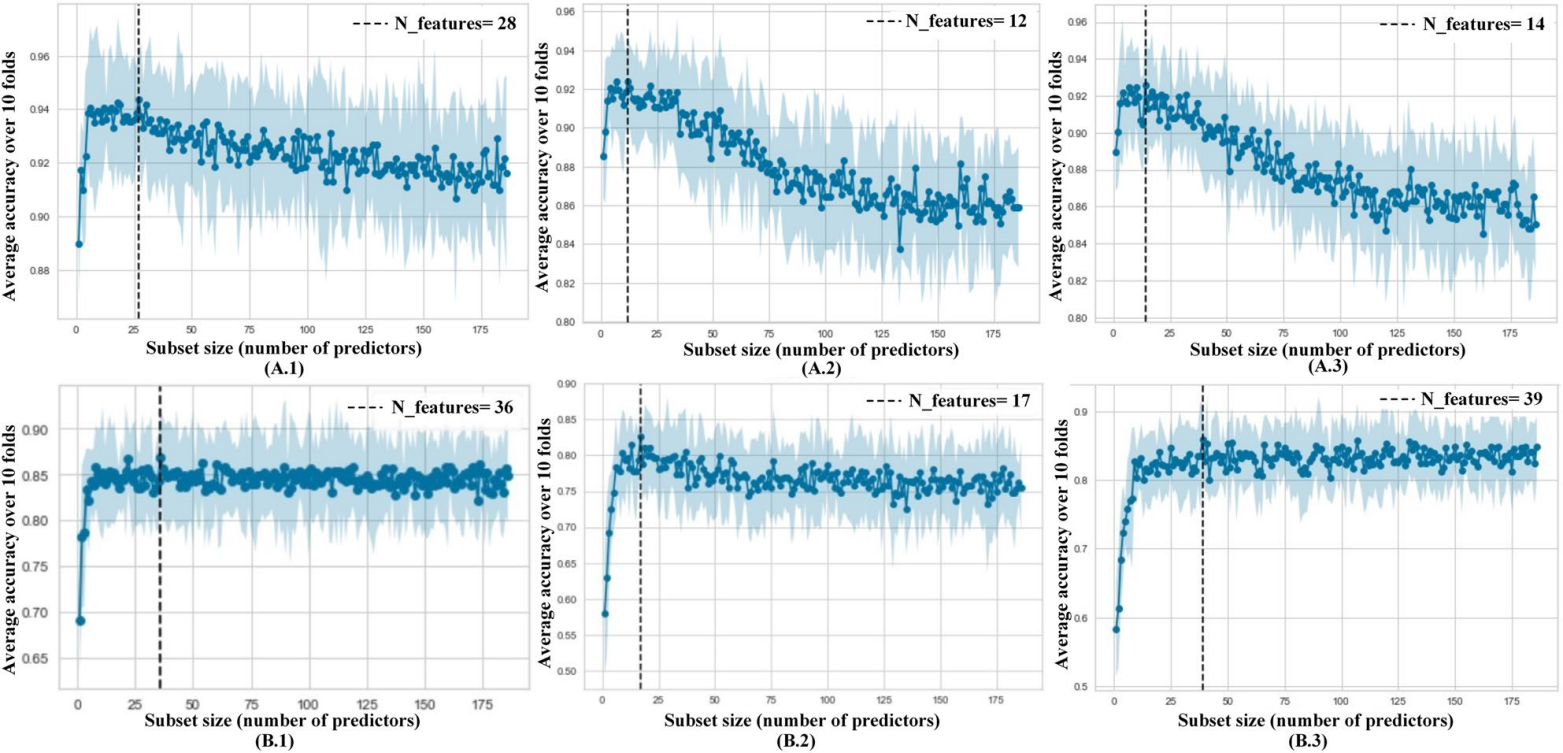
Selected features for both layers based on three different techniques of SVM, RF, and GB. The first row is for the first layer, and the second row is for the second layer.

Figure 1A shows the performance of different subset sizes assessed with RF-RFE (A.1), SVM-RFE (A.2), and GB-RFE (A.3) for the first layer. For different combinations of features, the accuracy from RF, SVM, and GB was measured, and the subset of features with the best performance was detained. As summarized in Table 1, for RF-RFE, we obtained a combination of 28 features [cognitive scores (8), genetics (5), lab tests (1), demographics (3), MRI (2), neuropsychological battery (6), and PET (3)] to attain the highest predictive accuracy of 94.4% (see Supplementary File [part 2], Table T1). Because the optimal subset of features derived using the RFE-RF approach yields the maximum accuracy, we utilized it for training the classification model. These features form about 15% of the whole feature set. Inspired by^20^, the features selected with RF-RFE are clustered into six modality kinds: (1) cognitive scores (CS) [eight features]; (2) neuropsychological battery (NB) [six features]; (3) MRI [two features], (4) PET [three features], (5) genetics [five features], and (6) medical history (MH) (lab test and demographics) [four features]. It is worth noting that the selected features based on RF-RFE are the most discriminant and informative features for the current classification problem (*P* < 0.05, Kruskal–Wallis test). The list of non-selected features does not add discriminative values with RFE; however, as asserted by our domain experts, many of these neglected features could provide additional knowledge to understand the made decisions (i.e., they include critical values for model’s explainability in accordance with physicians’ intuition and background). The different modalities were screened to investigate whether a cost-effective and non-invasive subset of features have a higher discriminative power than the whole dataset.

**Table 1:** Performance of the RFE for the two layers.

Layer	Model	No. of features	Accuracy (%)
First Layer	**RF-RFE**	**28**	**94.40**
	SVM-RFE	12	92.40
	GB-RFE	14	92.60
Second Layer	**RF-RFE**	**36**	**86.80**
	SVM-RFE	17	82.60
	GB-RFE	39	86.00

Figure 1B shows the performance of different subset sizes assessed with RF-RFE (B.1), SVM-RFE (B.2), and GB-RFE (B.3) for the second layer. The accuracy of RF, SVM, and GB was calculated for different combinations of features, and the subset of features with the best performance was taken. Similar to the First Layer, the RFE algorithm attained a higher performance when combined with RF than GB and SVM. With RF-RFE (see Table 1), the combination of 36 features [cognitive scores (7), genetics (5), lab tests (6), demographics (1), MRI (5), neuropsychological battery (7), PET (3), and vital signs (2)] achieves the highest predictive accuracy at 86.8%. Accordingly, we used the RF-RFE feature set for training the classification model. These features formed about 19% of the total feature set, (see Supplementary File [part 2], Table T1). Furthermore, we grouped this list of features into five modality types: (1) cognitive and functional assessments (CFA) (CS and NB), (2) MRI, (3) PET, (4) genetics, and (5) MH (lab tests, age, and vital signs). As with the First Layer, we analyzed the performance of different RF classifiers constructed using each modality (as well as their combinations).

### First layer: early AD detection performance

The First Layer in the framework is responsible for detecting AD patients from CN and MCI patients. To determine the smallest number of features that produces the most accurate results, we performed a set of experiments using different combination of modalities. Table 2 shows the performance obtained for the multiclass classification problem (i.e., CN, MCI, and AD) by using the whole training dataset and different combinations of six selected modalities (CS, NB, MRI, PET, MH, and genetics) and RF classifier (see the Random Forest for Classification Section). The models’ performance has been evaluated using the area under the receiver operating characteristic curve (AUC), precision, recall, accuracy (AC), and F1-score (F1) metrics (see the Model Performance Evaluation Metrics; in Material and methods). When the whole feature set is used, the model has a multiclass classification accuracy (MCA) of 93.42 ± 2.73% based on tenfold CV. We can see that the CS modality has the highest accuracy (MCA = 92.00 ± 2.26%), compared to other single modalities. As a result, the CS modality was combined with other modalities to test the improvement in the model performance. Please note that, although adding more features could increase the model’s confidence, it also adds additional noises. The two-modality combination CS + NB improved the CS accuracy by about 1%, i.e., MCA = 93.00 ± 2.61%. We notice that the standard deviation of the combined CS + NB data slightly increased compared to the CS dataset alone, but still it is less than the standard deviation of the models based on the whole dataset. After integration of the CS + NB modality with the other types of data, the genetics data improved the accuracy of the system to 93.95 ± 2.30%. We discover that the RF shows more confidence based on the CS + NB + Genetics dataset than CS + NB dataset. This is because its performance has lower standard deviation. The resulting modality of CS + NB + Genetics was tested by combining it with MRI, PET, and MH. However, the performance was not enhanced, and the models become noisier. As a result, the combination of CS, NB, and Genetics was selected as the one producing the best performance.

**Table 2:** Random Forest performance validation for detecting AD patients based on *tenfold cross-validation*.

Modalities	Precision (%)			Recall (%)			MCA (%)	MCF (%)
	CN	MCI	AD	CN	MCI	AD		
**All modalities**	**98.83 ± 1.33**	**90.91 ± 2.55**	**92.41 ± 1.99**	**96.21 ± 1.11**	**95.45 ± 2.00**	**86.61 ± 2.10**	**93.42 ± 2.73**	**93.39 ± 2.19**
*CS*	*99.60* ± *0.17*	*89.76* ± *1.99*	*88.51* ± *1.04*	*93.94* ± *0.18*	*93.64* ± *1.08*	*87.03* ± *2.09*	*92.00* ± *2.26*	*92.08* ± *2.00*
NB	84.86 ± 1.32	74.11 ± 2.06	77.93 ± 2.00	80.68 ± 1.09	80.68 ± 1.92	69.46 ± 2.01	77.83 ± 2.33	77.94 ± 2.10
MRI	45.19 ± 2.46	47.16 ± 4.34	48.99 ± 3.01	46.21 ± 2.41	50.91 ± 3.99	40.59 ± 3.31	46.99 ± 4.01	46.50 ± 3.91
PET	61.59 ± 1.89	65.06 ± 2.22	70.35 ± 3.03	70.45 ± 1.40	61.36 ± 3.50	66.53 ± 2.87	65.23 ± 2.98	65.89 ± 2.11
Genetics	61.28 ± 2.60	58.82 ± 3.33	54.90 ± 2.42	68.94 ± 2.00	59.09 ± 3.91	46.86 ± 2.00	58.75 ± 3.11	58.31 ± 2.89
MH	46.60 ± 1.42	50.08 ± 2.91	29.29 ± 1.67	36.36 ± 1.79	67.95 ± 3.82	17.15 ± 1.77	46.22 ± 2.99	41.22 ± 3.00
*CS* + *NB*	*99.22* ± *1.09*	*90.65* ± *2.06*	*90.79* ± *0.90*	*95.83* ± *1.11*	*94.77* ± *2.31*	*86.61* ± *2.90*	*93.00* ± *2.61*	*92.97* ± *2.25*
CS + MRI	99.20 ± 2.11	89.42 ± 3.62	89.57 ± 2.39	93.94 ± 2.70	94.09 ± 3.59	86.19 ± 3.20	92.05 ± 3.99	92.06 ± 4.01
CS + PET	99.20 ± 2.01	89.25 ± 2.99	89.87 ± 2.51	94.32 ± 2.22	94.32 ± 3.87	85.36 ± 2.91	92.05 ± 3.33	92.05 ± 3.41
CS + Genetics	99.20 ± 1.99	89.01 ± 2.53	89.08 ± 2.33	93.94 ± 1.83	93.86 ± 3.01	85.36 ± 2.43	91.73 ± 2.91	91.74 ± 3.08
CS + MH	98.43 ± 1.55	89.15 ± 4.01	89.04 ± 1.92	94.70 ± 1.77	93.41 ± 4.31	84.94 ± 3.91	91.62 ± 4.00	91.61 ± 3.81
CS + NB + MRI	99.22 ± 2.05	90.87 ± 3.91	91.19 ± 2.71	96.21 ± 2.09	95.00 ± 3.61	86.61 ± 2.99	93.21 ± 3.40	93.18 ± 3.47
CS + NB + PET	99.22 ± 1.99	91.45 ± 2.06	90.83 ± 2.90	96.97 ± 1.78	94.77 ± 3.01	87.03 ± 2.72	93.42 ± 2.97	93.38 ± 2.61
CS + NB + Genetics*	**99.22 ± 1.01**	**91.72 ± 2.01**	**92.54 ± 0.91**	**96.21 ± 1.00**	**95.68 ± 2.00**	**88.28 ± 1.81**	**93.95 ± 2.30**	**93.94 ± 2.07**
CS + NB + MH	99.22 ± 2.01	91.50 ± 3.87	92.07 ± 2.33	96.59 ± 2.33	95.45 ± 4.10	87.45 ± 3.83	93.74 ± 4.00	93.71 ± 3.61
CS + NB + Genetics + MRI	99.22 ± 2.30	91.50 ± 4.10	92.11 ± 2.97	96.21 ± 2.22	95.45 ± 4.20	87.87 ± 4.86	93.74 ± 4.01	93.72 ± 4.44
CS + NB + Genetics + PET	99.61 ± 2.03	90.46 ± 2.06	90.27 ± 3.04	96.59 ± 1.83	94.77 ± 3.31	85.36 ± 2.77	92.89 ± 2.99	92.84 ± 3.20
CS + NB + Genetics + MH	99.22 ± 2.22	91.34 ± 4.39	92.83 ± 2.31	96.97 ± 2.40	95.91 ± 4.90	86.61 ± 3.09	93.85 ± 4.30	93.81 ± 4.06
CS + NB + Genetics + MH + MRI	99.22 ± 2.30	90.93 ± 4.55	92.41 ± 3.60	96.21 ± 2.32	95.68 ± 4.24	86.61 ± 3.95	93.53 ± 4.32	93.51 ± 3.97
CS + NB + Genetics + MH + PET	98.84 ± 1.89	91.29 ± 3.41	92.04 ± 2.87	96.59 ± 2.00	95.23 ± 3.05	87.03 ± 2.78	93.53 ± 3.11	93.50 ± 3.01

MCA: multiclass classification accuracy, MCF: multiclass F1 score; Asterisk ( ∗): is the subset of features with the best predictive performance; italic text is the best of single and pairs of modalities.

The next step is to show the generalization capability of the proposed model. As shown in Table 3, we observe the same trend already shown in Table 2. Once again, the combination of NB, CS, and Genetics again achieved the best performance.

**Table 3:** Random Forest performance testing for detecting AD patients ($MS2$ *test dataset*;10% of the original data).

Modalities	Precision (%)			Recall (%)			MCA (%)	MCF (%)
	CN	MCI	AD	CN	MCI	AD		
**All modalities**	**100.0**	**86.54**	**96.30**	**86.67**	**97.83**	**89.66**	**92.38**	**92.81**
CS	100.0	71.43	93.75	86.61	97.83	51.72	81.90	83.29
*NB*	*85.29*	*81.63*	*95.45*	*96.60*	*86.96*	*72.41*	*85.71*	*86.39*
MRI	50.31	48.28	72.22	40.01	91.30	44.83	52.38	42.61
PET	50.00	55.22	73.53	36.67	80.43	86.21	60.95	58.66
Genetics	66.67	49.28	40.21	80.00	73.91	35.33	55.24	44.09
MH	33.33	52.54	38.46	36.67	67.39	17.24	44.76	40.93
*NB* + *CS*	*100.0*	*84.91*	*96.15*	*86.67*	*97.83*	*86.21*	*91.43*	*91.93*
NB + MRI	73.68	79.55	95.65	93.33	76.09	75.86	80.95	82.36
NB + PET	86.21	75.93	95.45	83.33	89.13	72.41	82.86	83.69
NB + Genetics	87.50	83.67	95.83	93.33	89.13	79.31	87.62	88.12
NB + MH	87.50	77.36	95.00	93.33	89.13	65.52	83.81	84.59
NB + CS + MRI	100.0	83.64	100.0	86.67	100.0	82.76	91.43	92.12
NB + CS + PET	100.0	82.14	100.0	86.67	100.0	79.31	90.48	91.27
NB + CS + Genetics*	**100**.0	**86.79**	**100**.0	**86.67**	**100**.0	**89.66**	**93.33**	**93.82**
NB + CS + MH	100.0	86.54	96.30	86.67	97.83	89.66	92.38	92.81
NB + CS + Genetics + MRI	100.0	85.19	100.0	86.67	100.0	86.21	92.38	92.97
NB + CS + Genetics + PET	100.0	85.19	100.0	86.67	100.0	86.21	92.38	92.97
NB + CS + Genetics + MH	100.0	86.79	100.0	86.67	100.0	89.66	93.33	93.82
NB + CS + Genetics + MH + MRI	100.0	86.54	96.30	86.67	97.83	89.66	92.38	92.81
NB + CS + Genetics + MH + PET	96.15	84.62	96.30	83.33	95.65	89.66	90.48	90.93

MCA: multiclass classification accuracy, MCF: multiclass F1 score; Asterisk ( ∗): is the subset of features with the best predictive performance; italic text is the best of single and pairs of modalities.

### Second layer: AD progression prediction performance

The Second Layer in our framework optimizes a binary classification problem to predict the progression to AD within three years from baseline (i.e., sMCI versus pMCI). This classifier is first validated using tenfold CV on the $MS1$ dataset. As shown in Table 4 with bold typeface, the best performance of this model was observed for the combined CFA, PET, Genetics, and MRI data, i.e. Precision = 88.07 ± 0.70%, Recall = 86.08 ± 1.30%, Accuracy = 87.09 ± 0.80%, F1-score = 87.08 ± 0.90%, and AUC = 87.08 ± 0.80%. In addition, this model achieved the lowest variance in performance compared to all models based on other combinations and the whole feature space. Regarding single modalities, CFA achieves the best performance (see Table 4). In addition, cognitive scores and neuropsychological battery are usually considered in clinical practice. Models built using either MH or PET alone achieved the worst performance and were noisy. Based on the results from single modalities, we combined the best CFA model with each of the other modalities to see if the performance may be improved or not.

**Table 4:** Random Forest performance validation for predicting whether MCI subjects will progress to AD or not (*tenfold cross-validation*; Second Layer).

Modalities used	Precision (%)	Recall (%)	Accuracy (%)	F1-score (%)	AUC
All	87.12 ± 1.52	81.31 ± 2.00	84.18 ± 1.77	83.21 ± 2.43	84.17 ± 1.99
*CFA*	*82.14* ± *1.40*	*84.19* ± *1.90*	*82.16* ± *1.60*	*83.15* ± *1.50*	*82.15* ± *1.50*
MRI	75.23 ± 1.88	72.25 ± 1.59	71.18 ± 2.01	72.17 ± 1.92	71.18 ± 1.89
PET	68.22 ± 2.22	68.53 ± 1.98	68.25 ± 1.99	66.39 ± 1.99	68.24 ± 2.01
Genetics	73.11 ± 1.73	68.36 ± 1.72	70.14 ± 1.79	69.24 ± 1.89	70.13 ± 1.80
MH	58.16 ± 4.60	52.22 ± 4.90	55.15 ± 3.73	54.19 ± 3.76	55.15 ± 3.76
CFA + MRI	83.11 ± 2.31	84.22 ± 2.20	83.14 ± 2.51	83.15 ± 2.26	83.14 ± 2.22
*CFA* + *PET*	*85.17* ± *1.70*	*84.29* ± *2.90*	*84.19* ± *1.90*	*84.21* ± *2.10*	*84.19* ± *1.90*
CFA + Genetics	82.14 ± 1.91	81.27 ± 1.98	81.16 ± 1.93	81.17 ± 1.96	81.16 ± 1.95
CFA + MH	84.16 ± 3.77	82.23 ± 4.60	82.17 ± 3.80	83.18 ± 3.33	82.17 ± 3.80
CFA + PET + MRI	86.09 ± 2.10	84.23 ± 2.30	84.11 ± 2.15	85.14 ± 2.22	85.11 ± 2.15
*CFA* + *PET* + *Genetics*	*90.11* ± *1.50*	*83.21* ± *2.21*	*86.08* ± *1.04*	*85.11* ± *2.00*	*86.08* ± *1.05*
CFA + PET + MH	86.09 ± 3.45	84.17 ± 4.20	84.08 ± 3.71	85.09 ± 4.01	84.08 ± 3.72
CFA + PET + Genetics + MRI*	**88.07 ± 0.70**	**86.13 ± 1.30**	**87.08 ± 0.80**	**87.09 ± 0.90**	**87.08 ± 0.80**
CFA + PET + Genetics + MH	86.09 ± 3.51	86.13 ± 4.70	86.08 ± 3.32	86.08 ± 3.99	86.08 ± 3.36

BA: Balanced accuracy. Asterisk ( ∗): is the subset of features with the best predictive performance. Performance: Mean ± standard deviation.

The addition of PET data improves the predictive performance of our model because PET data provide complementary information about disease progression. The combination of CFA and PET modalities achieves the best performance compared to combinations of other pairs of modalities. However, the resulting model is less confident compared to the model based on CFA alone. This is probably because the PET modality added noise to the combined set. In addition, the CFA + PET modality achieved the smallest variance compared to other two modalities combinations. To check for possible improvement in model performance, the CFA + PET feature set was combined with each of the MRI, Genetics, and MH modalities. The multimodality of CFA, PET, and Genetics enhances the performance of progression prediction by about 2%, compared to the combined CFA and PET modality. In addition, the resulting model is more stable compared to the CFA + PET-based model. This is in accordance with the fact that medically, Amyloid β, PTAU, and TAU are critical biomarkers to monitor the progression of AD^46–51^. Finally, we check the effect of combining MRI and MH with the rest of the modalities (CFA, PET, and Genetics). Again, integrating MRI brain volume features (including the hippocampus, ICV, and others) improves the model accuracy by about 1%. MRI volume features provide vital information for effective prediction of AD progression. According to our domain experts, we believe this is medically promising because it is critical to integrate MRI features in order to measure possible AD progression. With the unseen data in $MS2$ we verify the good generalization of the generated models that we already observed with tenfold CV (see Table 5).

**Table 5:** Random Forest performance measures for AD progression prediction of MCI subjects based on CFA, MRI, PET, genetics, and MH modalities ($MS2$ *test dataset*; Second Layer).

Modalities used	Precision (%)	Recall (%)	Accuracy (%)	F1-score (%)	AUC
All	87.50	87.50	87.76	87.75	0.953
*CFA*	*91.30*	*87.50*	*89.80*	*89.81*	*0.926*
MRI	56.67	70.83	59.18	59.66	0.691
PET	72.73	66.67	71.43	71.44	0.812
Genetics	76.00	79.17	77.55	77.58	0.787
MH	57.14	50.00	57.14	57.07	0.562
CFA + MRI	90.91	83.33	87.76	87.86	0.903
*CFA* + *PET*	*95.45*	*87.50*	*91.84*	*91.95*	*0.955*
CFA + genetics	84.00	87.50	85.71	85.75	0.926
CFA + MH	91.30	87.50	89.80	89.81	0.918
CFA + PET + MRI	88.00	91.67	89.80	89.83	0.949
*CFA* + *PET* + *genetics*	*91.67*	*91.67*	*91.85*	*91.83*	*0.956*
CFA + PET + MH	87.50	87.50	87.76	87.75	0.943
CFA + PET + genetics + MRI*	*91.70*	*91.70*	*91.86*	*91.84*	*0.963*
CFA + PET + genetics + MH	87.50	87.50	87.76	87.75	0.948

Asterisk ( ∗): is the subset of features with the best predictive performance.

### Comparison with other classifiers

Recently, Travers et al.^21^ provided a comprehensive survey of DL techniques in biology and medicine. In this context, Choi and Jin^22^ utilized a convolutional neural network (CNN) to detect pMCI cases based on positron emission tomography (PET) images. Spasov et al.^23^ proposed a multimodal DL classification model for AD progression detection based on the late fusion of magnetic resonance imaging (MRI), demographic, neuropsychological, and apolipoprotein E (APOE) e4 genetic data.

In addition, many AD studies have considered a single modality, especially MRI, to make a binary classification of sMCI versus pMCI^52,53^. Li et al.^54^ used five cognitive scores with a Cox linear regression model to build two prognostic models of AD. Moradi et al.^27^ achieved an area under the curve (AUC) of 0.77 in discriminating pMCI from sMCI based on RF and MRI data only; after fusing MRI features with baseline cognitive scores and age, they achieved an AUC of 0.90 for the same problem. Jin et al.^55^ used a Bayesian network to analyze multimodal data from ADNI data including demographics, MRI, PET, neuropsychometrics tests, and genotypes. It is worth noting that RF^56,57^ is an ensemble classifier that can provide more accurate predictions than other ML techniques. Fernandez-Delgado et al.^40^ evaluated 179 classifiers using different UCI datasets, and concluded that RF outperforms other classifiers, including SVMs and neural networks. RF works well with a mixture of quantitative and categorical features, and unlike SVM, it handles multiclass problems natively. RF is able to learn wide datasets with a very large number of features, compared to the number of cases. RF has been used intensively in the AD domain^26,57,58^. For example, Ramírez et al.^58^ proposed an ML model to predict MCI from normal patients. This model is based on feature standardization, analysis of variance feature selection, partial least squares feature dimension reduction, and an ensemble of one vs. rest RF classifiers. The model achieved accuracy of 56.25% based on MRI data.

To verify the goodness and robustness of our approach in each layer, we compared the performance of the RF models with other predictive models, namely the SVM, KNN, Naïve Bayes (NB), and DT models. For each layer, we use the selected features of RFE. For each selected algorithm, we tuned its hyperparameters the same way we tuned the RF algorithm. The results of the best performing parameters are shown in Tables 6 and 7. Our proposal outperforms the rest of classifiers. It is worth noting that we did not compare our model with the artificial neural network approach because they achieved really bad performance in preliminary experiments, mainly due to the small size of the used datasets. In other words, our data is not big enough for training and testing the state-of-the-art DL architectures.

**Table 6:** Comparison of different classifiers ($MS2$ *test dataset*; First Layer).

Classifier	Precision (%)			Recall (%)			MCA (%)	MCF (%)
	CN	MCI	AD	CN	MCI	AD		
SVM	100.0	91.43	83.87	96.67	89.13	89.66	91.43	91.74
KNN	65.71	59.57	73.91	76.67	60.87	58.62	64.76	65.89
DT	100.0	86.54	96.30	86.67	97.83	89.66	92.38	92.81
NB	90.32	92.68	84.85	93.33	82.61	96.55	89.52	90.05
RF (our model)*	**100.0**	**86.79**	**100.0**	**86.67**	**100.0**	**89.66**	**93.33**	**93.82**

MCA: multiclass classification accuracy, MCF: multiclass F1 score; Asterisk ( ∗): is the model with the best predictive performance.

**Table 7:** Comparison of different classifiers ($MS2$ *test dataset*; Second Layer).

Modalities used	Precision	Recall	Accuracy	F1-score	AUC
SVM	81.82	75.00	79.59	79.64	0.867
KNN	83.33	83.33	83.67	83.66	0.869
DT	85.71	75.00	81.63	81.82	0.850
NB	84.00	84.00	83.67	83.67	0.907
RF (our model)*	**87.50**	**87.50**	**87.76**	**87.75**	**0.953**

Asterisk ( ∗): is the model with the best predictive performance.

### Models explainability

#### Explainability based on random forest internal logic

Based on SHAP explainers, we calculate feature contributions of RF models (see the Explainability Capabilities Section; in Material and methods). Figure S1 in Supplementary File (part 2) shows this rank for each class in each layer. The most influential feature for the First Layer is CDRSB followed by MMSE, and the lowest feature is TRABSCOR_PartBTimeToComplete from the neuropsychological battery group (see Supplementary File [part 2], Table T2). For the Second Layer, FAQ plays the main role followed by ADNI_MEM, and Trail4Total has the lowest impact (see Supplementary File [part 2], Table T2). According to our domain experts, it is medically intuitive for cognitive scores to play the main role in detecting AD patients. However, for progression detection, we can see that Hippocampus and MidTerp volumes from MRI images also play significant role, in addition to FDG and SROI from PET images. Table 8 summarizes the sensitivity of the explainer to the different feature values for both layers. For further details about these features and terminologies, readers are invited to see the Supplementary File (part 2) and ADNI at http://adni.loni.usc.edu.

**Table 8:** Examples of the relationship between features and class prediction.

First Layer	Most influential feature	CDRSB	Clinical dementia rating sum of box score
	Lowest important feature	TRABSCOR PartBTimeToComplete	Neuropsychological battery’s TRABSCOR trail making test (part B—time to complete)
	The best feature for AD class	MMSE	Mini-Mental State Examination
	The best feature for CN and MCI classes	CDRSB	Clinical dementia rating sum of box score
	$\uparrow$ CDRSB, $\downarrow$ ADNI_MEM	$\uparrow$ risk for the AD class	Sensitivity of the AD class to this list of features
	$\downarrow$ CDRSB, $\uparrow$ ADNI_MEM, $\uparrow$ DigitalTotalScore, $\uparrow$ MOCA	$\uparrow$ chance for the CN class	Sensitivity of the CN class to this list of features
	$\downarrow$ CDRSB, $\uparrow$ FAQ,$\uparrow$ MOCA, $\uparrow$ CDGLOBAL, $\downarrow$ ADNI_MEM	$\downarrow$ risk for the MCI class	Sensitivity of the MCI class to this list of features

Second Layer	Most influential feature	ADNI_MEM	ADNI_MEM is composite logical memory score for the8 longitudinal changes in memory
	Lowest important feature	Trail4Total	Neuropsychological Battery AVTOT4 feature
	$\uparrow$ FAQ, $\uparrow$ RAVLT_immediate	$\uparrow$ chance for the sMCI class	Relationship between RAVLT_immediate and sMCI class
	$\uparrow$ FAQ, $\uparrow$ RAVLT_immediate	$\downarrow$ risk for the pMCI class	Relationship between FAQ and RAVLT_immediate and pMCI class
	$\uparrow$ ADAS 13, $\uparrow$ ADNI_MEM, $\downarrow$ FDG, $\downarrow$ MOCA	$\uparrow$ risk for the pMCI class	Relationship between ADAS, ADNI_MEM, FDG, and MOCA and pMCI class

#### Explainability of the behavior of individual features

**Figure 2:**
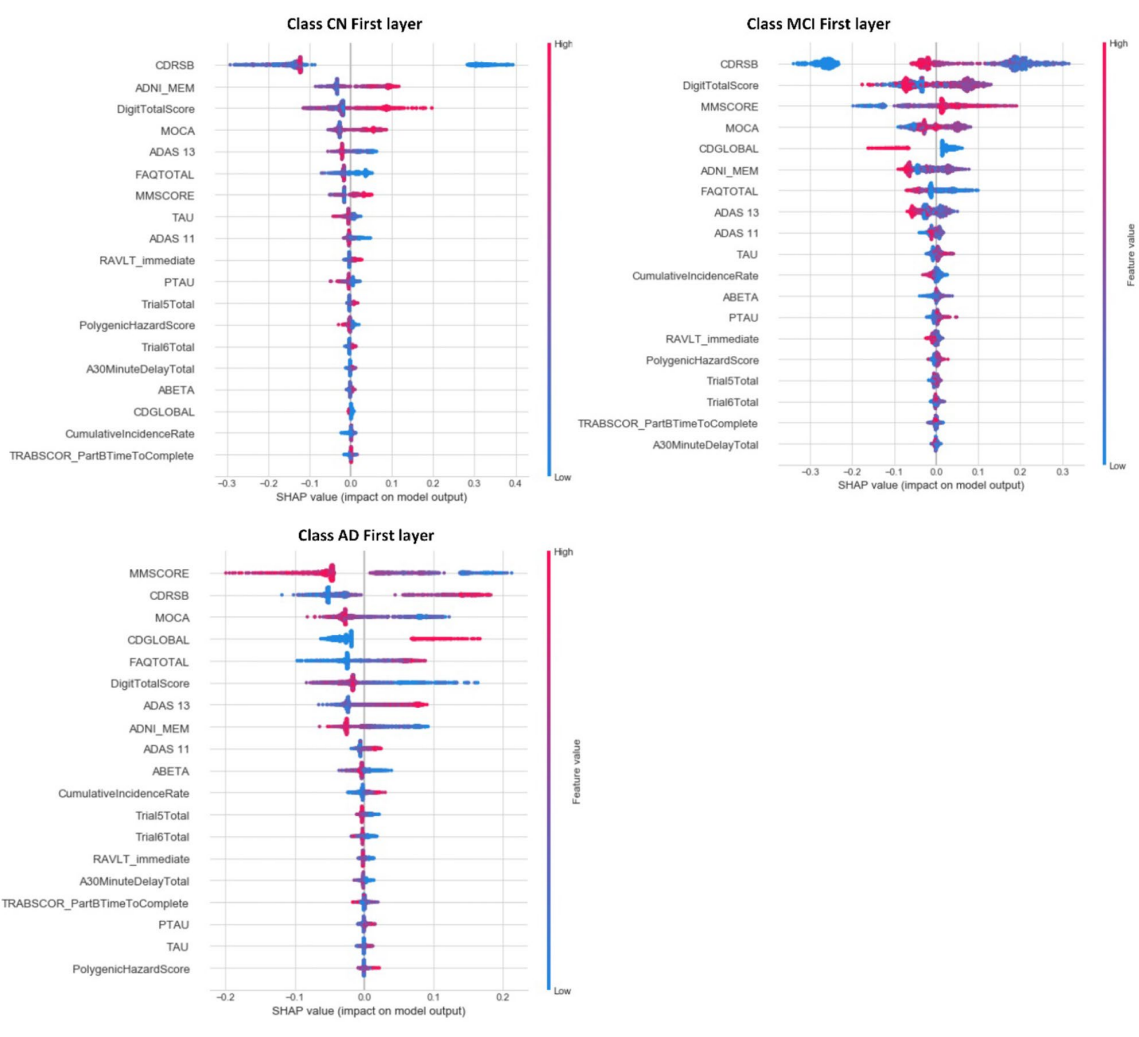
SHAP summary plots for the first layer. The upper left figure represents the CN class, the upper right figure represents the MCI class, and the second row represents the AD class.

The global feature importance gives an abstract view about the role of each feature, but we cannot know the direction of these effects. For example, we cannot know if a high value for CDRSB will increase the probability of selecting the AD, MCI, or CN class. Using SHAP summary plots, we are able to analyze the behavior of our XAI framework with respect to different values of features. Figure 2 shows the summary plots for every class in the first layer. Each dot represents the impact on a particular class of a particular feature for a given instance, and it is colored according to what magnitude of the value contributes to the model impact. The color represents the feature value (red = high, blue = low). We notice a different order for each class.

In the First Layer, we find that MMSE is more significant than CDRSB for the AD class, but CDRSB has the highest impact on the CN and MCI classes, see Table 8. The model shows a high degree of non-linearity because the impacts of many features are spread across relatively wide ranges. We notice that the high values of CDRSB have great positive impact on the model for predicting the AD class, meaning CDRSB is a factor that increases AD risk. For the CN class, low values of CDRSB have extreme positive impact on the model. In contrast, high values of ADNI_MEM, DigitalTotalScore, and MOCA have a positive impact on predicting the CN class. For the MCI class, low values of CDRSB have an extreme negative impact on the model. The CDGLOBAL feature is less critical than MOCA for the MCI class. However, in some cases, high values of this feature have a more negative impact on MCI cases than MOCA. The same happens for FAQ, where low values have a more positive impact on a system decision for the MCI class than MOCA and CDGLOBAL. We noticed that AD and MCI classes are related to negative values of ADNI_MEM, but CN is related to positive values. In addition, by plotting the impact of a feature on every sample, we can detect the impact of outliers. For example, in the case of the picture related to AD, although CDGLOBAL is not the most important feature globally, it is critical for a subset of patients. This is indicated by the long-tailed distribution to the right. Again, the same situation applies to the DigitalTotalScore feature for the AD class.

**Figure 3:**
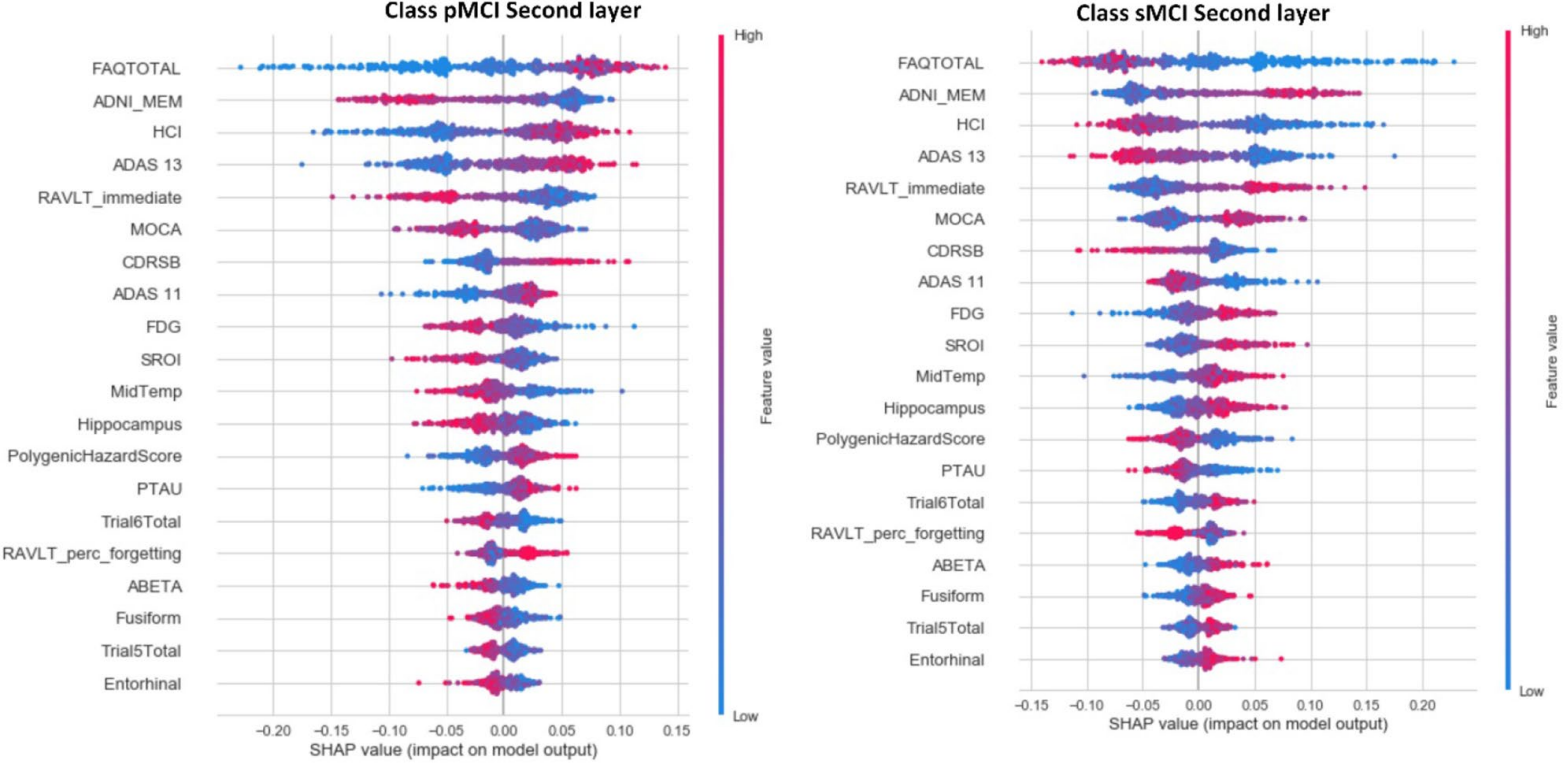
SHAP summary plots for the second layer. The left figure shows the pMCI class, and the right figure shows the sMCI class.

In the Second Layer, although HCI is globally less significant than ADNI_MEM for both sMCI and pMCI, in a subset of patients, this feature has more impact than ADNI_MEM, see Table 8 and Fig. 3. The same is true for CDRSB in relation to MOCA for the pMCI class, and ADAS 11 in relation to CDRSB for the sMCI class. A feature with a longer tail to the right means it has a greater positive influence, and vice versa. As a result, understanding the detailed role of each feature alone and in combination with other features is of critical importance. For example, large values of RAVLT_immediate positively impact the model toward selecting the sMCI class, but negatively impact towards the pMCI class. FAQ is the most important feature for both classes, followed by ADNI_MEM, HCI, and ADAS 13. The two classes show symmetric behaviors for all features. It is clear that low values of FAQ negatively affect the prediction of pMCI class, but they have the largest positive impact for the sMCI class. Large values of ADAS 13 have a higher positive impact on the model for predicting the pMCI than ADNI_MEM. Small values of FDG have a greater positive impact for predicting pMCI than MOCA and ADAS 11. As a result, some features are not critical globally, but extreme values for specific cases have a greater impact in the model than the globally important features. Based on the knowledge of our domain experts, this is also medically intuitive, and increases the confidence of medical experts in the behavior of our system.

#### Explainability of individual cases

**Figure 4:**
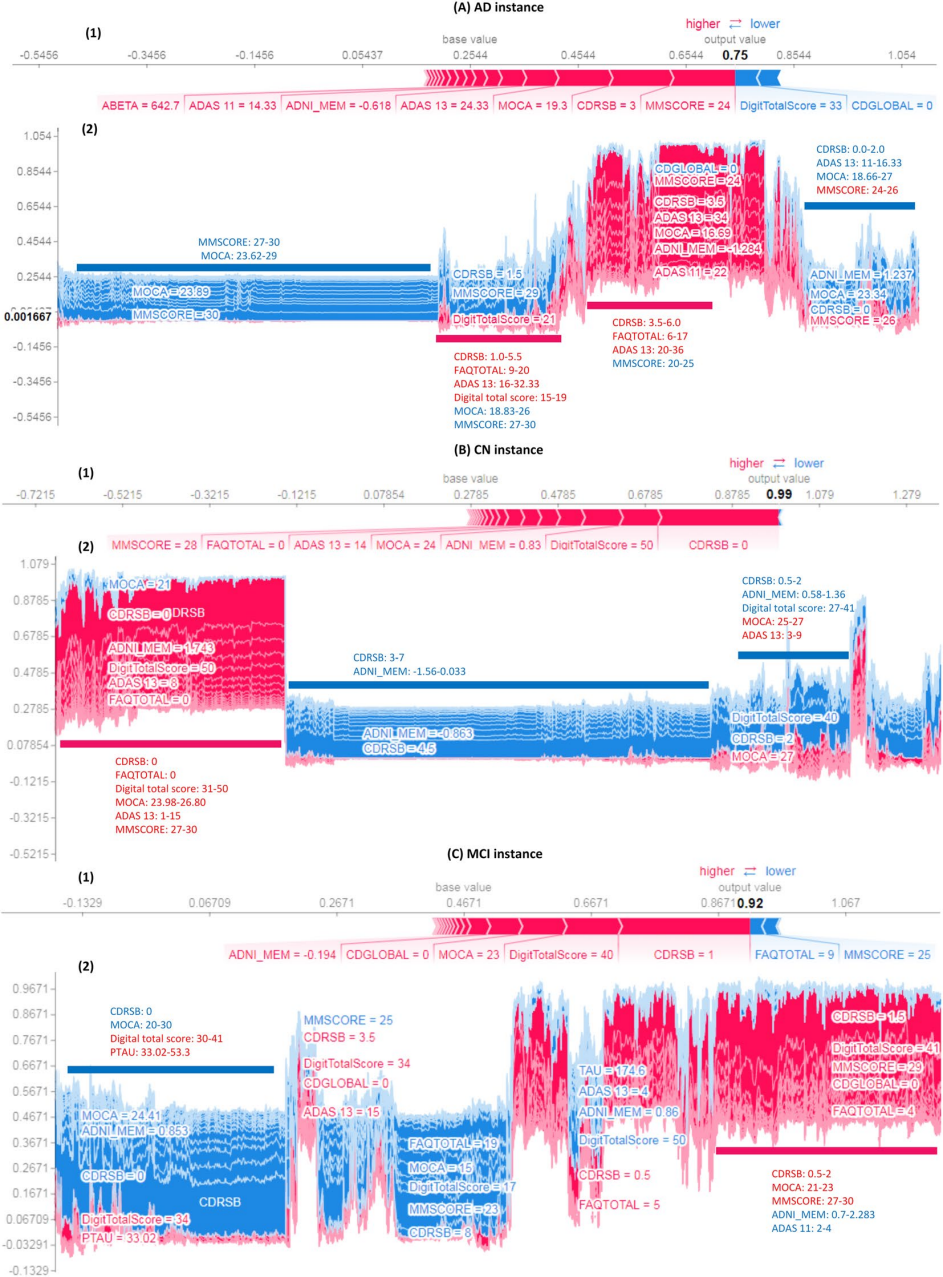
First layer example predictions for AD (**A**), CN (**B**), and MCI (**C**) and SHAP supervised clustering in model behavior for all cases in each class. Red indicates attributions that push the score higher, while blue indicates contributions that push the score lower. A few of the noticeable subgroups are annotated with the features that define them.

Figure 4 shows examples of prediction for each class in the First Layer, and Figure S2 in Supplementary file (part 2) shows another example from the Second Layer. In addition, the figure illustrates supervised clustering of all cases according to their similarities.

Each example is a vertical line, and SHAP values for all cases are ordered by similarity. We identify some critical values for each cluster. Figure 4 (A) (part 1) shows a case with a probability of 75% for being AD. It also shows the most significant feature values that have a positive impact for that class, such as MMSE = 24, CDRSB = 3.0, MOCA = 19.3, etc. This is consistent with ADNI data, where the average values of all AD subjects are MMSE = 23.235 ± 2.015, CDRSB = 4.3 ± 1.591, and MOCA = 17.553 ± 3.377. In addition, it shows the features that push the classification away from the AD class including DigitalTotalScore = 33, CDGLOBAL = 0, etc. The features with less impact such as TAU = 347.9, PTAU = 31.64, RAVLT immediate = 27, FAQ = 5, and Trial5Total = 7 are represented with short arrows. Figure 4 (A) (part 2) shows the behavior of the model on all the instances, and the role of each feature to support (red) or not support (blue) classification as AD. Different clusters are defined according to the values of critical features. We find that when MMSE is in the interval [27, 30] and MOCA is in [23.62, 29], this combination has the greatest role in preventing the model from selecting the AD class (blue cluster). On the other hand, when CDRSB is in the range [3.5, 6.0], FAQ is in [6–17], and ADAS 13 is in [20–36], the model will mostly classify cases as AD (red cluster).

Figure 4 (B) (part 1) does the same thing for the CN class. The model is 99% confident that the case is CN. Clearly defined clusters explain the model behavior in selecting the CN class. The most critical factors that push the decision towards CN class are CDRSB = 0 and DigitalTotalScore = 50. Figure 4 (B) (part 2) shows the overall logic in detecting CN subjects. We observe some critical values of some clusters from this figure. For example, if CDRSB = 0, FAQ = 0, DigitalTotalScore is in [31, 50], MMSE is in [27, 30], and ADAS 13 is in [1, 15], the patient is mostly classified as CN (red cluster). This means that if MMSE is combined with both CDRSB and FAQ at 0, it loses a lot of its impact on AD class prediction. According to the ADNI data and our experts’ knowledge, these decisions are medically intuitive because the average values of critical factors for CN subjects are CDRSB = 0.039 ± 0.141, FAQ = 0.194 ± 0.720, and DigitalTotalScore = 48.173 ± 7.481. Figure 4 (C) (part 1) shows the prediction of our model for an MCI case. In general, the characteristics of MCI cases are between those of CN and AD classifications. According to the model prediction, the low value of CDRSB (1 in this current case) has a high positive impact on predicting MCI cases. This subject has a negative ADNI_MEM value, which may have a significant impact on the system’s decision. By comparing the feature values of the three cases, we can say that a little change in CDRSB has a great impact on performance, and this is compatible with Fig. 2. Please note that the combination of different values of features could change the role of the related feature, as well as the final decision.

#### Explainability of the interaction between features

As shown in the middle of Figs. 3 and 4, many features (such as PTAU for AD class and ABETA for the CN and MCI classes), show a high degree of uncertainty. In addition, some features (such as Entorhinal and PTAU) seem to have less impact, because they are at the bottom of the list. However, these features may have a critical impact if they were combined with specific values of other features. To study the role of these types of features, we need to zoom in and study their behavior in combination with other features. Note that interaction analysis can be studied for other globally important features, as well, like CDRSB and FAQ.

Due to space restrictions, in Figure S3 of Supplementary File (part 2), we give a detailed example of the interaction impacts from one of these noisy features (e.g. PTAU) in the First Layer, and we study the impact of less globally critical feature (e.g., Entorhinal) in the Second Layer to highlight its role (see Fig. 2). As can be seen, the domain expert is able to interpret the internal behavior of the ML model and know exactly why it makes specific decisions. We notice that some features may be globally unimportant, but in some cases, they have extreme SHAP values, and that shows the real impact of these features. In addition, the real impact of a feature can be discovered by studying its interactions with other related features. Supplementary File (part 2) (Figure S4 to Figure S8) shows the SHAP interaction summaries for the most important features in both layers and for each class.

#### Explainability based on single explainers

In this section, we provide explanations of the RF model decisions from other explainers and based on other data types. Domain experts often consider these biomarkers to make accountable decisions. For example, the First Layer’s model does not consider MRI and PET data. Furthermore, the Second Layer’s model does not consider medical history. In addition, both models do not consider lab tests, vital signs, and physical examinations. However, all these features are considered by our explainers. It is worth noting that we are not interested in explaining the internal behavior of the RF model but providing physicians with post-hoc explanations of every decision. In the same way, how different physicians may figure out different explanations (in terms of different features) for a given output, our explainers yield complementary, consistent and reliable explanations.

Tables 9 and 10 summarize the quality (i.e. the performance-explainability trade-off) of the 22 explainers (11 DT and 11 FURIA) for each layer. Even if some of these explainers exhibit poor performance, they all exhibit complementary explainability because they depend on different features. In practice, these explainers provide physicians with plausible explanations in natural language. It is worth noting that given a specific data instance, only those explainers that point out at the same output class as the RF model are taken into account when generating explanations. Moreover, physicians are provided with explanations along with information about the reliability of each single explainer in terms of its balance between accuracy and explainability. At the end, the physician makes the final decision on which explainers to trust or to discard likewise she may ask for alternative opinions of different colleagues who are likely to have different experience and background. As expected, DT is clever for some modalities, while FURIA is better for others.

**Table 9:** The performance of the explainers on different modalities (First Layer).

Explainer	No. of features	Model	Exp. (%)	Precision	Recall	Accuracy	F1-score	Exp. measures
Cognitive scores based	12	DT	96 (91.43)	**0.913**	**0.912**	**0.912**	**0.912**	**NL = 29; S = 57**
		FURIA	97 (92.38)	0.910	0.909	0.909	0.909	NR = 17
Genetics based	5	DT	61 (58.10)	0.553	0.555	0.555	0.553	NL = 35; S = 69
		FURIA	63 (60.00)	**0.556**	**0.559**	**0.559**	**0.553**	**NR = 8**
Lab tests based	41	DT	39 (37.14)	0.408	0.407	0.407	0.408	NL = 155; S = 309
		FURIA	53 (50.48)	**0.469**	**0.494**	**0.494**	**0.460**	**NR = 6**
Medical history based	27	DT	83 (79.05)	0.495	0.496	0.496	0.494	NL = 173; S = 331
		FURIA	67 (63.81)	**0.521**	**0.519**	**0.519**	**0.517**	**NR = 10**
MRI based	8	DT	64 (60.95)	0.472	0.471	0.471	0.468	NL = 58; S = 115
		FURIA	51 (48.57)	**0.525**	**0.525**	**0.525**	**0.524**	**NR = 5**
Neurological exams based	12	DT	47 (44.76)	0.387	0.451	0.451	0.335	NL = 15; S = 29
		FURIA	47 (44.76)	**0.413**	**0.467**	**0.467**	**0.350**	**NR = 3**
Neuropsychological battery based	35	DT	87 (82.86)	0.733	0.732	0.732	0.732	NL = 85; S = 169
		FURIA	85 (80.95)	**0.771**	**0.771**	**0.771**	**0.770**	**NR = 22**
PET based	3	DT	60 (57.14)	0.614	0.612	0.612	0.610	NL = 22; S = 43
		FURIA	66 (62.86)	**0.621**	**0.618**	**0.618**	**0.616**	**NR = 6**
Physical exams based	10	DT	48 (45.71)	0.291	0.443	0.443	0.304	NL = 13; S = 25
		FURIA	49 (46.67)	**0.257**	**0.461**	**0.461**	**0.297**	**NR = 5**
Symptoms based	27	DT	49 (46.67)	**0.425**	**0.449**	**0.449**	**0.383**	**NL = 32; S = 63**
		FURIA	49 (46.67)	0.430	0.446	0.446	0.389	NR = 3
Vital signs based	8	DT	36 (34.29)	0.369	0.417	0.417	0.367	NL = 79; S = 157
		FURIA	45 (42.86)	**0.360**	**0.453**	**0.453**	**0.329**	**NR = 2**

Exp. measures, explainability measure; NL, number of leaves; S, size of the tree; NR, number of rules; Exp. (%), the number of explained cases (percentage of coverage); values in bold indicate the best performance.

**Table 10:** The performance of the explainers on different modalities (Second Layer).

Explainer	No. of features	Model	Exp. (%)	Precision	Recall	Accuracy	F1-score	Exp. measures
Cognitive scores based	12	DT	38 (77.55)	0.824	0.824	0.824	0.824	NL = 25; S = 49
		FURIA	38 (77.55)	**0.847**	**0.847**	**0.847**	**0.847**	**NR = 9**
Genetics based	5	DT	32 (65.31)	0.729	0.721	0.721	0.720	NL = 7; S = 13
		FURIA	33 (67.35)	**0.729**	**0.728**	**0.728**	**0.728**	**NR = 5**
Lab tests based	41	DT	28 (57.14)	0.528	0.526	0.526	0.526	NL = 58; S = 115
		FURIA	30 (61.22)	**0.574**	**0.574**	**0.574**	**0.574**	**NR = 20**
Medical history based	27	DT	29 (59.18)	0.590	0.590	0.590	0.589	NL = 51; S = 95
		FURIA	31 (63.27)	**0.643**	**0.643**	**0.643**	**0.643**	**NR = 3**
MRI based	8	DT	30 (61.22)	**0.732**	**0.721**	**0.721**	**0.720**	**NL = 12; S = 23**
		FURIA	33 (67.35)	0.687	0.686	0.686	0.687	NR = 6
Neurological exams based	12	DT	23 (46.94)	**0.492**	**0.515**	**0.515**	**0.429**	**NL = 9; S = 17**
		FURIA	24 (48.98)	0.457	0.497	0.497	0.417	NR = 3
Neuropsychological battery based	35	DT	31 (63.27)	0.693	0.693	0.693	0.693	NL = 52; S = 103
		FURIA	35 (71.43)	**0.764**	**0.762**	**0.762**	**0.762**	**NR = 11**
PET based	3	DT	31 (63.27)	0.680	0.677	0.677	0.677	NL = 8; S = 15
		FURIA	34 (69.39)	**0.710**	**0.709**	**0.709**	**0.709**	**NR = 4**
Physical exams based	10	DT	31 (63.27)	**0.552**	**0.542**	**0.542**	**0.537**	**NL = 14; S = 27**
		FURIA	29 (59.18)	0.534	0.529	0.529	0.526	NR = 5
Symptoms based	27	DT	27 (55.10)	0.531	0.524	0.524	0.520	NL = 20; S = 39
		FURIA	32 (65.31)	**0.556**	**0.547**	**0.547**	**0.542**	**NR = 4**
Vital signs based	8	DT	24 (48.98)	0.519	0.526	0.526	0.461	NL = 4; S = 7
		FURIA	31 (63.27)	**0.523**	**0.526**	**0.526**	**0.519**	**NR = 3**

Exp. measures, explainability measure; NL, number of leaves; S, size of the tree; NR, number of rules; Exp. (%), the number of explained cases (percentage of coverage); values in bold indicate the best performance.

We analyzed each instance in the test dataset of both layers and recorded how many explainers could predict the same class as their corresponding oracle (i.e. the RF model). The test set in the First Layer was made up of 105 instances. On average, 58.1% of the instances were managed by each single explainer. Regarding the number of explainers that act for each single instance, we found there were 13 (the median value) explainers considered; being 3 the worst case and 22 the best case. Being DT (vital signs-based) the least used explainer (34.4%) and FURIA (cognitive scores based) the most used explainer (92.4%). The test set in the Second Layer was made up of 49 instances. On average, 63% of the instances were managed by each single explainer. Being DT (neurological exams-based) the least used explainer (47%) and both DT and FURIA (cognitive scores-based) the most used explainers (78%). Regarding the number of explainers which act for each single instance, we observed that 14 (the median value) explainers are considered; being 7 the worst case and 20 the best case. All in all, we can conclude that even in the worst cases, we are ready to supply physicians with more than one single explanation. Moreover, explanations are normally rich, thanks to the fact that they involve several modalities. This fact was especially well appreciated by the physicians who collaborated in our study.

#### Case studies for FURIA and DT explainers

The supplementary file (part 3) lists a group of AD cases to tests the system explainability. The supplementary file (part 2) shows the expressiveness of the generated explanations for three illustrative case studies (see Supplementary File [part 2], Table T3 to T7). We tested the following: (1) the ability of explainers to generate supplementary explanations, (2) their consistency with the generated explanations from SHAP, and (3) the quality of the generated natural language explanations. In case study 1, we can see that generated explanations add many values to the interpretability and confidence of the decisions made. First, the explainers reinforce the explanations from SHAP. Second, they increase the confidence physicians have about the decision made. In case study 2, physicians can investigate all the information to understand why the system makes a specific decision. We note perfect matches among SHAP and explainers’ outputs. In case study 3, we observe how explanations related to sMCI and pMCI are somehow in contrast (and in accordance with) physicians’ intuition and background.

#### Model strengths and limitations

The proposed model is designed to comprehensively integrate high-fidelity Alzheimer’s data to predict AD and detect its possible progression within three years from baseline. We demonstrated the high predictive powers of the proposed models. The First Layer model achieves the best results by combining the NB, CS, and Genetics modalities. These modalities achieved the best cross-validations results. On the other hand, the Second Layer model shows the highest results based on CS, NB, PET, MRI, and Genetics. Both CS and NB have important roles in improving the performance of our model. Similar observations have been reported in the literature^20^. Note that not all biomarkers of these modalities are used in the training process, but only the features selected by the RFE technique. Using black-box models in the medical domain is very dangerous and not acceptable. Our model achieves superior performance, compared to other ML models; in addition, it combines high-accuracy, complex models (i.e. ensemble RF) with interpretable explanations. This combination allows physicians to receive the best possible predictions, and at the same time, gain insight into why those predictions were made. These actionable decisions increase the confidence and trust in the model’s behavior, help to debug the model, and can work as an educational tool for inexperienced physicians. Note that we used the word “*confidence*” to indicate that the model provided its results with small variances. In contrast, we used the word “*trust*” to indicate that our model provided interpretable and explainable results which improved the domain expert’s trust in the model’s decision. Moreover, when the model provided a result with high confidence, it then enhanced the domain expert’s level of trust. Consequently, in our study, more confidence resulted in more trust, in addition to the trust gained from explainability. The quantification of trust for deep learning models has been discussed recently^59^. Taking this quantification process into account would be an insightful investigation.

Training general practitioners, based on educational interventions, to recognize and manage AD has no significant impact on clinical practice^60^. A CDSS can provide another solution, but current systems are mostly based on a single modality^52,53^, make use of binary models (e.g., CADi2)^61^, or are not explainable^8–15^. As a result, current systems are rarely used routinely in AD management. We believe that a CDSS based on our comprehensive, accurate, and explainable model could make a difference in practice. We provide explanations from different perspectives including CS, NB, MRI, PET, Genetics, medical history, etc. In addition, we provide detailed explanations based on feature contributions. We believe that these explanations provide supplementary knowledge for physicians to fully understand the rationale behind the decisions taken. *To the best of our knowledge, this is the first study that provides such a comprehensive model and with such explainability features.*

Our model has a couple of limitations worth noting. *First*, we only considered the baseline data for making decisions. Because AD is a chronic disease, a time-series data analysis would be of critical value^62,63^. A future attempt will study the role of longitudinal data to enhance the model’s accuracy and explainability. We could consider some DL techniques, which are clever at handling time-series data, such as long short-term memory, in such a future study. *Second*, the ADNI collects data about the roles of a patient’s medication history and comorbidities on AD progression. No such research has been done previously to study these data. Another future enhancement could be the integration into the prediction ML model of semantic intelligence from ontologies. We will consider semantics from the standard ontologies (e.g. RxNorm, Systematized Nomenclature of Medicine-Clinical Terms [SNOMED CT], etc.) to encode these data and to infer hidden knowledge about the relationships between drugs, diseases, and Alzheimer’s. *Third*, the network science approaches have been used to characterizing the brain activities for AD patients to extract interconnectivity patterns of brain regions based on neuroimaging techniques^64–67^. Although these studies provided additional insights into AD pathophysiology, they come with several limitations. For example, Chen et al.,^64^ used a small cohort of 55 subjects for classifying subjects as AD vs. MCI vs. AD using the large-scale network analysis approach. These data have been collected at baseline visit only, and no longitudinal study has been performed. However, cross-sectional studies cannot dynamically observe changes in network patterns with disease progression. Furthermore, postmortem studies are required as the reference standard when validating the large-scale network methods. In addition, the study used simple linear regression to measure the relationship between changes in network connectivity strengths and behavioral scores. Wang et al.^65^ utilized a small dataset of 89 subjects to evaluate the impaired network functional connectivity with AD progression. Even though the whole brain network is complex, varied, and interrelated, this study was based on only five networks which put limitations placed on its results. Thus, the entire brain network analysis with finely defined regions is important. Also, this study is based on baseline data only. Besides, longitudinal data of multiple modalities such as functional and structural MRI, PET, genetic genotype, etc. should be fused to follow individuals to differentiate all the severity levels. In future studies, one might explore these network science approaches and integrate them with advanced XAI and deep learning techniques. In this context, we can study the roles of time series data to improve the current literature. Moreover, the role of data fusion of different modalities might be explored using different ML and DL algorithms. *Finally*, a web based CDSS system based on a user-friendly interface can provide medically intuitive aids for both medical experts and general practitioners. Work is currently in progress to develop such a system, which will be extended to work as a pluggable component of the electronic health record ecosystem. This design facilitates data entry by the physician, online training of the models, and automatic updates on patient status.

## Material and methods

### ADNI study

Data used in this work was collected from the ADNI database (*adni.loni.usc.edu*). Subjects have been enrolled from over 57 sites across the U.S. and Canada. The study was conducted according to the Good Clinical Practice guidelines, the Declaration of Helsinki, US 21 CFR part 50 —Protection of Human Subjects—and part 56—Institutional Review Boards. Subjects were willing and able to undergo test procedures, including neuroimaging and follow-up, and written informed consent was obtained from participants. All data are publicly available, at http://adni.loni.usc.edu/.

In all, 1048 subjects (54.5% male) participated in the study and were categorized into four groups based on the individual clinical diagnosis at baseline and future visits, as follows: (a) cognitively normal (CN): 294 subjects (28.1%) diagnosed as CN at baseline who remained CN at the time this manuscript is prepared. (b) sMCI: 254 subjects (24.2%) diagnosed as MCI at all-time points. (c) pMCI: 232 subjects (22.1%) evaluated as MCI at baseline visit who had progressed to AD within three years. (d) AD: 268 subjects (25.6%) who had a clinical diagnosis of AD for all visits. Subjects showing improvement in their clinical diagnosis during follow up (i.e., those clinically diagnosed as MCI but reverting to CN, or those clinically diagnosed as AD but reverting to MCI or CN) were excluded from the study because of the potential uncertainty of clinical misdiagnosis, considering that AD is considered irreversible form of dementia. In addition, cases that had a direct conversion from CN to AD were also removed. Patients taking part in this study are anonymized and the actual list of patient IDs in our study can be found in Supplementary File (part 1). The data used in this research are from the baseline visits only, no longitudinal data were considered.

### Study cohorts

Eligible participant patients were from 55 to 91 years old, fluent in English or Spanish, and had at least six years of education. Participants were categorized into three groups: CN, MCI (sMCI + pMCI), or AD. CN individuals were free of memory complaints, had a mini-mental state examination (MMSE) score of 24 to 30, and an average clinical dementia rating sum of boxes score (CDR-SB) of 0.04. MCI individuals had MMSE scores of 23 to 30, and an average CDR-SB of 1.582. MMSE and CDR-SB scores for MCI subjects were considerably different from CN subjects (*P* < *0.0001*). The ages of MCI subjects were significantly different from AD and CN subjects (*P* < *0.005*). The years of education for MCI subjects were significantly different from CN subjects (*P* < *0.01*). AD patients fulfill diagnostic criteria for probable AD as set by the National Institute of Neurological and Communicative Disorders and Stroke of the United States and the Alzheimer’s Disease and Related Disorders Association^68^, with MMSE scores of 19 to 27 and an average CDR-SB of 4.347. MMSE and CDR-SB scores of AD subjects were significantly different from CN and MCI subjects (*p* < *0.0001*). The ages of AD subjects were significantly different from CN subjects (*P* < *0.05*), and the education years of AD subjects were significantly different from CN subjects (*P* < *0.0001*) and MCI subjects (*P* < *0.01*). Available ADNI subjects (n = 1048) with both a T1-weighted MRI scan and a PET–fluorodeoxyglucose (PET-FDG) image upon preparation of this manuscript were used in this study. For the PET data, we collected only three PET-FDG features from Banner Alzheimer’s Institute (BAI)-PET Naval Medical Research Center (NMRC) summaries and University of California, Berkeley, FDG analysis^69^. The MRI features used in our experiments are based on the imaging data from the ADNI database processed by a team from UCSF, who performed cortical reconstruction and volumetric segmentations with the FreeSurfer version 6.0 image analysis suite (https://surfer.nmr.mgh.harvard.edu/) according to the atlas generated by Desikan et al.^70^.

The FreeSurfer software version 6.0 (https://surfer.nmr.mgh.harvard.edu/) was employed to automatically label cortical and subcortical tissue classes for the structural MRI scan of each subject, and to extract thickness measures of cortical regions of interest and cortical and subcortical volume measures. Based on the 312 features collected from each MRI image, we calculated seven features including ventricles, middle temporal gyrus [midTemp], fusiform, entorhinal, hippocampus, and whole brain volume. The equations used to calculate these features can be found in Supplementary File (part 2). Details of the analysis procedure are available at ADNI (http://adni.loni.usc.edu/methods/mri-tool/mri-analysis/). Detailed descriptions of the ADNI subjects, image acquisition protocol procedures, and post-acquisition preprocessing procedures can be found at ADNI (http://www.adni-info.org/). Demographic and clinical information of the subjects is shown in Table 11. In this study, we utilized multiple modalities that include the followings: (i) Cognitive scores, e.g. 12 features of the Alzheimer’s diseases assessment scale–cognitive subscale (ADAS-Cog) 11, ADAS-Cog 13, global CDR (CDGLOBAL), CDRSB, functional assessment questionnaire (FAQ), geriatric depression scale (GDT), MMSE, Montreal cognitive assessment (MoCA), and the neuropsychiatric inventory questionnaire score (NPISCORE). (ii) PET features, i.e. FDG, hypometabolic convergence index (HCI), and statistical region of interest [SROI]). (iii) Neuropsychological battery, i.e. 35 features of the Rey auditory verbal learning test (RAVLT), CLOCK, COPY, and AVTOT total scores and sub-scores. (iv) Neuropathology vital signs, i.e. seven features including body mass index (BMI), weight, blood pressure, etc. (v) Cerebrospinal fluid (CSF) biomarkers, i.e. TAU, phosphorylated TAU—PTAU, and amyloid-β peptide of 42 amino acids- Aβ_1–42_. (vi) Demographics, i.e. gender, age, number of years of education, marital status, and ethnic and racial categories. (vii) Medical history, i.e. 22 binary features to check the patient and parents histories, including smoking, allergies, malignancy, gastrointestinal problems, etc. (viii) Symptoms, i.e. 27 binary features asking about diarrhea, dizziness, falls, etc. (ix) Lab tests, i.e. 41 blood lab tests, including vitamin B12, monocytes, platelets, etc. (x) Physical examinations, i.e. 10 feature asking about problems in the head, neck, skin, chest, etc. (xi) Neurological exams, i.e. 12 binary features from the cerebellar exam, gait, motor strength, sensory capabilities, etc. (xii) MRI volumetric features, i.e. volumes of ventricles, MidTemp, fusiform, entorhinal cortex, hippocampus, total intracranial volume (ICV), and whole brain. (xiii) Genetics, i.e. APOE4. *To the best of our knowledge, there are no studies in the literature, which study the role of all of these biomarkers.* More details about these features can be found in Supplementary File (part 2).

**Table 11:** Descriptive statistics from the dataset used.

	CN (n = 294)	sMCI (n = 254)	pMCI (n = 232)	AD (n = 268)	Combined (n = 1048)
Gender (M/F)	140 ± 154	144/110	136/96	151 ± 117	571/477
Age (years)	74.120 ± 5.890	72.202 ± 07.553	73.771 ± 7.0840	75.241 ± 7.610	73.864 ± 07.107
Education (years)	16.405 ± 2.733	15.9530 ± 2.867	15.784 ± 2.7830	15.175 ± 2.923	15.844 ± 02.858
FAQ	0.1940 ± 0.720	01.539 ± 02.817	5.7110 ± 4.8680	13.146 ± 6.814	05.053 ± 06.754
MMSE	29.085 ± 1.143	27.941 ± 01.722	26.7590 ± 1.736	23.235 ± 2.015	26.797 ± 2.7960
MOCA	25.569 ± 1.866	23.493 ± 02.452	20.947 ± 01.908	17.553 ± 3.377	21.993 ± 3.9450
FDG	6.5690 ± 0.477	06.3820 ± 0.599	05.800 ± 00.462	5.4060 ± 0.614	6.0560 ± 0.7180
APOE4	0.2520 ± 0.472	0.4610 ± 0.6380	0.8660 ± 00.686	0.8880 ± 0.710	0.6010 ± 0.6850
CSF PTAU pg/mL	19.423 ± 6.820	25.640 ± 11.703	35.2240 ± 13.20	35.717 ± 13.11	28.5940 ± 13.29
CSF TAU pg/mL	215.07 ± 67.28	270.861 ± 106.4	352.86 ± 116.27	361.2 ± 121.41	296.46 ± 120.58
ADAS-Cog 11	05.617 ± 2.784	8.6260 ± 3.5200	13.412 ± 4.3850	19.318 ± 6.569	11.576 ± 06.970
ADAS-Cog 13	08.600 ± 4.108	13.791 ± 05.303	21.580 ± 05.841	29.706 ± 7.835	18.129 ± 10.085
RAVLT immediate	045.595 ± 9.64	37.705 ± 10.308	27.444 ± 06.510	22.466 ± 7.069	33.750 ± 12.585
RAVLT learn	06.139 ± 2.143	04.799 ± 02.403	02.853 ± 02.219	01.799 ± 1.810	3.9770 ± 02.752
RAVLT forgetting	03.582 ± 2.810	04.343 ± 02.497	05.039 ± 02.193	04.381 ± 1.783	4.2930 ± 02.420
RAVLT % forget	32.612 ± 27.53	50.000 ± 30.027	78.188 ± 27.892	88.562 ± 21.22	61.223 ± 35.098
CDR-SB	0.0390 ± 0.141	1.1970 ± 0.6390	02.004 ± 0.9980	04.347 ± 1.591	01.856 ± 01.896
GDTOTAL	0.7890 ± 1.056	1.7090 ± 01.462	01.668 ± 01.423	01.634 ± 1.454	01.423 ± 01.404
HCI	8.9500 ± 3.330	11.066 ± 04.080	15.560 ± 04.770	21.158 ± 7.384	14.048 ± 06.996
Hippo. vol. (cm^3^) (/1000)	7.4520 ± 0.920	07.106 ± 01.074	06.083 ± 01.038	05.713 ± 0.995	06.621 ± 01.240

AD, Alzheimer’s disease; MCI, mild cognitive impairment; pMCI-sMCI, progressive MCI – stable MCI; CN, cognitive normal; CDR, clinical dementia rating; ADAS-Cog, Alzheimer’s Disease Assessment Scale–Cognitive Subscale test; RAVLT, Rey Auditory Verbal Learning Test; FAQ, Functional Assessment Questionnaire; MMSE, Mini–Mental State Examination; FDG, sum of mean glucose metabolism uptake in regions of angular, temporal, and posterior cingulate; TAU, CSF level of TAU; Aβ42, CSF level of amyloid β1–42 peptide; HCI, hypometabolic convergence index; AV45, Average AV45 SUVR of frontal, anterior cingulate, precuneus, and parietal cortex relative to the cerebellum; Hippo, Hippocampus; GDTOTAL, Geriatric Depression Scale.

*Data are mean ± standard deviation.

### Feature selection and modeling approach

**Figure 5:**
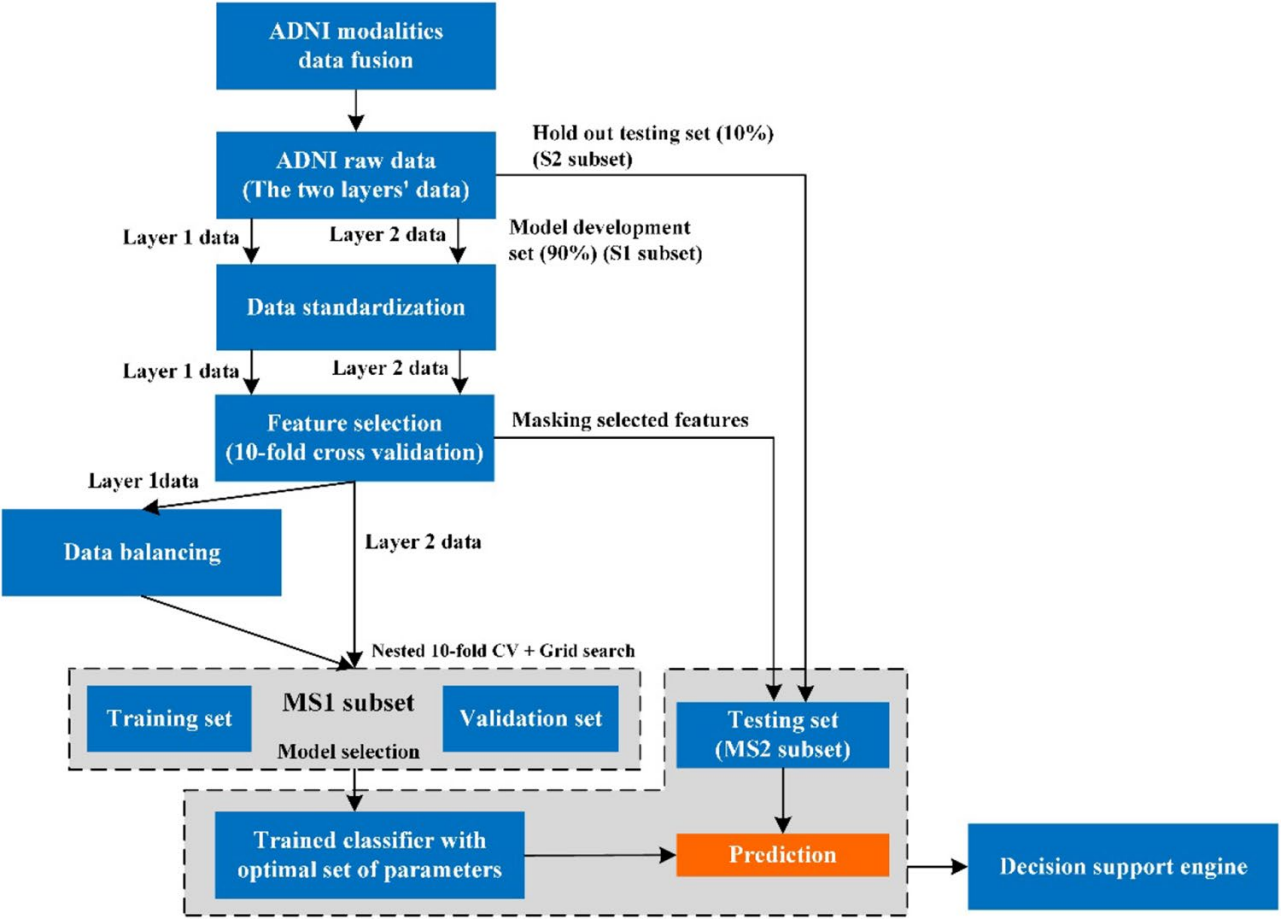
Development process for the oracle model in each layer.

The proposed model has two main layers. Each layer has an oracle classifier based on RF and a set of 22 explainers. The oracle is trained to be as accurate as possible based on the fused dataset. The First Layer’s oracle classifies the patient as CN, MCI, or AD based on the whole dataset. The Second Layer’s oracle concentrates further on the MCI cases, filtered from the previous layer, to predict their probable progression to AD within three years from baseline. As such, the Second Layer classifies the MCI cases into sMCI and pMCI cases. The development process of the proposed oracles has several major steps, as presented in Fig. 5. These steps are applied in the same order for both layers separately. *First*, after fusing the raw data modalities, for each layer, the full dataset is stratified and randomly divided into a model development set [$S1$] (90%) and a testing set [$S2$] (10%) that is utilized to evaluate and compare the generality and explainability of models. This split prevents the mixing of model-selection and performance estimation, which supports the estimations of unbiased generalization performance from the models. *Second*, a feature standardization step is assimilated on numerical features to normalize them in the same way, which is done by standardizing the random variables with zero mean and unitary standard deviation. Note that categorical features are excluded from the normalization process.

*Third*, for enhanced generalization performance of the models, the $S1$ set is used to implement a feature selection process to identify the most relevant features. *Fourth*, most ML approaches tend to generate biased models when handling imbalanced datasets. Our Second Layer’s dataset is balanced (52.3% sMCI and 47.7% pMCI). However, the First Layer’s dataset is imbalanced (28.05% CN, 46.37% MCI, and 25.58% AD). Therefore, the synthetic minority oversampling technique (SMOTE) is used to handle the class imbalance in the $S1$ set of the First Layer by resampling the original data and creating synthetic instances^71^. *Fifth*, to guarantee unbiased tuning of model hyperparameters, and because our datasets are relatively small, the model selection and validation process (i.e. hyperparameter optimization) is carried out based on the grid search and *nested k*-fold stratified cross-validation (CV) where *k* = *10*^72^. The entire process has two loops: an inner loop for hyperparameter tuning, and an outer loop for evaluation of the model with selected parameters on unseen data^73^. Model selection without nested CV uses the same data from parameter tuning and model evaluation, where information may leak into the model and overfit the data. The leave-one-out cross-validation (LOOCV), i.e. k-fold CV where *k* = *n*^72^, assures small bias but large variance^74^. The tenfold CV provides the best trade-off between bias and variance^75^. Keeping the $S2$ set untouched helps us to verify that the generalization performance of the selected model thanks to tenfold CV is preserved even with unseen data. In each layer, we develop an RF classification model based on the selected features.

RF classifiers are used because they are accurate, and it is possible to get the feature contributions for the whole model (a global explanation) and calculate feature contributions for each specific instance (a local explanation). Although SVM and DL have a huge capability to fit complex nonlinear models to the data and achieve high performance, the resultant models are opaque what makes hard to explain their decisions^18^. We therefore selected RF as the oracle to classify patients in our two-layer model.

After building the RF oracle classifiers, we implement two interpretable classifiers (DT and FRBS) for each of the 11 modalities in each layer. The resulting 22 classifiers play the role of explainers to interpret the oracle decisions at each layer. Thus, we have 11 classifiers as a DT, and 11 classifiers as an FRBS. The FRBS deals naturally with imprecision and uncertainty^36^. Moreover, an FRBS plays an important role in the quest for XAI^76^. More precisely, we selected the Fuzzy Unordered Rule Induction Algorithm (FURIA) [51] from among all algorithms available for building an FRBS. FURIA is recognized as one of the most accurate fuzzy classifiers. In addition, FURIA usually yields a compact set of fuzzy IF–THEN rules. FURIA is based on the Repeated Incremental Pruning to Produce Error Reduction (RIPPER) algorithm^77^. FURIA translates RIPPER rule antecedents into trapezoidal fuzzy sets. These antecedents are related by FURIA weighed rules, which do not necessarily include an antecedent for all the input attributes and can have more than one antecedent for the same attribute. Each FURIA rule is associated with a certainty factor, i.e. a rule weight that FURIA computes regarding the relevance of the rule in accordance with the training data. Given a specific data instance, the min–max fuzzy inference mechanism is applied, and the winning rule, i.e. the one with maximum firing degree, determines the output class. If no rules are fired for a given data instance, then FURIA applies the so-called rule-stretching mechanism, which looks for slight modifications in the rule base with the aim of finding a new rule on-the-fly that is able to manage the given instance. Unfortunately, FURIA rules lack linguistic meaning because they have local semantics, i.e. the most suitable fuzzy sets are defined independently for each rule. This fact may jeopardize the interpretability of FURIA rules.

With the aim of paving the way from interpretable to explainable classifiers, we use ExpliClas^78^. This is a web service ready to provide users with multimodal (textual + graphical) explanations related to the DT and FURIA. As a matter of fact, ExpliClas creates a linguistic layer on top of the DT and FURIA. First, global semantics (whether we consider the DT or FURIA) is set up beforehand. By default, three linguistic terms (e.g., low, medium, high) are defined for each attribute. Next, domain experts (if available) can add/remove/refine the given linguistic terms to assure they are meaningful. Then, given a specific data instance, the actual classification carried out by the DT or FURIA is automatically interpreted by ExpliClas with regard to the linguistic terms previously defined. In practice, both the activated branch of the DT and the winner rule of FURIA are translated into sequences of meaningful words (i.e., each numerical interval in the DT or fuzzy set in FURIA is verbalized by the closest linguistic term in ExpliClas). As a result, users are provided with an explanation in natural language of the output class in terms of the involved attributes. It is worth noting that we substituted the default linguistic terms in ExpliClas by meaningful linguistic terms in agreement with a physician in this study.

**Figure 6:**
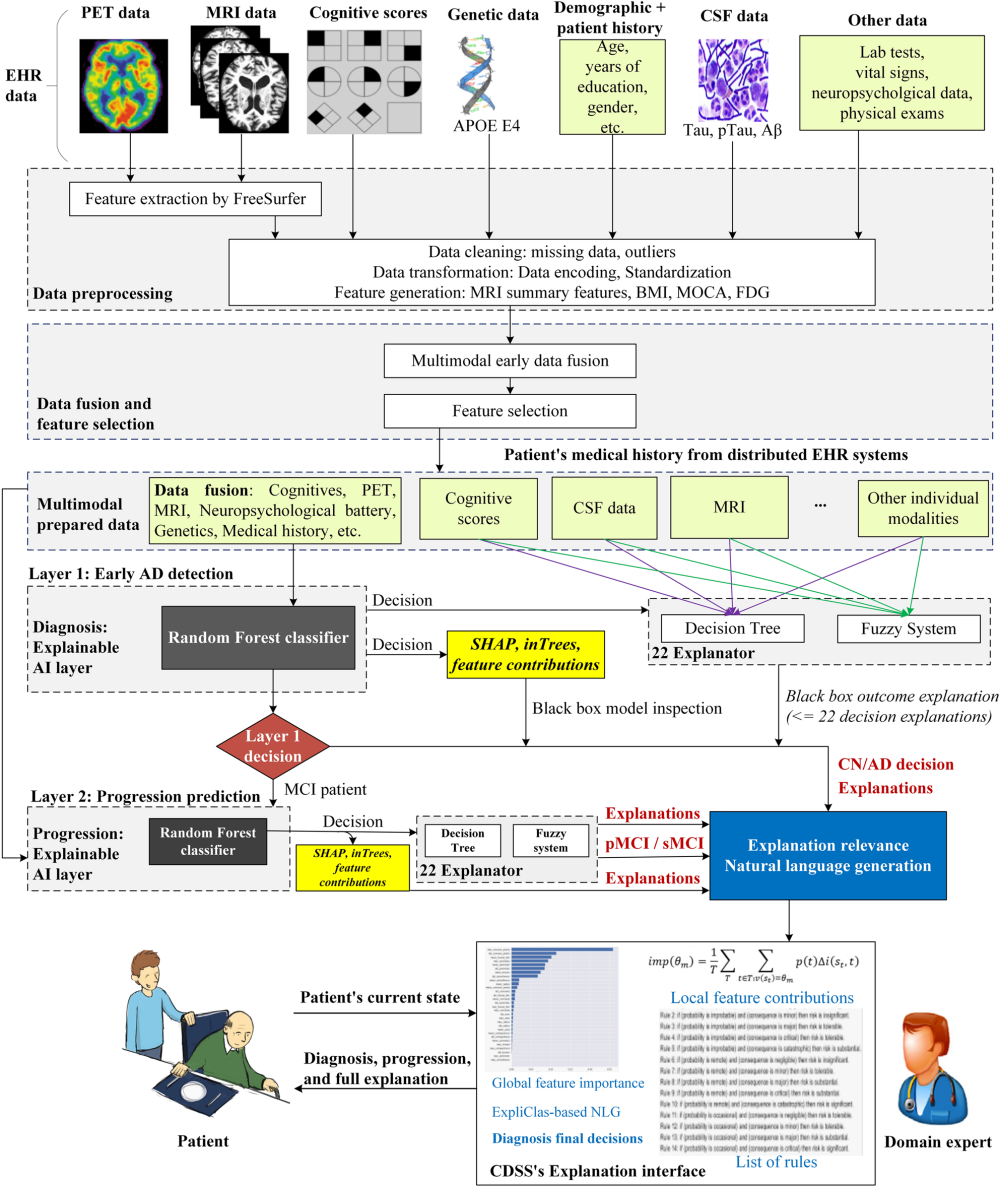
The proposed XAI framework. A variety of data modalities are used to build the predictive model. In addition, a variety of explanations are built for the entire RF behavior and for each prediction. The FreeSurfer version 6.0 is used (https://surfer.nmr.mgh.harvard.edu/).

Figure 6 shows a detailed description of our proposed XAI framework. The first step is preprocessing, which is used to prepare and improve the quality of the datasets. This step has the following four sub-processes.

**Figure 7:**
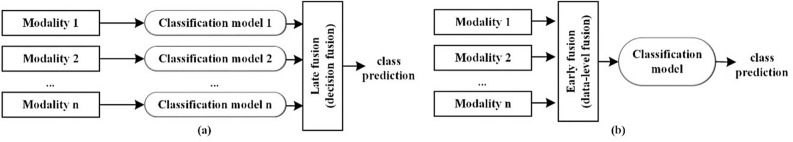
Multimodal fusion strategies: (**a**) late fusion, (**b**) early fusion.

- *Preparing biological modalities:* For the biological MRI modality, we used ready-made extracted and pre-processed features (http://adni.loni.usc.edu/), done by ADNI. We then used these detailed features to create a list of seven volumetric summary features for the most critical brain regions of interest, including the hippocampus, ventricles, entorhinal, fusiform gyrus, MidTemp, whole brain, and ICV. For biological PET modality, we collected only three FDG-PET features from BAI-PET NMRC summaries and UC Berkeley-FDG analysis^69^. For instance, to measure FDG, mean levels of glucose metabolisms are first recorded at different regions of interest. The five most common regions are left and right angular gyri, posterior cingulate cortex, and left and right inferior temporal gyri. Then, the summation of the mean glucose metabolisms is considered FDG^79^. Other PET measures include the HCI to characterize in a single summary metric the extent to which both the magnitude and spatial extent of cerebral glucose hypometabolism in a person’s FDG-PET image corresponds to that in patients with probable AD dementia^80^. Our prepared PET and MRI features are based on their popularity in studies from the literature, their availability, and their level of accuracy in our current medical problem (see Supplementary File [part 2] for further details).
- *Multimodal fusion:* The AD environment is multimodal in nature, where multiple feature sets are combined. This is called multimodal fusion, where each modality has supplementary information to support the final decision. In this context, two simple strategies are followed: late fusion and early fusion. In late fusion (i.e., decision-level fusion), a different model is trained independently for each modality, and the individual outcomes are merged into a final common decision, as seen in Fig. 7a. In the early fusion strategy (i.e., feature-level fusion), raw features from the individual modalities are integrated to create a common feature vector. The common feature vector is then used to train a classifier as the final prediction model, as seen in Fig. 7b. Each strategy has its own advantages and disadvantages. However, late fusion is based mainly on computing weights associated to which classifiers, which is not an easy process to learn and to explain. Therefore, in this study, we apply the early fusion strategy.
- *Data standardization:* After data splitting, each type of participating data may have a different order of magnitude. These raw data cannot be used directly to train the RF model. To ensure that every feature has the same level of importance, data were standardized using the z-score method (see Eq. 1). The standardized data is therefore normally distributed with mean and standard deviation of 0 and 1, respectively.
  1 $$z_{j} = \frac{{x_{j} - \mu_{j} }}{{\sigma_{j} }}$$
  where ${x}_{j}$ is the old value of feature $j$, ${z}_{j}$ is the normalized value, ${\mu }_{j}$ is the feature’s mean, and ${\sigma }_{j}$ is the feature’s standard deviation. As a side effect, this method removes outliers.
- *Handling missing values:* For handling missing values, we first removed any feature with more than 30% of the values missing. Then, we use the k-nearest neighbors (KNN) algorithm to impute missing values, where missing values are replaced using information from neighbor subjects that have the same class. After finding $k$ neighbors, the imputation value is computed by averaging the values of those neighbors. In our study, the mixed Euclidean distance (MED) was used, and *k* was set to 10 empirically via experiments (for numerical values, the Euclidean distance was used; for categorical values, a distance of 0 was taken if both values were the same, otherwise the distance was set to 1). Please note that the data standardization process has been done before the missing values handling.
  2 $$MED\left( {x,y} \right) = \sqrt {\mathop \sum \limits_{i = 1}^{N} d_{i} \left( {x_{i} ,y_{i} } \right)^{2} }$$
  where $$d_{i} \left( {x_{i} ,y_{i} } \right) = \left\{ {\begin{array}{*{20}l} {overlab\left( {x_{i} ,y_{i} } \right)} \hfill & {if\;i\;is\;categorical} \hfill \\ {diff\left( {x_{i} ,y_{i} } \right)} \hfill & {if\;i\;is\;numerical} \hfill \\ \end{array} } \right.$$, $$overlab\left( {x_{i} ,y_{i} } \right) = \left\{ {\begin{array}{*{20}l} 0 \hfill & {if\;x_{i} = y_{i} } \hfill \\ 1 \hfill & {if\;x_{i} \ne y_{i} } \hfill \\ \end{array} } \right.$$, and $$diff\left({x}_{i},{y}_{i}\right)=\frac{{x}_{i}-{y}_{i}}{{max}_{i}-{min}_{i}}$$

For the automatic feature selection, we used wrapper methods, which obtain subsets of features, and offer better performance than filter methods^20^. The commonly used classifiers in wrapper are naïve Bayes^81^, SVM^82^, RF^83^, and AdaBoost^84^. Along with greedy search algorithms, these methods find the optimal set of features. It is worth noting that the well-known principal component analysis (PCA) technique cannot be used in our experiments because we need to preserve meaningful medical features, and PCA produces synthetic features that are hard to interpret as a combination of the principal components.

Recursive feature elimination (RFE) is famous in the medical domain owing to its efficiency in reducing computational burden^85^. It maximizes its predictor performance through backward feature elimination as well as its ranking criterion. The literature asserts that RF-RFE outperforms SVM-RFE in finding the best subsets of features, and does not need any parameter regulation to offer reasonable outcomes^86^. We applied RFE with the stratified tenfold CV related to the $S1$ dataset. To prevent the bias introduced by randomly partitioning a dataset in CV, the tenfold CV procedure was repeated five times with different data partitions. To evaluate the robustness of the RF-RFE process in selecting the optimal set of features, we utilized the RFE method with RF, SVM, and gradient boosting (GB) classifiers. The initial fused feature set had 188 features combined from 11 different modalities, including MRI, genetics, and symptoms.

The two RF models are used as the oracle to make the final decisions. Of course, final decisions are made by physicians in light of the provided information (i.e., both oracle decisions, along with related explanations). The 11 modalities are used separately to build classifiers by using two interpretable ML models, i.e. DT and FURIA. In each layer, the resulting 22 interpretable models are used to support the oracle model by providing interpretations of its decisions. The supplementary explanations extracted from different modalities with different classification algorithms are expected to enhance the medical expert’s confidence in the oracle decisions. As a result, it supports the applicability of the resulting system in real medical environments. It is worth noting that we are not interested in explaining the internal behavior of the oracle but providing physicians with post-hoc explanations of the decision output. Our approach is inspired in the way how different experts who look at the same patient may figure out different explanations for a given output in terms of different features (i.e., in accordance with their own knowledge and background). Similarly, our explainers provide physicians with complementary explanations, all of them consistent and reliable.

### Random forest for classification

RF is an ensemble classifier formed by a family of $T$ decision trees,$h\left({n}_{1}|{\theta }_{1}\right),\dots , h\left({n}_{T}|{\theta }_{T}\right)$, where ${\theta }_{i}=({\theta }_{i1},{\theta }_{i2}{,\dots ,\theta }_{ip})$ is a list of $p$ features for DT $i$, and ${n}_{i}$ represents the training instances. Each DT leads to a classifier. Specifically, given data $D={\{{(\theta }_{i},{y}_{i})\}}_{i=1}^{N}$, we train a family of classifiers,${h}_{T}$. The predictions of all individual trees are combined by using the majority-voting mechanism. A node is partitioned using the best possible binary split. In our case, information gain is used to define the split point at each node, where $G\left(S,A\right)=E\left(S\right)-\sum_{v\in values\left(A\right)}\frac{\left|{S}_{v}\right|}{\left|S\right|}E\left({S}_{V}\right)$, and $E(X)=-\sum_{i=1}^{c}{p}_{i} {log}_{2}({p}_{i})$ is the entropy of set $X$, in which ${p}_{i}$ is the probability of class $i$; $\left|{S}_{v}\right|$ is the number of cases with $A={S}_{v}$, and $|S|$ is the number of cases in $A$. Outliers are likely to be ignored by most trees, which makes RF more stable.

Another important feature of RF is its ability to measure the importance of each feature based on the *Gini impurity index*. *Gini impurity* is the likelihood of an incorrect classification of a randomly selected case if it was randomly labeled according to the class distribution of the data. From intuitive perspective, Gini impurity helps the algorithm to decide the optimal split from a root node, and subsequent splits. It is calculated as $G(D)=\sum_{i=1}^{c}p\left(i\right)*(1-p(i))$, where $c$ is the number of classes and $p(i)$ is the relative frequency of class $i$ in $D$. For an attribute ${\theta }_{m}$, if it splits $D$ in to ${D}_{1}$ and ${D}_{2}$, then the Gini index for ${\theta }_{m}$ is ${G}_{{\theta }_{m}}\left(D\right)=\frac{\left|{D}_{1}\right|}{\left|D\right|}G\left({D}_{1}\right)+\frac{\left|{D}_{2}\right|}{\left|D\right|}G\left({D}_{2}\right),$ and the reduction in impurity is $\Delta G\left({\theta }_{m}\right)=G\left(D\right)-{G}_{{\theta }_{m}}\left(D\right)$. A binary DT,${h}_{T}$, is built from a learning sample of size ${n}_{t}$ drawn from $D$ using a recursive procedure, which identifies at each node $t$ the split condition ${s}_{t}={\theta }_{m}<c$ that splits ${n}_{t}$ node samples into ${t}_{L}$, and ${t}_{R}$ maximizes the decrease $\Delta i\left(s,t\right)=i\left(t\right)-pL*i\left({t}_{L}\right)-pR*i({t}_{R})$; $\Delta i$ is the importance of node $t$ based on Gini importance; $pL={n}_{{t}_{L}}$, and $pR={n}_{{t}_{R}}$. For each node split, the Gini impurity index values for the two child nodes are less than the value for the parent node. For each variable, the summation of Gini impurity decreases in a dataset over all trees in the RF model and is the corresponding Gini importance measure for that variable. The global importance of a feature, ${\theta }_{m}$, for predicting $y$ is calculated by adding up the weighted impurity decreases, $p\left(t\right)\Delta i\left({s}_{t},t\right)$, for all nodes $t$ where ${\theta }_{m}$ is used, averaged over all $T$ trees in the forest (see Eq. 3).

3 $$imp\left( {\theta_{m} } \right) = \frac{1}{T}\mathop \sum \limits_{T} \mathop \sum \limits_{{t \in T:v\left( {s_{t} } \right) = \theta_{m} }} p\left( t \right)\Delta i\left( {s_{t} ,t} \right)$$

Interested readers are referred to^56^ for further details about the RF algorithm. More details on the Gini variable importance approach in RF can be found in^87^.

#### Explainability capabilities

**Figure 8:**
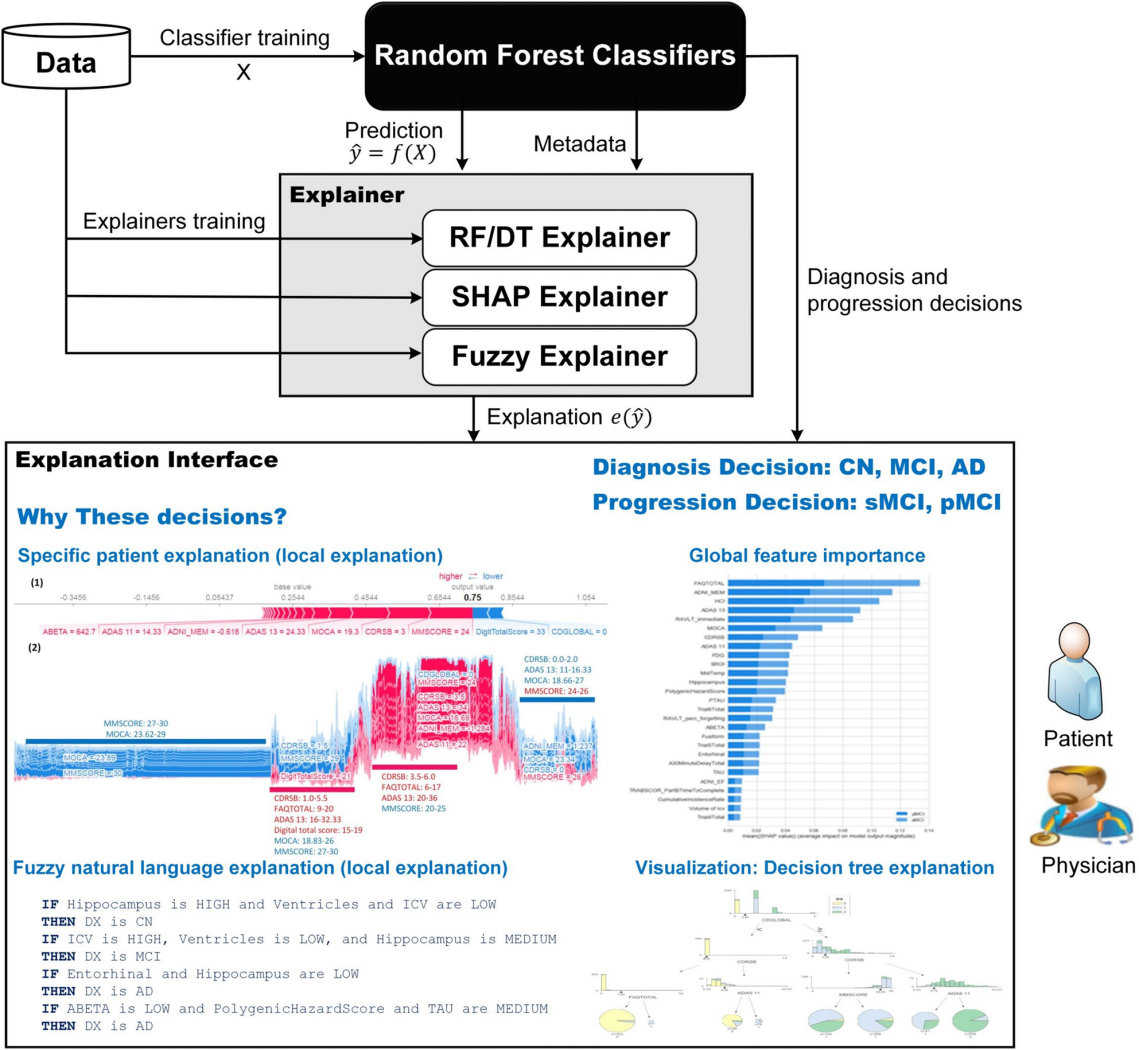
Roles of explainers to enhance RF interpretability.

As RF is an ensemble classifier, it is difficult to get understandable explanation from this complex model. Therefore, we use a collection of simpler models, see Fig. 8, to endow RF with explainability. Each of these models is called “*an explainer*.” These models provide complementary views and explanations associated to the original RF model. Because AD is a complex disease and RF is a complex model, in order to have a global comprehensive, consistent, and accurate picture about AD progression, several explanatory techniques are required^88^. Our explainer framework includes SHAP explainer, DT explainer, and fuzzy explainer. Each of these explainers has been carefully designed to exhibit a good balance between accuracy and explainability. All explainers have been tested to verify they provide physicians with consistent and reliable explanations. As a result, medical expert will be more confident regarding the RF decisions.

For each layer in the proposed model, we provide two types of explanation. *The first type gets explanations from the RF black-box model itself.* For an RF model $b$ and a dataset $D=\left\{\theta ,Y\right\}$, function $f:\left(\theta \to Y\right)\times \left({\theta }^{n}\times {Y}^{n}\right)\to V$ takes $b$ and $D$ as input and returns either global or local approximations $V$ of the behavior of $b$,$f(b,D)=v\in V$, where $V$ is the set of all possible explanations from RF. List $V$ includes explanations regarding both global and local issues. We use *Eli5* to calculate global feature importance based on the Gini index^89,90^, i.e. we compute the level of importance for all features based on the entire set of training data and the RF structure. Because model $b$ is complex, global explanations can sometimes be too approximate to be trustworthy. In addition, medical experts prefer individualized explanations for each specific patient according to his/her own features. Then, we need to take care of local feature contributions too. These explanations, with the contribution directions, are provided for every single patient according to his/her feature vector. We use SHAP tree explainer, which is called the additive feature attribution method^42,91^. SHAP is based on the Shapely value concept from game theory^91,92^. Shapely values are used to estimate the magnitude as sign of feature contributions or importance. It is a theoretically justified and model-agnostic approach that builds a local explanation model,$g$ for the original model $f$. This model is a linear combination of binary variables $g\left({x}^{^{\prime}}\right)={\varnothing }_{0}+\sum_{j=1}^{M}{\varnothing }_{j}{x}_{j}^{^{\prime}}$, where ${x}_{j}^{^{\prime}}$ is a simplified input that map to the original input $x$ using the mapping function ${x=h}_{x}\left({x}^{^{\prime}}\right)$, ${x}^{^{\prime}}\in {\left\{0, 1\right\}}^{M}$ is the coalition vector and the 1 means the features in the new data are the same as those of the original data (the instance $x$), and 0 means the features in the new data are different from those of the original data, $M$ is the total number of features, and ${\varnothing }_{j}\in {\mathbb{R}}$ is the Shapely value that measures the average feature attribution value for feature $j$ for instance $x$. SHAP try to ensure that $g\left({z}^{^{\prime}}\right)\approx f({h}_{x}\left({z}^{^{\prime}}\right))$ when ${z}^{^{\prime}}\approx {x}^{^{\prime}}$. SHAP calculates ${\varnothing }_{j}$ based on the Shapley value from game theory (see Eq. 4)^93^:

4 $$\emptyset_{j} = \mathop \sum \limits_{{S \in \left\{ {x_{1} , \ldots ,x_{M} } \right\}\backslash \left\{ {x_{j} } \right\}}} \frac{{\left| S \right|!\left( {M - \left| S \right| - 1} \right)!}}{M!}\left( {f\left( {S \cup \left\{ {x_{j} } \right\}} \right) - f\left( S \right)} \right)$$

where $S$ is the subset of set of the features used in the model which have non-zero indexes in ${x}^{^{\prime}}$, ${x}^{^{\prime}}$ is the vector of feature values for the instance to be explained, $(\left|S\right|!\left(M-\left|S\right|-1\right)!)/M!$ is a weighting factor, and $f\left(S\right)=E[f(x)|{x}_{S}]$ is the expected value of $f$ for features in subset $S$ that are marginalized over features not included in subset $S$. SHAP values are consistent and accurate because they are calculated by averaging the differences in predictions over every possible feature ordering. In addition, the mean magnitude of the SHAP values can be used to estimate the global feature importance. We will compare the Gini index and SHAP-based methods using our datasets and trained RF classifiers.

Because an individual decision explanation is critical in the medical domain, and because confidence is very important in order to create a trustworthy model, we add another type of explainability. *The second type collects explanations from auxiliary or post-hoc models that try to explain RF decisions.* The explainer is a function $f:\left({\theta }^{m}\to Y\right)\times \left({\theta }^{n\times m}\times {Y}^{n}\right)\to \left({\theta }^{m}\to Y\right)$, which takes $b$, $D$ as input and returns local predictor ${p}_{i}$, i.e.${p}_{i}=f(b,D)$, where ${p}_{i}$ is able to mimic the behavior of $b$; a local explanatory function ${\varepsilon }_{i}:\left(\left({\theta }^{m}\to Y\right)\times \left({\theta }^{m}\times Y\right)\times {\theta }^{m}\right)\to \varepsilon$ exists, for $b$,${p}_{i}$, and ${\theta }^{m}$ instances are inputs; and ${\varepsilon }_{i}$ returns a human interpretable explanation for the patient record ${\theta }^{m}$, i.e.${ \varepsilon }_{i}=f\left(b, {p}_{i},{\theta }^{m}\right)=e$. We implement interpretable classifiers (i.e. DT and FURIA) for each individual modality. These explainers create simple and easy-to-understand explanations from different dimensions (e.g. MRI, cognitive scores, symptoms, etc.), which help to inform domain experts about the oracle’s decision. By using these 22 explainers, we are confident that each oracle’s decision will have a sufficient number of related explanations. The most important thing regarding these 22 explainers is that they are not affected by the feature selection process, which means more features will participate in the explanation. In addition, the extracted formal knowledge from RF and post-hoc models is represented in natural language form by using *ExpliClas*^78^. Accordingly, we resolve the accuracy-explainability trade-off by providing a variety of explanations, while retaining the accuracy of a complex ensemble model (i.e. RF).

### Model performance evaluation metrics

To evaluate the proposed method, we used the following performance metrics: The area under the receiver operating characteristic curve (AUC), precision, recall, accuracy (AC), and F1-score (F1). In addition to the performance evaluation, the system maximizes the interpretability of the underlying models, and pays special attention to explainability, which can serve as an indispensable tool in the era of precision medicine. To validate the performance of the models, we report both cross-validation as well as test results. In each layer, we compared the performance of the best RF model with other ML models, including SVM, KNN, and decision tree models. The hyperparameters of these algorithms were tuned in the same way as RF.

We used several libraries in the Python data science ecosystem to execute the experiments. The *scikit-learn* 0.21.2 package was used to perform feature selection and to train and evaluate all classifiers. *Eli5* 0.8.2 and *SHAP* 0.26.0 were used for explainability, and *ExpliClas* was used to provide natural language explanations from the 22 explainers. The naturalness and acceptability of generated explanations was validated by the physicians who collaborated in our study.

## Concluding remarks

In this paper, we proposed a highly accurate and explainable ML model based on a RF classifier. We have shown that multimodal RF classifiers can be successfully applied to AD detection and progression prediction. We proved that predictions based on combined multimodalities are significantly better than any single modality for both binary and multi-class classification tasks. Based on precise selection of the most informative features from 11 multimodalities, the system achieved the highest accuracies. Explainability was achieved using a variety of techniques. *First*, we provided a set of explanation capabilities for the RF models based on SHAP. For each layer’s model, global feature importance for the whole RF model and feature contributions for each specific patient were provided. For the first layer, we found that MMSE was the most important feature for the AD class, and CDRSB was the most important predictor for CN and MCI classes. For the second layer, FAQ was the most important feature for both sMCI and pMCI classes. *Second*, we implemented 22 explainers for each layer based on a decision tree classifier and a fuzzy rule-based system. Each explainer is based on a single modality. As a result, in each layer, each output decision comes up along with several complementary, consistent and reliable explanations. To validate the effectiveness of our model, we conducted experiments using the ADNI dataset. The model achieved high performance in each layer. The first layer had cross-validation accuracy of 93.95% and an F1-score of 93.94%, and second layer had cross-validation accuracy of 87.08% and an F1-Score of 87.09%. Moreover, our model exhibits a good accuracy-interpretability tradeoff because it achieved very accurate results as well as high level of interpretability. The resulting two-layer model provided justifiable, medically accurate, and hence, actionable decisions that can enhance physician confidence.

The proposed ML model is accurate and explainable. However, it is worth noting that even if we achieved promising results from an academic point of view, we are still far from applying the model in a real-world clinical scenario; what we plan to do in the future. This is a long-term ongoing project. Currently, we are reporting results of the first stage. We have already validated our model with the ADNI dataset; what is a crucial contribution to pave the way towards the application of the model to real clinical data in primary care or general medical practice. Although it is the biggest and most popular real dataset for Alzheimer’s disease, the relevance of our work to direct primary care is limited by the ADNI cohort. Therefore, to translate the outcomes of this study into full-scale clinical practice, further investigations are required to determine its performance characteristics by applying the model to other relevant datasets. We plan to enhance our model with the aim of achieving even higher performance by means of deep learning applied to longitudinal data while preserving explainability issues as we already did in the present manuscript.

### Ethics statement

Data used in this study were obtained from the ADNI (http://adni.loni.usc.edu/). The Alzheimer’s Disease Neuroimaging Initiative Data and Publications Committee (ADNI DPC) coordinates patient enrollment and ensures standard practice on the uses and distribution of the data as follows: The ADNI data were previously collected across 50 research sites. To participate in the study, each study subject gave written informed consent at the time of enrollment for imaging and genetic sample collection and completed questionnaires approved by each participating sites’ Institutional Review Board (IRB). All procedures performed in studies involving human participants were in accordance with the ethical standards of the institutional and/or national research committee and with the 1964 Helsinki declaration and its later amendments or comparable ethical standards. A complete description of ADNI and up-to-date information is available at http://adni.loni.usc.edu/ and data access requests are to be sent to http://adni.loni.usc.edu/data-samples/access-data/. Detailed inclusion criteria for the diagnostic categories can be found at the ADNI website (http://adni.loni.usc.edu/methods). The ethics committees/institutional review board that approved the ADNI study are listed within Supplementary file (part 4).

## Supplementary Information

Supplementary Information.

## Data Availability

The data that support the findings of this study are openly available at the ADNI web site (http://adni.loni.usc.edu/). In addition, the specific patient RIDs used in our study and the full description of used features can be found in the Supplementary Files.

## Abbreviations

AD: Alzheimer’s disease
ADAS-Cog: Features of the Alzheimer’s diseases assessment scale-cognitive
ADNI: Alzheimer’s Disease Neuroimaging Initiative
APOE: Apolipoprotein E
AUC: Area under the ROC curve
AV45: Average AV45 SUVR of frontal
CDSS: Clinical decision support system
CNN: Convolutional neural network
CFA: Cognitive and functional assessments
CS: Cognitive scores
CDRSB: Clinical dementia rating sum of boxes
CDGLOBAL: Global CDR
DL: Deep learning
DT: Decision tree
DARPA: Defense Advanced Research Projects Agency
FRBS: Fuzzy rule-based system
FAQ: Functional assessment questionnaire
GB: Gradient boosting
GDT: Geriatric depression scale
GDPR: General data protection regulation
HCI: Hypometabolic convergence index
ICV: Intracranial volume
KNN: K nearest neighbor
MRI: Magnetic resonance imaging
ML: Machine learning
MCI: Mild cognitive impairment
MCA: Multiclass classification accuracy
MMSE: Mini-Mental State Examination
MoCA: Montreal cognitive assessment
MH: Medical history
NPISCORE: Neuropsychiatric inventory questionnaire score
NB: Neuropsychological battery
NB: Naïve Bayes
pMCI: Progressive MCI
PTAU: Phosphorylated TAU
PET: Positron emission tomography
PCA: Principal component analysis
RF: Random forest
RFE: Recursive feature elimination
RAVLT: Rey Auditory Verbal Learning Test
SHAP: SHapley Additive exPlanations
sMCI: Stable MCI
SVM: Support vector machine
SNOMED CT: Systematized Nomenclature of Medicine-Clinical Terms
XAI: Explainable artificial intelligence
