# Supplementary material for: A multilayer multimodal detection and prediction model based on explainable artificial intelligence for Alzheimer’s disease
Source: Sci Rep. 2021 Jan 29;11:2660. doi: 10.1038/s41598-021-82098-3 (PMC7846613; doi:10.1038/s41598-021-82098-3)
Supplement: Supplementary file 1 — Supplementary Information. [file 41598_2021_82098_MOESM1_ESM.docx]

**Supplementary material**

**A Multilayer Multimodal Detection and Prediction Model based on Explainable Artificial Intelligence for Alzheimer’s Disease**

Shaker El-Sappagh^a,*^, Jose M. Alonso^a^, S. M. Riazul Islam^b^, Ahmad M. Sultan^c^, Kyung Sup Kwak^d,*^,
for the Alzheimer’s Disease Neuroimaging Initiative^1^

^a^ Centro Singular de Investigación en Tecnoloxías Intelixentes (CiTIUS), Universidade de Santiago de Compostela,

15782, Santiago de Compostela, SPAIN; shaker.elsappagh@usc.es (S.E.S.)

^a^ Centro Singular de Investigación en Tecnoloxías Intelixentes, Universidade de Santiago de Compostela, 15703 Santiago, Spain; josemaria.alonso.moral@usc.es (J.M.A.)

^b^ Department of Computer Science and Engineering, Sejong University, 209 Neungdong-ro, Gwangjin-gu, Seoul 05006, Korea; riaz@sejong.ac.kr (S.M.R.I.)

^c^ Gastrointestinal Surgical Center, Faculty of Medicine, Mansoura University, Mansura 35516, Egypt; dr_ahmadsultan@mans.edu.eg (A.M.S.)

^d^ Department of Information and Communication Engineering, Inha University, Incheon 22212, South Korea; kskwak@inha.ac.kr (K.S.K.)

*Correspondence: kskwak@inha.ac.kr (K.S.K); shaker.elsappagh@usc.es (S.E.S.); Tel: +82-32860-7416

**PART 1: THE LIST OF UTILIZED SUBJECT RIDS FROM ADNI DATASET**

| **CN** | **sMCI** | **pMCI** | **AD** |
| --- | --- | --- | --- |
| 011_S_0005 | 022_S_0004 | 023_S_0030 | 011_S_0003 |
| 022_S_0014 | 100_S_0006 | 007_S_0041 | 022_S_0007 |
| 011_S_0016 | 035_S_0033 | 023_S_0042 | 011_S_0010 |
| 067_S_0019 | 130_S_0102 | 067_S_0045 | 067_S_0029 |
| 011_S_0021 | 136_S_0107 | 123_S_0050 | 011_S_0053 |
| 011_S_0023 | 027_S_0116 | 099_S_0054 | 067_S_0076 |
| 023_S_0031 | 137_S_0158 | 018_S_0057 | 023_S_0078 |
| 099_S_0040 | 014_S_0169 | 067_S_0077 | 023_S_0083 |
| 018_S_0043 | 067_S_0176 | 067_S_0098 | 023_S_0084 |
| 100_S_0047 | 128_S_0200 | 007_S_0101 | 123_S_0088 |
| 035_S_0048 | 128_S_0225 | 123_S_0108 | 123_S_0091 |
| 023_S_0058 | 021_S_0273 | 099_S_0111 | 023_S_0093 |
| 022_S_0066 | 067_S_0290 | 023_S_0126 | 123_S_0094 |
| 010_S_0067 | 099_S_0291 | 007_S_0128 | 068_S_0109 |
| 007_S_0068 | 100_S_0296 | 021_S_0141 | 067_S_0110 |
| 007_S_0070 | 027_S_0307 | 018_S_0155 | 022_S_0129 |
| 123_S_0072 | 005_S_0324 | 010_S_0161 | 023_S_0139 |
| 027_S_0074 | 031_S_0351 | 027_S_0179 | 032_S_0147 |
| 023_S_0081 | 037_S_0377 | 032_S_0187 | 098_S_0149 |
| 136_S_0086 | 130_S_0449 | 136_S_0195 | 011_S_0183 |
| 073_S_0089 | 057_S_0464 | 035_S_0204 | 100_S_0190 |
| 099_S_0090 | 068_S_0478 | 032_S_0214 | 136_S_0194 |
| 032_S_0095 | 137_S_0481 | 023_S_0217 | 020_S_0213 |
| 022_S_0096 | 130_S_0505 | 005_S_0222 | 128_S_0216 |
| 020_S_0097 | 005_S_0546 | 128_S_0227 | 005_S_0221 |
| 123_S_0113 | 014_S_0557 | 021_S_0231 | 128_S_0266 |
| 027_S_0118 | 067_S_0607 | 013_S_0240 | 018_S_0286 |
| 027_S_0120 | 128_S_0608 | 011_S_0241 | 136_S_0299 |
| 041_S_0125 | 053_S_0621 | 067_S_0243 | 136_S_0300 |
| 022_S_0130 | 012_S_0634 | 007_S_0249 | 128_S_0310 |
| 021_S_0159 | 027_S_0644 | 027_S_0256 | 007_S_0316 |
| 098_S_0172 | 036_S_0656 | 128_S_0258 | 031_S_0321 |
| 067_S_0177 | 036_S_0673 | 098_S_0269 | 014_S_0328 |
| 136_S_0184 | 126_S_0709 | 130_S_0289 | 021_S_0332 |
| 136_S_0186 | 128_S_0715 | 007_S_0293 | 018_S_0335 |
| 136_S_0196 | 032_S_0718 | 031_S_0294 | 035_S_0341 |
| 067_S_0257 | 073_S_0746 | 041_S_0314 | 021_S_0343 |
| 127_S_0260 | 128_S_0770 | 013_S_0325 | 137_S_0366 |
| 041_S_0262 | 133_S_0771 | 011_S_0326 | 116_S_0370 |
| 128_S_0272 | 002_S_0782 | 023_S_0331 | 099_S_0372 |
| 137_S_0283 | 130_S_0783 | 067_S_0336 | 114_S_0374 |
| 002_S_0295 | 133_S_0792 | 007_S_0344 | 116_S_0392 |
| 123_S_0298 | 137_S_0800 | 011_S_0362 | 032_S_0400 |
| 137_S_0301 | 068_S_0802 | 023_S_0388 | 027_S_0404 |
| 037_S_0303 | 141_S_0851 | 123_S_0390 | 136_S_0426 |
| 082_S_0304 | 031_S_0867 | 127_S_0394 | 127_S_0431 |
| 073_S_0311 | 003_S_0908 | 018_S_0406 | 131_S_0457 |
| 073_S_0312 | 133_S_0912 | 136_S_0429 | 099_S_0470 |
| 072_S_0315 | 053_S_0919 | 094_S_0434 | 057_S_0474 |
| 131_S_0319 | 094_S_0921 | 068_S_0442 | 116_S_0487 |
| 037_S_0327 | 109_S_0950 | 018_S_0450 | 131_S_0497 |
| 021_S_0337 | 022_S_0961 | 068_S_0476 | 128_S_0517 |
| 099_S_0352 | 052_S_0989 | 053_S_0507 | 128_S_0528 |
| 016_S_0359 | 133_S_1031 | 033_S_0511 | 062_S_0535 |
| 018_S_0369 | 099_S_1034 | 033_S_0513 | 022_S_0543 |
| 116_S_0382 | 051_S_1040 | 073_S_0518 | 006_S_0547 |
| 073_S_0386 | 027_S_1045 | 037_S_0539 | 031_S_0554 |
| 027_S_0403 | 023_S_1046 | 014_S_0563 | 073_S_0565 |
| 002_S_0413 | 141_S_1052 | 033_S_0567 | 036_S_0577 |
| 010_S_0419 | 051_S_1072 | 031_S_0568 | 126_S_0606 |
| 018_S_0425 | 128_S_1088 | 005_S_0572 | 002_S_0619 |
| 133_S_0433 | 109_S_1114 | 023_S_0604 | 037_S_0627 |
| 131_S_0441 | 114_S_1118 | 128_S_0611 | 018_S_0633 |
| 010_S_0472 | 003_S_1122 | 023_S_0625 | 021_S_0642 |
| 068_S_0473 | 051_S_1131 | 137_S_0631 | 018_S_0682 |
| 032_S_0479 | 127_S_1140 | 133_S_0638 | 012_S_0689 |
| 133_S_0488 | 002_S_1155 | 082_S_0641 | 062_S_0690 |
| 133_S_0493 | 062_S_1182 | 116_S_0649 | 131_S_0691 |
| 006_S_0498 | 109_S_1183 | 014_S_0658 | 141_S_0696 |
| 128_S_0500 | 126_S_1187 | 098_S_0667 | 012_S_0712 |
| 013_S_0502 | 072_S_1211 | 006_S_0675 | 012_S_0720 |
| 033_S_0516 | 029_S_1218 | 136_S_0695 | 033_S_0724 |
| 014_S_0519 | 136_S_1227 | 141_S_0697 | 062_S_0730 |
| 133_S_0525 | 129_S_1246 | 016_S_0702 | 033_S_0733 |
| 094_S_0526 | 041_S_1260 | 126_S_0708 | 128_S_0740 |
| 099_S_0533 | 057_S_1269 | 033_S_0723 | 100_S_0747 |
| 099_S_0534 | 033_S_1284 | 033_S_0725 | 021_S_0753 |
| 016_S_0538 | 123_S_1300 | 133_S_0727 | 127_S_0754 |
| 005_S_0553 | 094_S_1314 | 002_S_0729 | 036_S_0759 |
| 002_S_0559 | 141_S_1378 | 022_S_0750 | 036_S_0760 |
| 013_S_0575 | 072_S_1380 | 116_S_0752 | 126_S_0784 |
| 036_S_0576 | 029_S_1384 | 016_S_0769 | 010_S_0786 |
| 062_S_0578 | 137_S_1414 | 082_S_0832 | 141_S_0790 |
| 114_S_0601 | 094_S_1417 | 116_S_0834 | 062_S_0793 |
| 005_S_0610 | 041_S_1418 | 027_S_0835 | 137_S_0796 |
| 031_S_0618 | 127_S_1419 | 057_S_0839 | 012_S_0803 |
| 012_S_0637 | 037_S_1421 | 023_S_0855 | 067_S_0812 |
| 057_S_0643 | 016_S_2031 | 011_S_0856 | 005_S_0814 |
| 116_S_0648 | 072_S_2037 | 013_S_0860 | 002_S_0816 |
| 116_S_0657 | 128_S_2045 | 011_S_0861 | 010_S_0829 |
| 036_S_0672 | 123_S_2055 | 126_S_0865 | 029_S_0836 |
| 032_S_0677 | 941_S_2060 | 036_S_0869 | 127_S_0844 |
| 127_S_0684 | 035_S_2061 | 136_S_0873 | 027_S_0850 |
| 002_S_0685 | 099_S_2063 | 136_S_0874 | 141_S_0852 |
| 137_S_0686 | 023_S_2068 | 029_S_0878 | 141_S_0853 |
| 094_S_0692 | 072_S_2072 | 023_S_0887 | 033_S_0889 |
| 094_S_0711 | 035_S_2074 | 100_S_0892 | 126_S_0891 |
| 141_S_0726 | 021_S_2077 | 010_S_0904 | 023_S_0916 |
| 006_S_0731 | 098_S_2079 | 033_S_0906 | 005_S_0929 |
| 033_S_0734 | 072_S_2083 | 073_S_0909 | 002_S_0938 |
| 009_S_0751 | 072_S_2093 | 133_S_0913 | 130_S_0956 |
| 141_S_0767 | 021_S_2100 | 141_S_0915 | 114_S_0979 |
| 062_S_0768 | 153_S_2109 | 033_S_0922 | 016_S_0991 |
| 141_S_0810 | 072_S_2116 | 100_S_0930 | 100_S_0995 |
| 036_S_0813 | 032_S_2119 | 057_S_0941 | 013_S_0996 |
| 057_S_0818 | 082_S_2121 | 052_S_0952 | 029_S_0999 |
| 029_S_0824 | 021_S_2124 | 002_S_0954 | 036_S_1001 |
| 029_S_0845 | 021_S_2125 | 137_S_0973 | 002_S_1018 |
| 009_S_0862 | 021_S_2142 | 036_S_0976 | 141_S_1024 |
| 128_S_0863 | 153_S_2148 | 032_S_0978 | 094_S_1027 |
| 029_S_0866 | 073_S_2153 | 141_S_0982 | 137_S_1041 |
| 130_S_0886 | 018_S_2155 | 132_S_0987 | 053_S_1044 |
| 003_S_0907 | 072_S_2164 | 035_S_0997 | 133_S_1055 |
| 033_S_0923 | 022_S_2167 | 141_S_1004 | 029_S_1056 |
| 003_S_0931 | 027_S_2183 | 057_S_1007 | 003_S_1059 |
| 057_S_0934 | 073_S_2191 | 041_S_1010 | 100_S_1062 |
| 052_S_0951 | 067_S_2196 | 012_S_1033 | 082_S_1079 |
| 023_S_0963 | 109_S_2200 | 128_S_1043 | 027_S_1081 |
| 109_S_0967 | 009_S_2208 | 052_S_1054 | 027_S_1082 |
| 130_S_0969 | 027_S_2219 | 003_S_1057 | 094_S_1090 |
| 003_S_0981 | 128_S_2220 | 031_S_1066 | 014_S_1095 |
| 041_S_1002 | 073_S_2225 | 002_S_1070 | 032_S_1101 |
| 109_S_1014 | 094_S_2238 | 029_S_1073 | 094_S_1102 |
| 033_S_1016 | 024_S_2239 | 126_S_1077 | 021_S_1109 |
| 036_S_1023 | 027_S_2245 | 016_S_1117 | 141_S_1137 |
| 013_S_1035 | 052_S_2249 | 016_S_1121 | 099_S_1144 |
| 033_S_1086 | 073_S_2264 | 023_S_1126 | 141_S_1152 |
| 012_S_1133 | 082_S_2307 | 006_S_1130 | 100_S_1154 |
| 941_S_1195 | 014_S_2308 | 036_S_1135 | 109_S_1157 |
| 941_S_1197 | 027_S_2336 | 016_S_1138 | 013_S_1161 |
| 007_S_1206 | 129_S_2347 | 128_S_1148 | 094_S_1164 |
| 012_S_1212 | 126_S_2360 | 027_S_1213 | 133_S_1170 |
| 007_S_1222 | 123_S_2363 | 057_S_1217 | 024_S_1171 |
| 116_S_1232 | 003_S_2374 | 005_S_1224 | 130_S_1201 |
| 128_S_1242 | 036_S_2380 | 036_S_1240 | 013_S_1205 |
| 052_S_1250 | 013_S_2389 | 116_S_1243 | 031_S_1209 |
| 052_S_1251 | 005_S_2390 | 023_S_1247 | 126_S_1221 |
| 082_S_1256 | 130_S_2391 | 057_S_1265 | 067_S_1253 |
| 094_S_1267 | 114_S_2392 | 116_S_1271 | 027_S_1254 |
| 013_S_1276 | 007_S_2394 | 011_S_1282 | 003_S_1257 |
| 002_S_1280 | 053_S_2396 | 012_S_1292 | 023_S_1262 |
| 100_S_1286 | 126_S_2405 | 941_S_1295 | 033_S_1281 |
| 020_S_1288 | 126_S_2407 | 062_S_1299 | 033_S_1283 |
| 131_S_1301 | 012_S_4012 | 941_S_1311 | 033_S_1285 |
| 094_S_2201 | 031_S_4029 | 116_S_1315 | 130_S_1290 |
| 098_S_4003 | 941_S_4036 | 016_S_1326 | 051_S_1296 |
| 116_S_4010 | 072_S_4063 | 051_S_1331 | 007_S_1304 |
| 041_S_4014 | 129_S_4073 | 022_S_1351 | 024_S_1307 |
| 098_S_4018 | 123_S_4127 | 027_S_1387 | 033_S_1308 |
| 023_S_4020 | 012_S_4128 | 131_S_1389 | 130_S_1337 |
| 031_S_4021 | 041_S_4143 | 024_S_1393 | 007_S_1339 |
| 037_S_4028 | 037_S_4146 | 022_S_1394 | 005_S_1341 |
| 031_S_4032 | 141_S_4160 | 094_S_1398 | 041_S_1368 |
| 041_S_4037 | 024_S_4169 | 128_S_1407 | 057_S_1371 |
| 116_S_4043 | 123_S_4170 | 041_S_1412 | 057_S_1373 |
| 098_S_4050 | 005_S_4185 | 041_S_1423 | 082_S_1377 |
| 041_S_4060 | 116_S_4199 | 041_S_1425 | 057_S_1379 |
| 011_S_4075 | 099_S_4202 | 127_S_1427 | 127_S_1382 |
| 099_S_4076 | 099_S_4205 | 098_S_2047 | 027_S_1385 |
| 003_S_4081 | 072_S_4206 | 094_S_2216 | 094_S_1397 |
| 035_S_4082 | 127_S_4210 | 068_S_2248 | 094_S_1402 |
| 024_S_4084 | 073_S_4216 | 011_S_2274 | 128_S_1409 |
| 099_S_4086 | 129_S_4220 | 068_S_2316 | 128_S_1430 |
| 082_S_4090 | 072_S_4226 | 009_S_2381 | 037_S_4001 |
| 072_S_4103 | 002_S_4229 | 057_S_2398 | 016_S_4009 |
| 099_S_4104 | 011_S_4235 | 031_S_4005 | 031_S_4024 |
| 011_S_4105 | 041_S_4271 | 037_S_4015 | 014_S_4039 |
| 003_S_4119 | 007_S_4272 | 023_S_4035 | 094_S_4089 |
| 011_S_4120 | 135_S_4281 | 031_S_4042 | 003_S_4136 |
| 153_S_4125 | 129_S_4287 | 072_S_4057 | 003_S_4152 |
| 127_S_4148 | 130_S_4294 | 014_S_4058 | 006_S_4153 |
| 006_S_4150 | 137_S_4299 | 014_S_4079 | 153_S_4172 |
| 153_S_4151 | 073_S_4300 | 012_S_4094 | 006_S_4192 |
| 024_S_4158 | 127_S_4301 | 123_S_4096 | 116_S_4195 |
| 023_S_4164 | 135_S_4309 | 072_S_4102 | 137_S_4211 |
| 022_S_4173 | 073_S_4311 | 035_S_4114 | 098_S_4215 |
| 033_S_4176 | 073_S_4312 | 072_S_4131 | 024_S_4223 |
| 033_S_4177 | 003_S_4354 | 094_S_4162 | 019_S_4252 |
| 022_S_4196 | 135_S_4356 | 116_S_4167 | 137_S_4258 |
| 041_S_4200 | 073_S_4360 | 002_S_4171 | 024_S_4280 |
| 082_S_4208 | 072_S_4383 | 136_S_4189 | 094_S_4282 |
| 002_S_4213 | 072_S_4390 | 031_S_4203 | 029_S_4307 |
| 011_S_4222 | 072_S_4394 | 127_S_4240 | 116_S_4338 |
| 082_S_4224 | 013_S_4395 | 023_S_4243 | 003_S_4373 |
| 021_S_4254 | 130_S_4417 | 130_S_4250 | 114_S_4379 |
| 941_S_4255 | 073_S_4443 | 014_S_4263 | 019_S_4477 |
| 022_S_4266 | 002_S_4447 | 019_S_4293 | 126_S_4494 |
| 021_S_4276 | 109_S_4455 | 137_S_4303 | 127_S_4500 |
| 029_S_4279 | 099_S_4463 | 009_S_4324 | 023_S_4501 |
| 022_S_4291 | 072_S_4465 | 011_S_4366 | 123_S_4526 |
| 941_S_4292 | 007_S_4467 | 021_S_4402 | 019_S_4549 |
| 037_S_4308 | 002_S_4473 | 135_S_4406 | 007_S_4568 |
| 018_S_4313 | 031_S_4476 | 035_S_4414 | 016_S_4591 |
| 022_S_4320 | 135_S_4489 | 130_S_4415 | 014_S_4615 |
| 021_S_4335 | 099_S_4498 | 141_S_4426 | 116_S_4625 |
| 082_S_4339 | 041_S_4510 | 036_S_4430 | 135_S_4657 |
| 068_S_4340 | 126_S_4514 | 037_S_4432 | 130_S_4660 |
| 130_S_4343 | 072_S_4522 | 126_S_4458 | 137_S_4672 |
| 010_S_4345 | 109_S_4531 | 023_S_4502 | 135_S_4676 |
| 018_S_4349 | 137_S_4536 | 126_S_4507 | 126_S_4686 |
| 003_S_4350 | 072_S_4539 | 006_S_4515 | 018_S_4696 |
| 130_S_4352 | 019_S_4548 | 009_S_4530 | 005_S_4707 |
| 006_S_4357 | 128_S_4553 | 130_S_4542 | 021_S_4718 |
| 019_S_4367 | 053_S_4557 | 016_S_4584 | 067_S_4728 |
| 129_S_4369 | 128_S_4571 | 013_S_4595 | 130_S_4730 |
| 129_S_4371 | 031_S_4590 | 137_S_4596 | 116_S_4732 |
| 153_S_4372 | 109_S_4594 | 053_S_4661 | 018_S_4733 |
| 072_S_4391 | 007_S_4611 | 014_S_4668 | 032_S_4755 |
| 094_S_4503 | 072_S_4613 | 126_S_4675 | 137_S_4756 |
| 127_S_4604 | 073_S_4614 | 019_S_4680 | 037_S_4770 |
| 009_S_4612 | 153_S_4621 | 135_S_4689 | 128_S_4772 |
| 013_S_4616 | 052_S_4626 | 126_S_4712 | 128_S_4774 |
| 007_S_4620 | 094_S_4630 | 036_S_4715 | 035_S_4783 |
| 137_S_4632 | 116_S_4635 | 027_S_4729 | 027_S_4801 |
| 007_S_4637 | 128_S_4636 | 009_S_4741 | 027_S_4802 |
| 012_S_4643 | 128_S_4653 | 027_S_4757 | 036_S_4820 |
| 003_S_4644 | 002_S_4654 | 035_S_4784 | 011_S_4827 |
| 127_S_4645 | 024_S_4674 | 023_S_4796 | 073_S_4853 |
| 094_S_4649 | 137_S_4678 | 137_S_4815 | 135_S_4863 |
| 073_S_4739 | 006_S_4679 | 137_S_4816 | 006_S_4867 |
| 073_S_4762 | 135_S_4722 | 021_S_4857 | 037_S_4879 |
| 035_S_4785 | 135_S_4723 | 057_S_4888 | 016_S_4887 |
| 073_S_4795 | 036_S_4736 | 016_S_4902 | 003_S_4892 |
| 128_S_4832 | 128_S_4742 | 067_S_4918 | 036_S_4894 |
| 019_S_4835 | 021_S_4744 | 127_S_4928 | 024_S_4905 |
| 127_S_4843 | 941_S_4764 | 051_S_4929 | 011_S_4906 |
| 070_S_4856 | 123_S_4780 | 027_S_4936 | 005_S_4910 |
| 003_S_4872 | 067_S_4782 | 052_S_4945 | 007_S_4911 |
| 036_S_4878 | 027_S_4804 | 031_S_4947 | 011_S_4912 |
| 003_S_4900 | 123_S_4806 | 009_S_4958 | 027_S_4938 |
| 016_S_4951 | 018_S_4809 | 041_S_5026 | 127_S_4940 |
| 016_S_4952 | 130_S_4817 | 016_S_5031 | 011_S_4949 |
| 073_S_5023 | 153_S_4838 |  | 135_S_4954 |
| 070_S_5040 | 012_S_4849 |  | 052_S_4959 |
| 041_S_5078 | 094_S_4858 |  | 027_S_4962 |
| 027_S_5079 | 018_S_4868 |  | 027_S_4964 |
| 041_S_5082 | 027_S_4869 |  | 068_S_4968 |
| 027_S_5083 | 072_S_4871 |  | 130_S_4971 |
| 100_S_5091 | 041_S_4876 |  | 051_S_4980 |
| 027_S_5093 | 018_S_4889 |  | 130_S_4982 |
| 041_S_5100 | 126_S_4891 |  | 130_S_4984 |
| 027_S_5109 | 126_S_4896 |  | 130_S_4990 |
| 027_S_5110 | 116_S_4898 |  | 127_S_4992 |
| 135_S_5113 | 027_S_4919 |  | 051_S_5005 |
| 027_S_5118 | 027_S_4926 |  | 130_S_5006 |
| 941_S_5124 | 072_S_4941 |  | 019_S_5012 |
| 037_S_5126 | 027_S_4966 |  | 033_S_5013 |
| 027_S_5127 | 073_S_4986 |  | 135_S_5015 |
| 003_S_5130 | 012_S_4987 |  | 033_S_5017 |
| 041_S_5131 | 041_S_4989 |  | 002_S_5018 |
| 127_S_5132 | 009_S_5000 |  | 019_S_5019 |
| 013_S_5137 | 022_S_5004 |  | 009_S_5027 |
| 020_S_5140 | 114_S_5047 |  | 127_S_5028 |
| 041_S_5141 | 128_S_5066 |  | 082_S_5029 |
| 130_S_5142 |  |  | 016_S_5032 |
| 003_S_5154 |  |  | 009_S_5037 |
| 012_S_5157 |  |  | 005_S_5038 |
| 029_S_5158 |  |  | 024_S_5054 |
| 067_S_5159 |  |  | 127_S_5056 |
| 073_S_5167 |  |  | 016_S_5057 |
| 027_S_5169 |  |  | 127_S_5058 |
| 027_S_5170 |  |  | 052_S_5062 |
| 013_S_5171 |  |  | 036_S_5063 |
| 130_S_5175 |  |  | 127_S_5067 |
| 009_S_5176 |  |  | 053_S_5070 |
| 021_S_5177 |  |  | 013_S_5071 |
| 002_S_5178 |  |  | 033_S_5087 |
| 127_S_5185 |  |  | 007_S_5196 |
| 941_S_5193 |  |  |  |
| 021_S_5194 |  |  |  |
| 012_S_5195 |  |  |  |
| 027_S_5197 |  |  |  |
| 033_S_5198 |  |  |  |
| 057_S_5199 |  |  |  |
| 127_S_5200 |  |  |  |
| 053_S_5202 |  |  |  |
| 020_S_5203 |  |  |  |
| 012_S_5213 |  |  |  |
| 037_S_5222 |  |  |  |
| 127_S_5228 |  |  |  |
| 002_S_5230 |  |  |  |
| 021_S_5236 |  |  |  |
| 126_S_5243 |  |  |  |
| 041_S_5253 |  |  |  |
| 130_S_5258 |  |  |  |
| 033_S_5259 |  |  |  |
| 007_S_5265 |  |  |  |
| 127_S_5266 |  |  |  |
| 053_S_5272 |  |  |  |
| 082_S_5282 |  |  |  |
| 027_S_5288 |  |  |  |
| 032_S_5289 |  |  |  |
| 024_S_5290 |  |  |  |
| 057_S_5292 |  |  |  |

**PART 2: DATA SET DESCRIPTION AND RESULTS**

**2.A DESCRIPTION OF THE UTILIZED MODALITIES**

The following is a detailed description of the data used. The selected features are the most famous features in the literature, and the recommended features by the ADNI. These multimodalities considered include cognitive scores, demographics, medical history, symptoms, vital signs, MRI, PET, genetics, lab tests, and neuropsychological battery. Each domain is described in more detail in what follows. In the following: (N) means the feature is numeric, and (YES/NO) means that the feature has only two values: YES or NO.

1. *Cognitive scores*

- ADAS-Cog 11: (N) Alzheimer Disease Assessment Scale–Cognitive Subscale (11 items)
- ADAS-Cog 13: (N) Alzheimer Disease Assessment Scale–Cognitive Subscale (13 items)
- ADNI_MEM: (N) UW - Neuropsych Summary Scores, Score 1 (composite score for memory)
- ADNI_EF: (N) UW - Neuropsych Summary Scores, Score 2 (composite score for executive functioning)
- CDGLOBAL: (N) Clinical Dementia Rating (CDR) Global Score
- CDRSB: (N) CDR sum of box score
- FAQTOTAL: (N) Functional Activities Questionnaire (FAQ) Total Score
- GDTOTAL: (N) Geriatric Depression Scale (GDS) Total Score
- HMSCORE: (N) Modified Hachinski Ischemia Total Score
- MMSCORE: (N) Mini-Mental State Examination (MMSE) Total Score
- MOCA: (N) Montreal Cognitive Assessment (MoCA)
- NPISCORE: (N) Neuropsychiatric Inventory Questionnaire (NPI-Q) Score

1. *Genetics*

- ABETA: (N) Amyloid beta
- PTAU: (N) Tau phosphorylated at specific epitopes in cerebrospinal fluid
- TAU: (N) Tau proteins
- PolygenicHazardScore: (N) Desikan Lab Polygenic Hazard Score (PHS)
- CumulativeIncidenceRate: (N) Desikan Lab Cumulative Incidence Rate

1. *Lab tests*

- CTRED: (N) Red blood cell count
- CTWHITE: (N) White blood cell count
- PROTEIN: (N) Protein results
- GLUCOSE: (N) Glucose results
- BAT126: (N) Test BAT126; Vitamin B12
- HMT10: (N) Test HMT10; Monocytes
- HMT100: (N) Test HMT100; MCH
- HMT102: (N) Test HMT102; MCHC
- HMT11: (N) Test HMT11; Eosinophils
- HMT12: (N) Test HMT12; Basophils
- HMT13: (N) Test HMT13; Platelets
- HMT15: (N) Test HMT15; Neutrophils (%)
- HMT16: (N) Test HMT16; Lymphocytes (%)
- HMT17: (N) Test HMT17; Monocytes (%)
- HMT18: (N) Test HMT18; Eosinophils (%)
- HMT19: (N) Test HMT19; Basophils (%)
- HMT2: (N) Test HMT2; Hematocrit
- HMT3: (N) Test HMT3; RBC
- HMT4: (N) Test HMT4; MCV
- HMT40: (N) Test HMT40; Hemoglobin
- HMT7: (N) Test HMT7; WBC
- HMT8: (N) Test HMT8; Neutrophils
- HMT9: (N) Test HMT9; Lymphocytes
- RCT1: (N) Test RCT1; Total Bilirubin
- RCT11: (N) Test RCT11; Serum Glucose
- RCT12: (N) Test RCT12; Total Protein
- RCT13: (N) Test RCT13; Albumin
- RCT14: (N) Test RCT14; Creatine Kinase
- RCT1407: (N) Test RCT1407; Alkaline Phosphatase
- RCT1408: (N) Test RCT1408; LDH
- RCT183: (N) Test RCT183; Calcium (EDTA)
- RCT19: (N) Test RCT19; Triglycerides (GPO)
- RCT20: (N) Test RCT20; Cholesterol (High Performance)
- RCT29: (N) Test RCT29; Direct Bilirubin
- RCT3: (N) Test RCT3; GGT
- RCT5: (N) Test RCT5; AST (SGOT)
- RCT6: (N) Test RCT6; Urea Nitrogen
- RCT9: (N) Test RCT9; Phosphorus
- RCT392: (N) Test RCT392; Creatinine (Rate Blanked)
- RCT4: (N) Test RCT4; ALT (SGPT)
- RCT8: (N) Test; Serum Uric Acid

1. *Medical history*

- FHQDAD: (Yes/No) Patient’s father had dementia
- FHQDADAD: (Yes/No) Patient’s father had Alzheimer’s
- FHQMOM: (Yes/No) Patient’s mother had dementia
- FHQMOMAD: (Yes/No) Patient’s mother had Alzheimer’s
- MH2NEURL: (Yes/No) Patient has a neurologic problem other than Alzheimer’s
- MH3HEAD: (Yes/No) Patient has head, eyes, ears, nose, or throat problem
- MH4CARD: (Yes/No) Patient has a cardiovascular problem
- MH5RESP: (Yes/No) Patient has a respiratory problem
- MH6HEPAT: (Yes/No) Patient has a hepatic problem
- MH7DERM: (Yes/No) Patient has a dermatologic connective tissue problem
- MH8MUSCL: (Yes/No) Patient has a musculoskeletal problem
- MH9ENDO: (Yes/No) Patient has an endocrine metabolic problem
- MH10GAST: (Yes/No) Patient has a gastrointestinal problem
- MH11HEMA: (Yes/No): Patient has a hematopoietic lymphatic problem
- MH12RENA: (Yes/No) Patient has a renal genitourinary problem
- MH13ALLE: (Yes/No) Patient has allergies or drug sensitivities
- MH14ALCH: (Yes/No): Patient has an alcohol abuse problem
- MH15DRUG: (Yes/No) Patient has a drug abuse problem
- MH16SMOK: (Yes/No) Patient is a smoker
- MH17MALI: (Yes/No) Patient has a malignancy
- MH18SURG: (Yes/No) Patient had major surgical procedures
- MHPSYCH: (Yes/No) Patient has a psychiatric problem
- AGE: (N) Age
- APOE4: (N) APOE4
- PTMARRY: (C) Marital status
- PTGENDER: Gender (Male/Female)
- PTEDUCAT: (N) Number of years of education

1. *Magnetic Resonance Imaging*

- Entorhinal: (N) Entorhinal volume
- Fusiform: (N) Fusiform volume
- Hippocampus: (N) Hippocampus volume
- MidTemp: (N) Temporal volume
- Ventricles: (N) Sum of ventricles volume
- Volume of ICV: (N) Total intracranial volume
- Whole brain volume: (N) Volume of the brain

1. *Neuro exams*

- NXAUDITO: (Yes/No) Patient has significant auditory impairment
- NXCONSCI: (Yes/No) Patient has normal level of consciousness
- NXFINGER: (Yes/No) Patient passed the cerebellar finger-to-nose test
- NXGAIT: (Yes/No) Patient has a normal gait
- NXHEEL: (Yes/No) Patient passed the asymmetric heel-to-shin test
- NXMOTOR: (Yes/No) Patient has a motor strength problem
- NXNERVE: (Yes/No) Patient has a cranial nerve problem
- NXPLANTA: (Yes/No) Patient has a plantar reflexes problem
- NXSENSOR: (Yes/No) Patient has a sensory problem
- NXTENDON: (Yes/No) Patient has a deep tendon reflex problem
- NXTREMOR: (Yes/No) Patient has tremors
- NXVISUAL: (Yes/No) Patient has significant visual impairment

1. *Neuropsychological battery*

- AVDEL30MIN: (N) 30-minute delay total
- AVDELTOT: (N) Recognition score
- Trial1Total: (N) Trial 1 total
- Trial2Total: (N) Trial 2 total
- Trial3Total: (N) Trial 3 total
- Trial4Total: (N) Trial 4 total
- Trial5Total: (N) Trial 5 total
- Trial6Total: (N) Trial 6 total
- ListBTotal: (N) List B total
- CATANIMSC: (N) Category fluency: animals total correct
- CATANINTR: (N) Category fluency: animal intrusions
- CATVGPERS: (N) Category fluency: animals perseverations
- CLOCKCIRC: (Yes/No) Clock: approximately circular face
- CLOCKHAND: (Yes/No) Clock: presence of the two hands
- CLOCKNUM: (Yes/No) Clock: correctness of numbers
- CLOCKSCOR: (N) Clock: total score
- CLOCKSYM: (Yes/No) Clock: symmetry of number placement
- CLOCKTIME: (Yes/No) Clock: presence of the two hands set to ten after eleven
- COPYCIRC: (Yes/No) Copy: approximately circular face
- COPYHAND: (Yes/No) Copy: presence of the two hands
- COPYNUM: (Yes/No) Copy: correctness of numbers
- COPYSCOR: (N) Copy: total score
- COPYSYM: (Yes/No) Copy: symmetry of number placement
- COPYTIME: (Yes/No) Copy: presence of the two hands set to ten after eleven
- DIGITSCOR: (N) Digit total score
- RAVLT forgetting: (N) Rey Auditory Verbal Learning Test (RAVLT) forgetting
- RAVLT_immediate: (N) RAVLT immediate
- RAVLT learning: (N) RAVLT learning
- RAVLT percentage forgetting: (N) RAVLT percentage forgetting
- TRABERRCOM: (N) Errors of commission
- TRABERROM: (N) Errors of omission part A
- TRAASCOR: (N) Part A time to complete
- TRABERRCOM: (N) Errors of commission
- TRABERROM: (N) Errors of omission part B
- TRABSCOR: (N) Part B time to complete

1. *Positron emission tomography*

- FDG: (N) Fluorodeoxyglucose (FDG), sum of mean glucose metabolism uptake in regions of angular, temporal, and posterior cingulate
- HCI : (N) Hypometabolic convergence index
- SROI: (N) Statistical region of interest developed for optimal longitudinal FDG- positron emission tomography (PET) CMRgl changes

1. *Physical exams*

- PXGENAPP: (Yes/No) Patient has a general appearance problem
- PXNECK: (Yes/No) Patient has a neck problem
- PXCHEST: (Yes/No) Patient has a chest problem
- PXHEART: (Yes/No) Patient has a heart problem
- PXABDOM: (Yes/No) Patient has an abdomen problem
- PXEXTREM : (Yes/No) Patient has an extremities problem
- PXPERIPH: (Yes/No) Patient has a peripheral vascular problem
- PXSKIN: (Yes/No) Patient has a skin and/or appendages problem

1. *Symptoms*

- BCNAUSEA: (Yes/No) Nausea
- BCVOMIT: (Yes/No) Vomiting
- BCDIARRH: (Yes/No) Diarrhea
- BCCONSTP: (Yes/No) Constipation
- BCABDOM: (Yes/No) Abdominal discomfort
- BCSWEATN: (Yes/No) Sweating
- BCDIZZY: (Yes/No) Dizziness
- BCENERGY: (Yes/No) Low energy
- BCDROWSY: (Yes/No) Drowsiness
- BCVISION: (Yes/No) Blurred vision
- BCHDACHE: (Yes/No) Headache
- BCDRYMTH: (Yes/No) Dry mouth
- BCBREATH: (Yes/No) Shortness of breath
- BCCOUGH: (Yes/No) Coughing
- BCPALPIT: (Yes/No) Palpitations
- BCCHEST: (Yes/No) Chest pain
- BCURNDIS: (Yes/No) Urinary discomfort (e.g., burning)
- BCURNFRQ: (Yes/No) Abnormal urinary frequency
- BCANKLE: (Yes/No) Ankle swelling
- BCRASH: (Yes/No) Rash
- BCINSOMN : (Yes/No) Insomnia
- BCDPMOOD: (Yes/No) Depressed mood
- BCCRYING: (Yes/No) Crying
- BCELMOOD: (Yes/No) Elevated mood
- BCWANDER: (Yes/No) Wandering
- BCFALL: (Yes/No) Fall

1. *Vital signs*

- VSBPDIA: (N) Diastolic blood pressure (mmHg)
- VSBPSYS: (N) Systolic blood pressure (mmHg)
- VSHEIGHT: (N) Height
- VSPULSE: (N) Seated pulse rate (per minute)
- VSRESP: (N) Respiratory rate (per minute)
- VSTEMP (N) Temperature
- VSWEIGHT: (N) Weight
- BMI: (N) Body mass index

**2.B DESCRIPTION OF THE CALCULATED FEATURES**

The following is a description of the calculated features based on the previously described features. All details about the ADNI dataset can be found at *adni.loni.usc.edu/* and *https://adni.bitbucket.io/reference/index.html*.

1. Magnetic resonance imaging (MRI) biomarkers are used for Alzheimer’s disease (AD) progression modeling. We extracted a set of summary features from MRI images including:

- *Whole brain volume*= sum (ST128SV+ST17SV+ST18SV+ST19SV+ST20SV+ST61SV+ST16SV+ ST53SV+ST42SV+ST29SV+ST12SV+ST11SV+ST65SV+ST76SV+ST77SV+ST78SV+ST79SV+ST120SV+ST75SV+ST112SV+ST101SV+ST88SV+ST71SV+ST70SV+ST124SV)
- *Hippocampus* = ST29SV+ST88SV
- *Entorhinal* = ST24CV+ST83CV
- *Fusiform* = ST26CV+ST85CV
- *MidTemp* = ST40CV+ST99CV
- *Ventricles* = ST30SV+ST37SV+ST89SV+ST96SV

1. Four neuropsychological features are calculated as follows:

- *RAVLT forgetting*: AVTOT5 - AVDEL30MIN
- *RAVLT immediate*: AVTOT1 + AVTOT2 + AVTOT3 + AVTOT4 + AVTOT5
- *RAVLT learning*: AVTOT5 - AVTOT1
- *RAVLT percentage forgetting*: 100 * RAVLT forgetting / AVTOT5

1. Body Mass Index (BMI) is calculated as follows:

- BMI = weight in KGs/(height in meters)^2^

**2.C SELECTED FEATURES FOR EACH LAYER**

According to the feature selection step, Table T1 describes the selected features in each layer.

| **Table T1.** Feature selection results for both layers |
| --- |
| **(A) Selected Features for the First Layer: 28 Features** |
| - **Cognitive scores**= {‘MOCA’, ‘MMSCORE’, ‘CDRSB’, ‘ADAS 13’, ‘CDGLOBAL’, ‘FAQTOTAL’, ‘ADAS 11’, ‘ADNI_MEM’} - **MRI**= {‘Hippocampus’, ‘Entorhinal’} - **PET**= {‘FDG’, ‘HCI’, ‘SROI’} - **Genetics**= {‘ABETA’, ‘TAU’, ‘PTAU’, ‘PolygenicHazardScore’, ‘CumulativeIncidenceRate’} - **Neuropsychological battery**= {‘RAVLT_immediate’, ‘DigitTotalScore’, ‘Trial6Total’, ‘Trial5Total’, ‘TRABSCOR_PartBTimeToComplete’, ‘A30MinuteDelayTotal’} - **Medical history**= {‘AGE’, ‘Gender’, ‘FatherHadAlzheimer’, ‘HMT17_Mo0cytesPercentage’} |
| **(B) Selected Features for the Second Layer: 36 Features** |
| - **Cognitive scores** = {'ADAS 11', 'ADAS 13', 'ADNI_MEM', 'ADNI_EF', 'CDRSB', 'FAQTOTAL', 'MOCA'} - **Neuropsychological battery**= {'Trial4Total', 'Trial5Total','Trial6Total', 'RAVLT_immediate', 'RAVLT_perc_forgetting', 'TRABSCOR_PartBTimeToComplete', 'A30MinuteDelayTotal'} - **MRI**= {'Fusiform', 'Hippocampus', 'Volume of ICV', 'MidTemp', 'Entorhinal'} - **PET**= {'FDG', 'HCI', 'SROI'} - **Genetics**= {'ABETA', 'PTAU', 'TAU', 'PolygenicHazardScore', 'CumulativeIncidenceRate'} - **MH**= {'AGE', 'BMI', 'HMT8_Neutrophils', 'HMT15_NeutrophilsPercentage', 'HMT16_LymphocytesPercentage', 'HMT18_EosinophilsPercentage', 'RCT14_CreatineKinase', 'RCT1408_LDH', 'Weight'} |

**2.D RANDOM FOREST MODEL EXPLAINABILITY**

Figure S1 shows the global feature importance for 19 features of the first layer and 27 features of the second layer. These values are based on the SHAP values of each feature, and the values are distributed according to the classes. For the first layer, the most important feature is CDRSB, and the lowest important feature is TRABSCOR_PartBTimeToComplete. For the second layer, FAQTOTAL is the most critical feature, and Trial4Total is the lowest important feature. Table T2 shows the combined feature importance for the model, averaged over all classes, for the first layer and the second layer. Figure S2 shows two examples of prediction in the second layer. In Figure S2 (A) part (1), the system has 90% confidence that the case is progressive mild cognitive impairment (pMCI). As can be seen, ADAS 13= 31 and FAQTOTAL= 5 have the main effect in pushing the classification towards pMCI. Other features, including ADNI_MEM= -0.535, HCI= 15.25, RAVLT immediate= 27, ADAS 11= 22, Hippocampus= 5714, etc., have a visible effect on the model, pushing it towards the pMCI class. On the other hand, MOCA=24 and PolygenicHazardScore= -0.8 have a main role in pushing the decision against the pMCI class.

Figure S2 (A) part (2) clusters the behavior of the model according to the values of critical features. For example, for a case with the features MedTemp= 2.2, FAQTOTAL=0, Hippocampus= 7920, and HCI= 7.445, the system will not select the pMCI class. On the other hand, for a case having the values FAQTOTAL= 10, CDRSB= 3, SROI= 1.139, ADAS 13= 18, and ADNI_MEM= ‑0.231, the system returns HIGH in selecting the pMCI class. As a result, the medical expert is able to know how the model calculates, and why it takes specific decisions. Figure S2 (B) part (1) shows a prediction example of stable MCI (sMCI), and Figure S2 (B) part (2) shows the clustering of cases according to the feature contributions for the class (in red) and against the class (in blue).

Although some features like PTAU are listed as important features in Figure S1, their role in each class prediction is noisy (see Figure 5). In addition, some features are at the bottom of the list in Figure S1 (e.g. Entorhinal), which means they may have less effect on the model’s decisions. To understand the nature of these features and their real roles in the model, we need to zoom in and study their behavior (alone and in combination with other features). Figure S3 provides an example of this analysis for the PTAU feature in the first layer and for Entorhinal in the second layer.


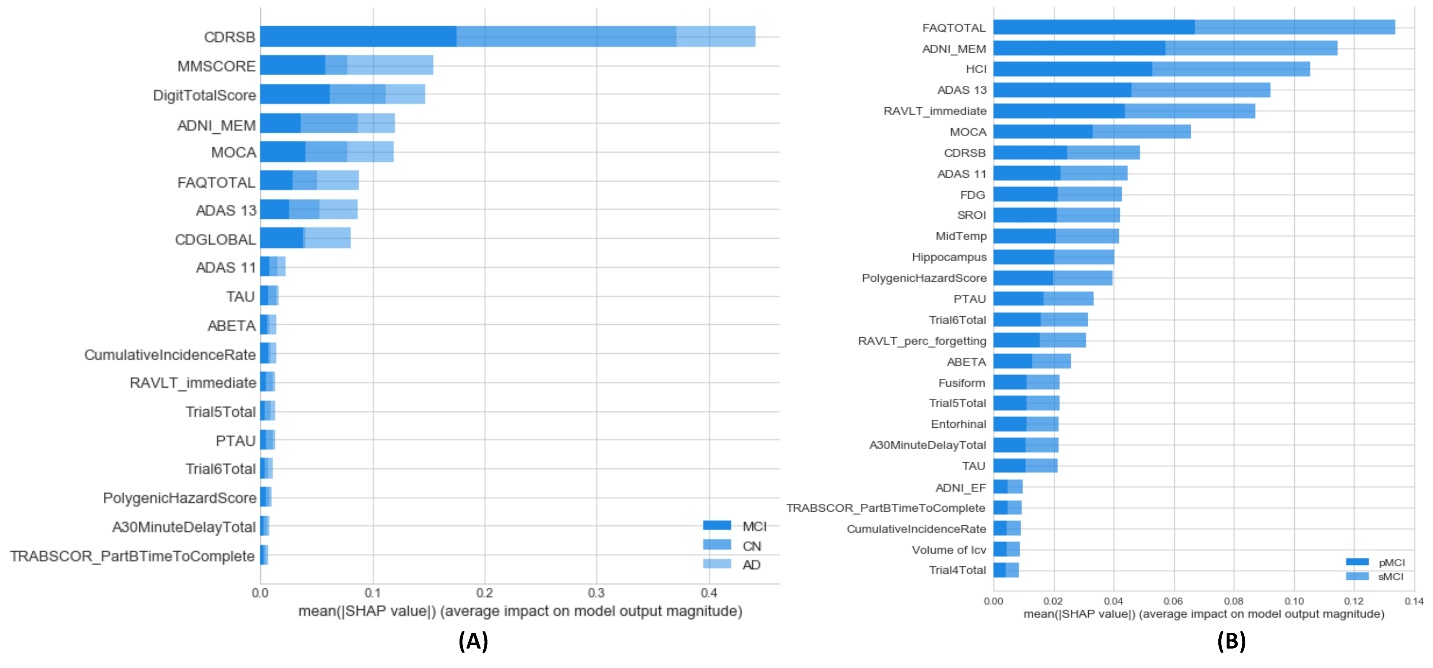


**Figure S1.** Global feature importance based on the SHAP values for every layer and for each class.

We use the SHAP dependence contribution plots, which are similar to partial dependency plots, but we add more details. Figure S3 (1) plots the dependence relationship between PTAU and the most correlated feature: ADNI_MEM. It is easy to see that small values for PTAU with large values for ADNI_MEM have a positive impact towards the CN class. PTAU around 40 with HIGH ADNI_MEM values also have a positive impact towards the CN class, but low values of ADNI_MEM have a negative impact on the same class. On the other hand, low values for PTAU have a negative impact for the MCI class, as seen in Figure S3 (2), and PTAU around 40 has an inverse effect for the CN class. This is medically intuitive. For the AD class, as seen in Figure S3 (3), the SHAP values show dispersion due to feature interaction. This dispersion can be captured by using the Shapley interaction index.

| **Table T2.** Global feature importance based on SHAP values averaged over all classes for each layer | | | |
| --- | --- | --- | --- |
| **First Layer** | | **Second Layer** | |
| Feature name | Feature importance | Feature name | Feature importance |
| CDRSB  DigitTotalScore  MMSCORE  MOCA  FAQTOTAL  ADNI_MEM  ADAS 13  CDGLOBAL  ADAS 11  PTAU  TAU  ABETA  RAVLT_immediate  Trial6Total  CumulativeIncidenceRate  A30MinuteDelayTotal  PolygenicHazardScore  Trial5Total  TRABSCOR_PartBTimeToComplete | 0.12204700  0.05745940  0.05625770  0.04462080  0.03805050  0.03618750  0.02890950  0.02162120  0.01294330  0.00744985  0.00661905  0.00559975  0.00534061  0.00526021  0.00518833  0.00443709  0.00377049  0.00290747  0.00252967 | FAQTOTAL  ADNI_MEM  HCI  ADAS 13  RAVLT_immediate  MOCA  CDRSB  ADAS 11  FDG  SROI  MidTemp  Hippocampus  PolygenicHazardScore  PTAU  Trial6Total  RAVLT_perc_forgetting  ABETA  Fusiform  Trial5Total  Entorhinal  A30MinuteDelayTotal  TAU  ADNI_EF  TRABSCOR_PartBTimeToComplete  CumulativeIncidenceRate  Volume of ICV  Trial4Total | 0.0668579  0.0572493  0.0527069  0.0460380  0.0436037  0.0328886  0.0244004  0.0222991  0.0213570  0.0210909  0.0208894  0.0201799  0.0198271  0.0166814  0.0157308  0.0153791  0.0129552  0.0109841  0.0109419  0.0108784  0.0108222  0.0107518  0.0048170  0.0047846  0.0045062  0.0044386  0.0042562 |

The SHAP interaction index between two features$i, j$ is defined as$\emptyset_{i, j}=\sum_{S\in\{x_{1},\ldots,x_{M}\}\backslash\{x_{i}, x_{j}\}} \frac{\left| S \right|!\left( M-\left| S \right|-2 \right)!}{M!}\nabla_{i,j}(S)$, where$\nabla_{i,j}\left( S \right)=f_{x}\left( S \bigcup\left\{ x_{i}, x_{j} \right\} \right)-f_{x}\left( S\cup\left\{ x_{j} \right\} \right)-\left[ f_{x}\left( S\bigcup\left\{ x_{i} \right\} \right)-f_{x}\left( S \right) \right]$. This index captures the effect of feature$i$ with and without the presence of feature$j$. The main effect of feature $i$ is$\emptyset_{i, i}=\emptyset_{i}-\sum_{j\neq i} \emptyset_{i, j}$. Figures S3 (4-6) show the main effects for PTAU for the three classes after all interaction effects have been removed. As is seen, the feature has a clear and distinguishable role for every class. This role is not clear in Figure 5.

We can define different clusters for different types of values. However, dispersion of the same value (i.e. the same value can play different roles) is due to interactions. Figures 3 (7-9) show the interaction plots from PTAU with different features, according to the class. These features are selected automatically as the most significant features according to the SHAP values. For example, Figure 3 (7) shows the interaction between PTAU and ADNI_MEM with respect to the CN class. Interesting patterns can be defined. For instance, when TAU<20 and ADNI_MEM is HIGH, the SHAP values are positive, but PTAU>20 with HIGH ADNI_MEM values gives negative SHAP values. The low ADNI_MEM values have little interaction with PTAU. For example, PTAU>60 with low ADNI_MEM values give SHAP values in the range [0.0-0.005].


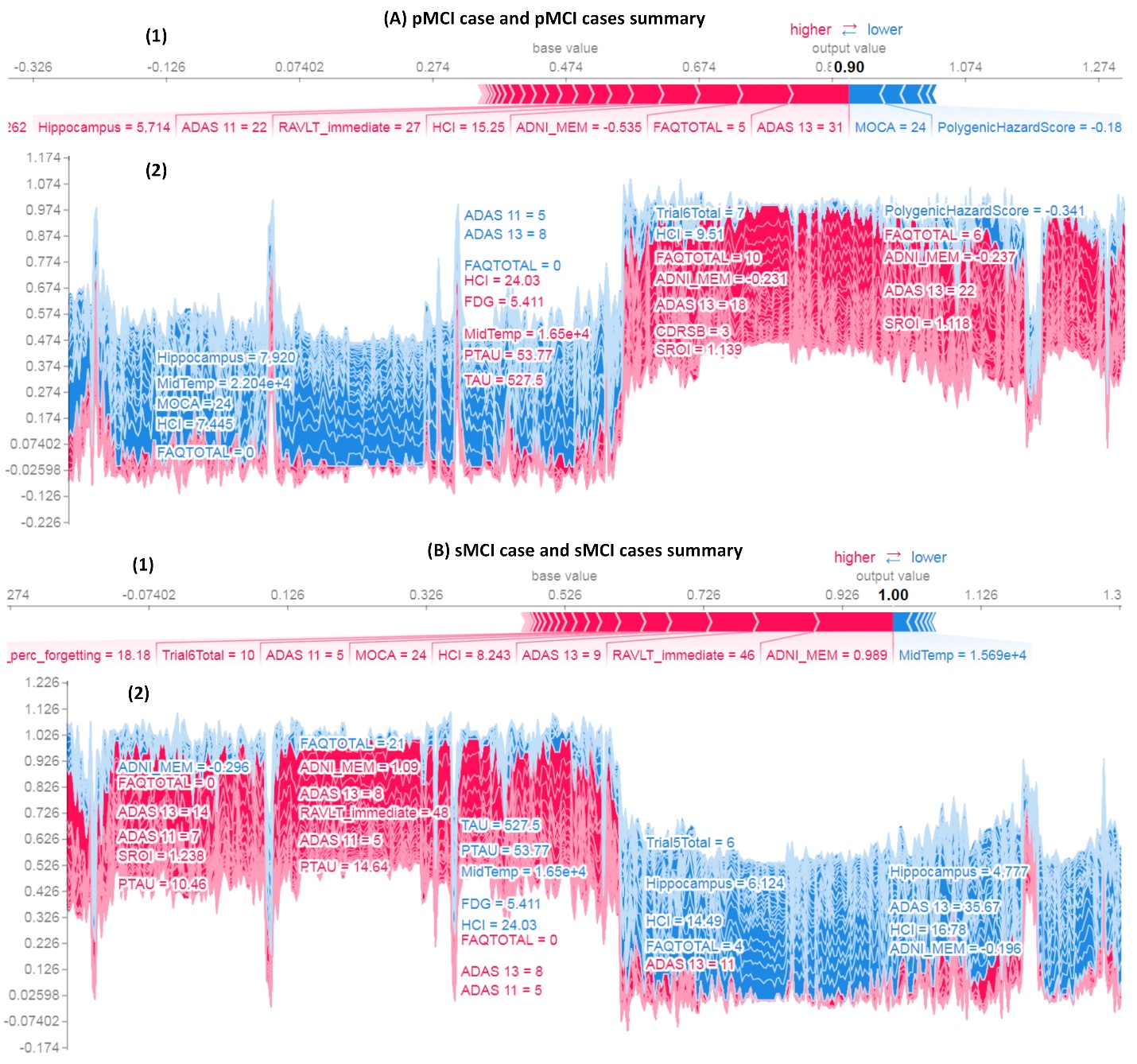


**Figure S2.** Sample prediction for two cases in the second layer: (A) pMCI class, and (B) sMCI class. In addition, SHAP supervised clustering is shown for system behavior in all cases of each class. Red feature attributions push the score higher, while blue feature contributions push the score lower. A few of the noticeable subgroups are annotated with the features that define them.

Figure S3 (8) illustrates the interaction between PTAU and CDRSB for the MCI class. As expected, the situation is mostly the inverse of the CN class. Low values of PTAU with low values of CDRSB have a negative effect towards the MCI class. There is little interaction between the two features when PTAU>20. Figure S3 (9) illustrates the interaction between PTAU and FAQTOTAL towards the AD class. There are many clear patterns from this interaction. Low values of PTAU (<30) with low values of FAQTOTAL have positive SHAP values, but low PTAU values with HIGH FAQTOTAL have negative SHAP values. In addition, PTAU>40 with low values for FAQTOTAL have negative SHAP values, and PTAU around 40 with HIGH values for FAQTOTAL have positive SHAP values. From the above analysis, the domain expert can understand the role of the PTAU feature in the classification process of the three classes. The same analysis can be done for all other features.


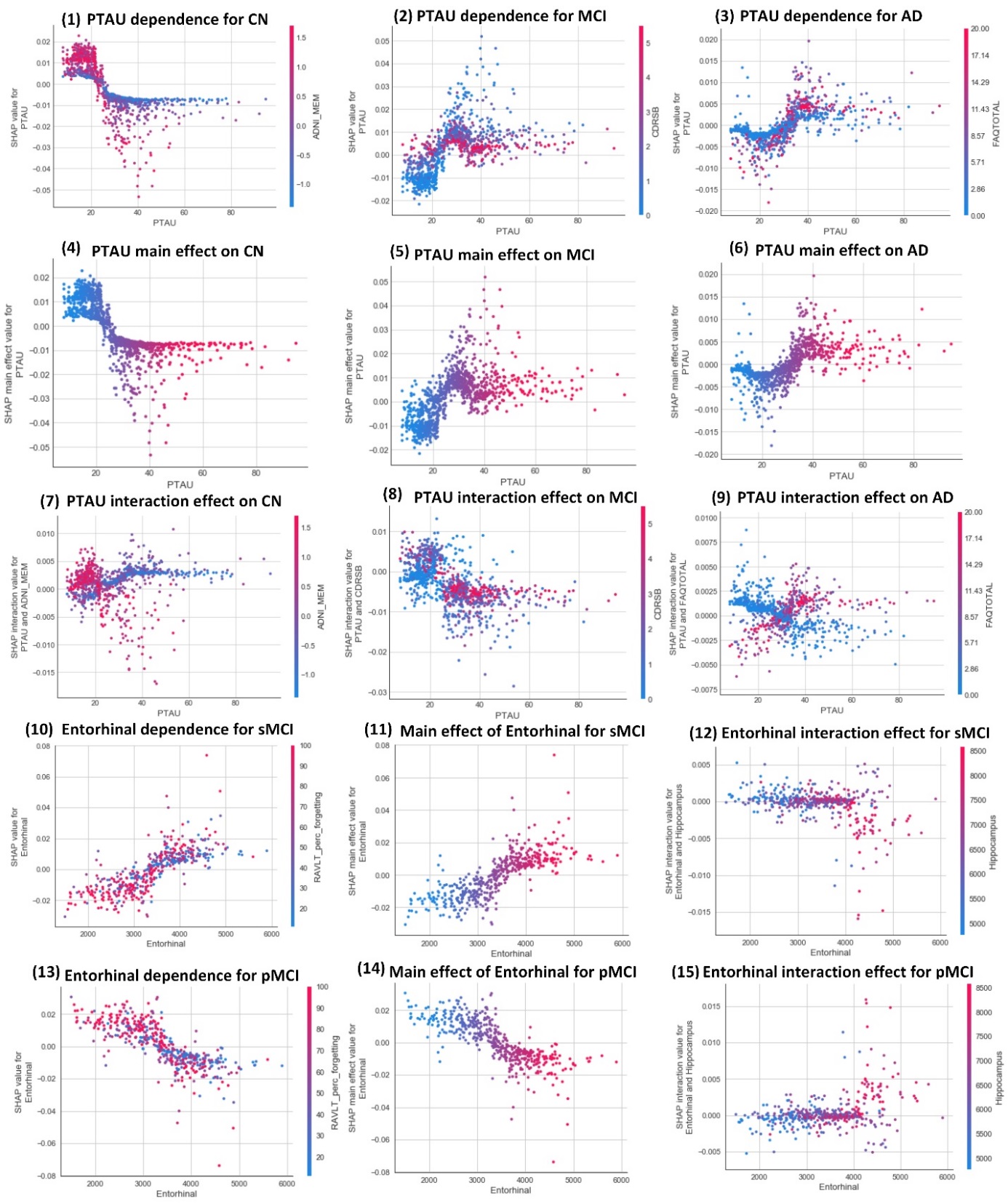


**Figure S3.** Feature interaction plots for PTAU from the first layer and for Entorhinal from the second layer.


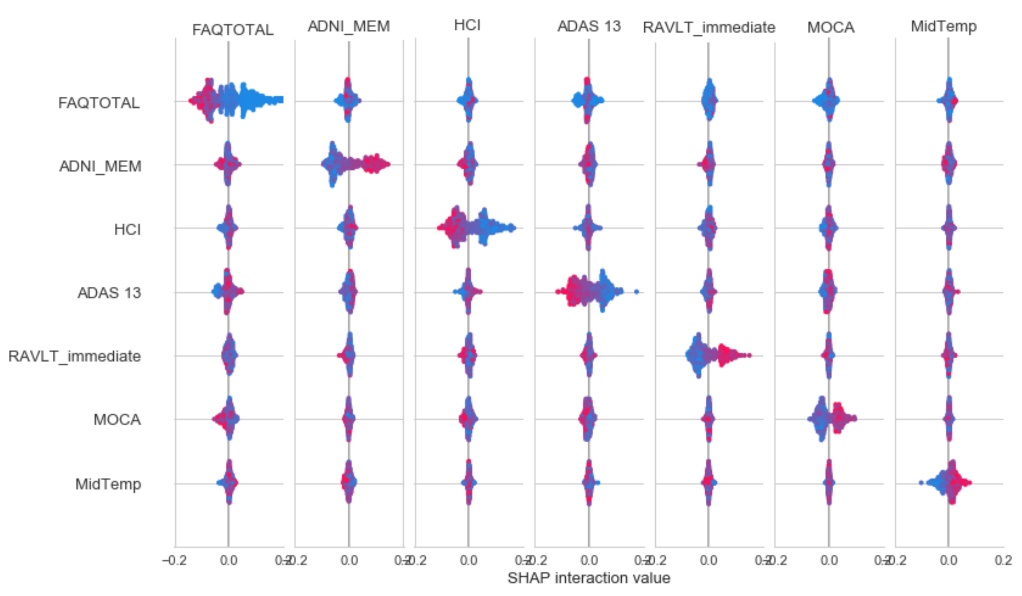


**Figure S4.** SHAP interaction summary plot of the most important features for the sMCI class in the second layer.


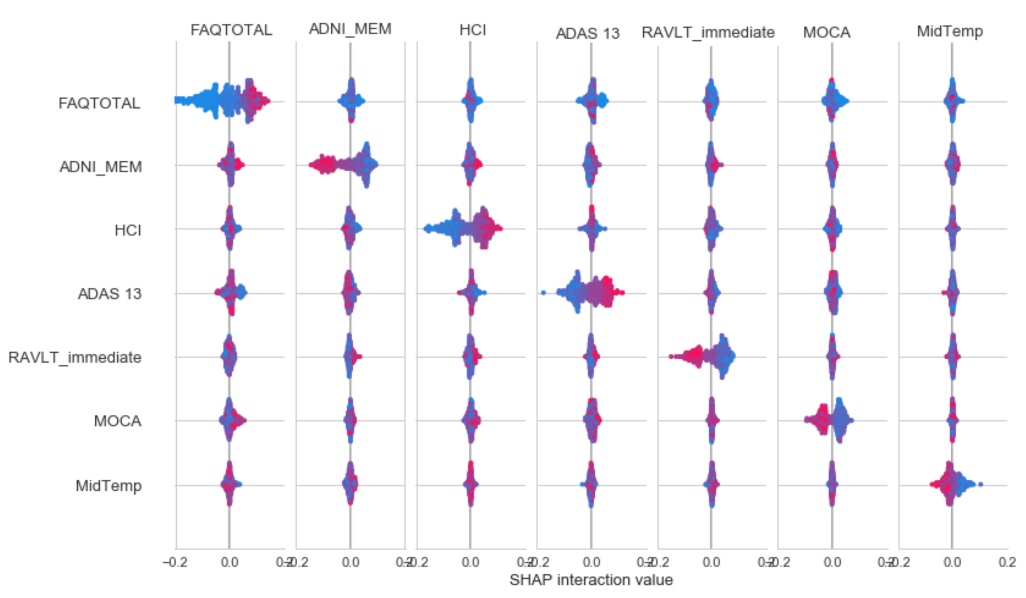


**Figure S5.** SHAP interaction summary plot of the most important features for the pMCI class in the second layer.


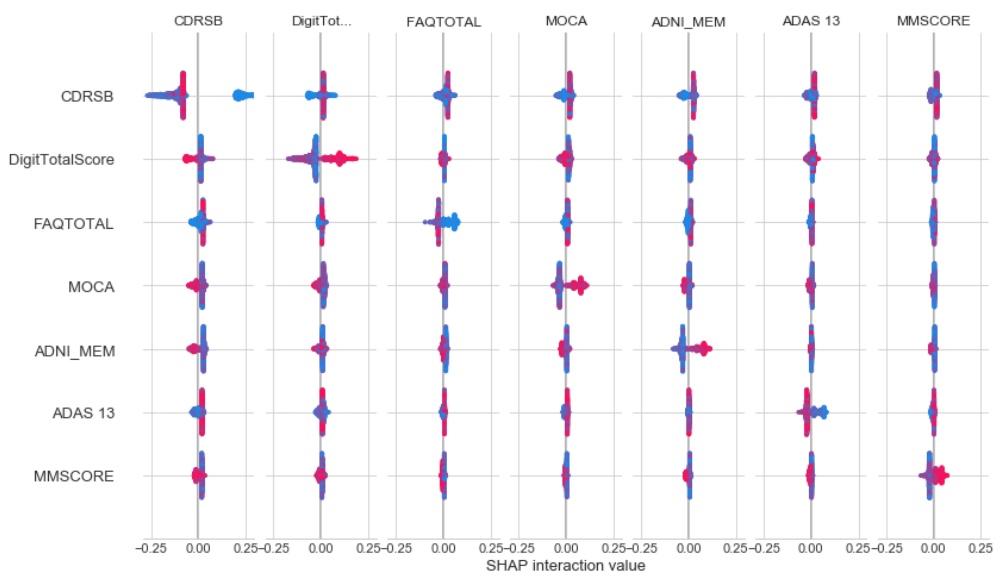


**Figure S6.** SHAP interaction summary plot of the most important features for the CN class in the first layer.


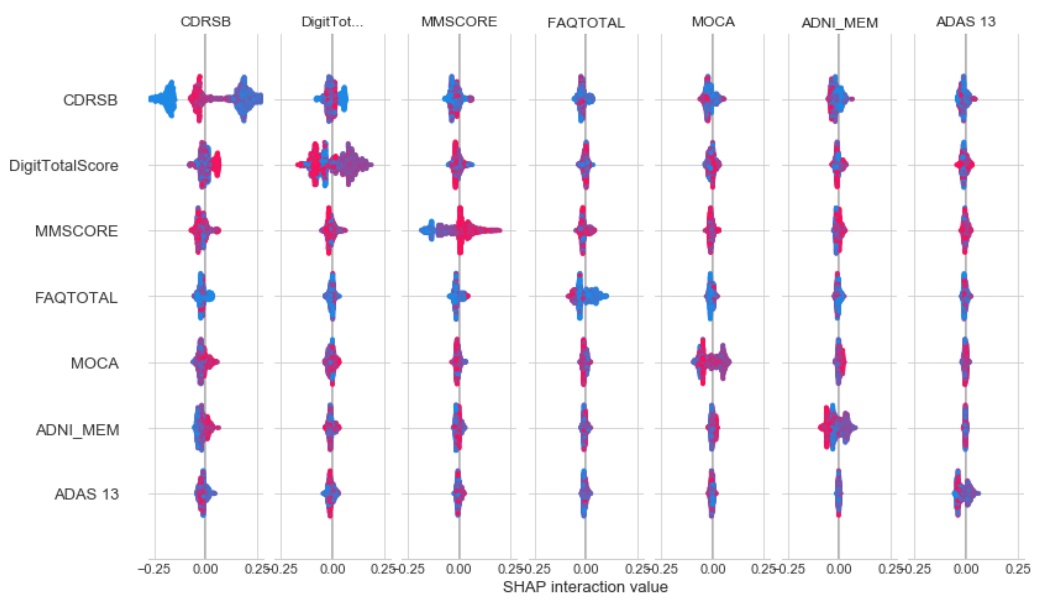


**Figure S7.** SHAP interaction summary plot of the most important features for the MCI class in the first layer.


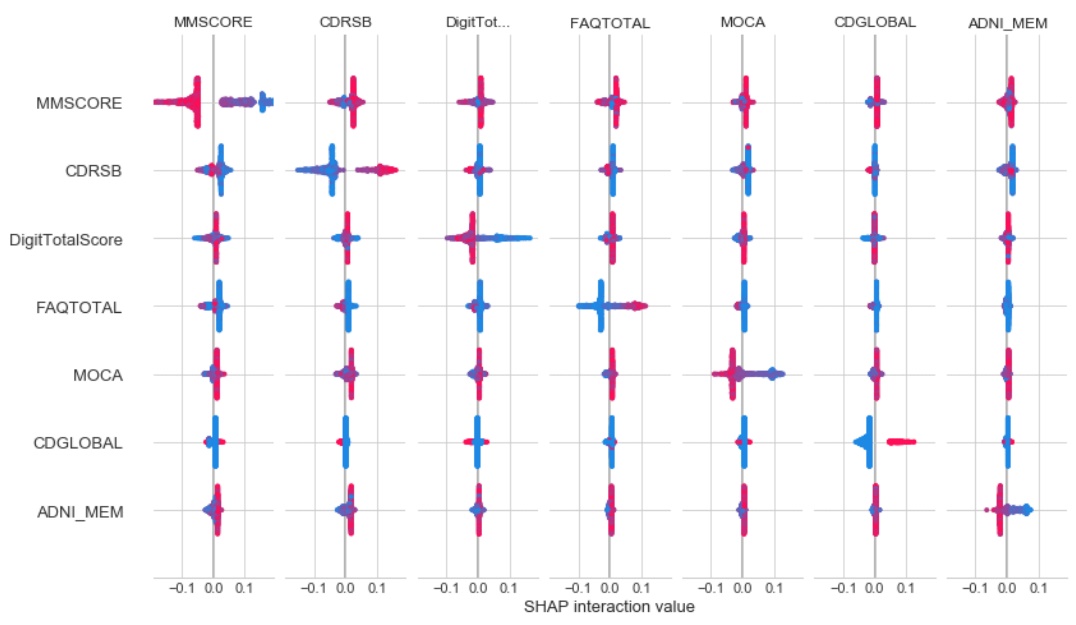


**Figure S8.** SHAP interaction summary plot of the most important features for the AD class in the first layer.

For the second layer, we select one feature (Entorhinal) that seems unimportant for the classifier. We need to prove that some features could be globally unimportant, but they could have critical roles for some groups of subjects. This depends on the types of interactions between features. Figure S3 (10) illustrates the dependency plot for Entorhinal and RAVLT percentage forgetting for the sMCI class. Entorhinal <3000 and HIGH values for RAVLT percentage forgetting have negative SHAP values. There are some outlier cases with Entorhinal <5000 and HIGH values of RAVLT percentage forgetting. These cases have the largest SHAP values. For these cases, Entorhinal may have the largest impact on the system decisions. Figure S3 (13) illustrates the dependence plot for Entorhinal and RAVLT percentage forgetting for the pMCI class. We can see that this figure is the inverse of Figure S3 (10), where Entorhinal <3000 and HIGH values of RAVLT percentage forgetting have high SHAP values, and the extreme points have very large, negative SHAP values. Figure S3 (11) illustrates the main impact of Entorhinal on the sMCI class. As can be seen, the small values of this feature have negative SHAP values, and the large values have a positive impact. Figure S3 (14) illustrates the main role of this feature for the pMCI class. The low values have a positive impact, and the high values have a negative impact. By studying the interaction between Entorhinal and Hippocampus, we can define the following patterns. For the sMCI class, low Hippocampus values with Entorhinal less than 3000 have positive SHAP values, but Entorhinal greater than 4000 with large Hippocampus values have less effect on the decisions. Lastly, Figure S3 (15) illustrates the interaction between Entorhinal and Hippocampus for the pMCI class. Again, it has a behavior inverse to the sMCI class shown in Figure S3 (12). Figures S4-S8 show the interaction plots for the most critical features in both layers and for every class.

**2.E NATURAL LANGUAGE–BASED EXPLANATIONS**

In this section, we provide some examples of natural-language explanations generated by the ExpliClas application programming interface (API). The explanations are based on decision tree (DT) and Fuzzy Unordered Rule Induction Algorithm (FURIA) fuzzy decisions. First-layer examples include three cases, (one each for CN, MCI, and AD classes). See tables T3-T5. Second-layer examples include two cases (one for sMCI and the other for pMCI), see tables T6 and T7. Some explanations and a comparison with SHAP are provided in the main text. We have evaluated the explainability capabilities for all instances in both datasets. In order to show the expressiveness of the generated explanations, let us concentrate now on some illustrative case studies, which are detailed in Supplementary files 2 and 3. We have randomly selected one specific data instance for each plausible output class to test the following: (1) the ability of explainers to generate supplementary explanations, (2) their consistency with the generated explanations from SHAP, and (3) the quality of the generated natural language explanations (Supplementary File 2). In the first layer, the selected instances have the following roster IDs (RID) with the output class in brackets: 5282 (CN), 2121 (MCI), and 753 (AD). In the second layer, the selected instances are 4849 (sMCI) and 4796 (pMCI).

**Case study 1**

In the case of instance 5282 (CN), the next four different modalities were considered: MRI, cognitive scores, lab tests, and neuropsychological battery. For example, the two explainers based on cognitive scores provide physicians with the same explanation “Diagnosis is CN because CDRSB is LOW.” Note that this explanation is in accordance with Figure 4 where, thanks to SHAP, we observed graphically how CDRSB turns up as one of the most informative attributes regarding CN. SHAP asserts that CDRSB=0 is the most critical factor that pushes RF towards selecting the CN class. In addition, DT based on MRI states that “Diagnosis is CN because Hippocampus is HIGH and Ventricles and ICV are LOW.” This is complementary to the explanation provided by FURIA based on MRI: “Diagnosis is CN because Entorhinal is HIGH and Whole_Brain_Volume is LOW.” Readers are referred to Supplementary files 2 and 3, where all explanations are detailed. We can see that these explanations add many values to the interpretability and confidence of the decisions made. First, the explainers could repeat and thus reinforce the same explanations from SHAP. Second, repeating the same decision from both explainers increases the confidence physicians have about the decision made. Third, explainers can consider different data not seen by the RF model. For example, for this CN case, both explainers gave justifications based on lab test features, MRI (ICV and Whole Brain Volume), and neuropsychological battery (Trial1Total), which were not considered by the RF model.

**Case study 2**

In instance 2121 (MCI), nine different modalities were considered for generating explanations. It has 13 complementary explanations (eight based on DT and five based on FURIA), which provide comprehensive analysis of the patient characteristics. Thus, explainers pay attention to all modalities except for PET and Genetics. For example, DT uses the patient’s medical history to classify him/her as MCI because the patient has an endocrine metabolic problem, and APOE4 is LOW. However, he/she is not AD, probably because he/she has no dermatologic connective tissue, hepatic, and renal genitourinary problems. Based on patient demographics, FURIA classifies the patient as MCI because AGE is LOW, there is a musculoskeletal problem, and Gender is Female. MRI is used by both explainers to provide complementary knowledge (i.e. ICV is HIGH, Ventricles is LOW, and Hippocampus is MEDIUM from DT; and MidTemp is HIGH from FURIA). Symptom modality is used by FURIA to indicate that the patient has no urinary frequency problems, but he/she had constipation. By symptoms modality, DT induces that the patient may be not AD because he is not crying, has no chest pain, does not suffer from depression, is not vomiting, and no wandering. As it can be seen, the two explainers could depend on different features from the same modality. Physicians can investigate all the information to understand why the system makes a specific decision. We note perfect matches among SHAP and explainers’ outputs. For example, FURIA classifies this case as MCI because “FAQ is LOW.” From figure 2, it is clear that low values of FAQ are highly related to the MCI class. DT supports MCI based on lab test as HMT16_lymphocytespercentage is HIGH; HMT11_eosinophils, HMT15_neutrophilspercentage, RCT20_cholesterol, RCT5_AST_SGOT, RCT9_phosphorus and RedBloodCellCount are LOW; and HMT12_basophils is MEDIUM.

**Case study 3**

In instance 753 (AD), eight different modalities (all except for lab tests, neurological exams and physical exams) were considered for generating explanations. The case has 11 explanations (four from FURIA and seven from DT). Based on MRI and PET, FURIA gives the following explanations: Entorhinal is LOW, Hippocampus is LOW, and FDG is LOW, and HCI is MEDIUM. DT adds “SROI is LOW” as a possible reason for predicting AD class. Genetics modality is considered by both explainers. DT concludes the main reasons for selecting the AD class are that ABETA is LOW, PolygenicHazardScore is MEDIUM, and TAU is MEDIUM. DT points out the selected ABETA value (LOW) by FURIA, but it selects another value for PolygenicHazardScore (HIGH) and adds another cause: PTAU is MEDIUM. We can conclude that AD patients cannot have low values for Polygenichazardscore. Based on the neuropsychological battery modality, DT supports the AD class because DigitTotalScore, Trial3Total, and Trial6Total are LOW; FURIA adds that Trabscor_PartBTimeToComplete is HIGH, ListBTotal is LOW, and Trial2Total is MEDIUM can indicate the AD class. Based on patient history, DT supports indicating the AD class because the patient is crying and suffers depression. This information is fully compatible with SHAP explanations. Many more explanations can be seen in the Supplementary File 2. Of course, all these explanations are complementary, and support the output class pointed out by the RF model. Due to space restrictions, the explanations from the Second Layer can be found in the Supplementary File 2. In summary, we observed how explanations related to sMCI and pMCI are somehow in contrast (and in accordance with) physicians’ intuition and background. In addition, the provided explanations are fully compatible with the SHAP decisions.

The classification of instance 4849 (sMCI) is explained in terms of all 11 of the modalities. In addition, the explanations related to instance 4796 (pMCI) are provided by the DT (regarding all modalities except for neurological exams, physical exams, and vital signs) and by the FURIA, which takes into account PET, MRI, cognitive scores, neuropsychological battery, neurological exams, and symptoms. We observe how explanations related to sMCI and pMCI are somehow in contrast, but in accordance, with physicians’ intuition and background. For example, DT (Genetics) says “Diagnosis is sMCI because ABETA is HIGH” (regarding instance 4849) and “Diagnosis is pMCI because ABETA and PolygenicHazardScore are LOW and PTAU is MEDIUM” (regarding instance 4796).

| **Table T3**. Case 1 explainers |
| --- |
| **RID:** 5282. **Diagnosis:** CN |
| **Patient characteristics:** Supplementary File 3 |
| **Decision tree** explanations based on MRI, Cognitive Scores, Lab Tests, and Neuropsychological Battery modalities   1. *Based on MRI dataset:*   DX is CN because Hippocampus is **HIGH** and Ventricles and ICV are **LOW**.   1. *Based on Cognitive Score dataset:*   DX is CN because CDRSB is **LOW**.   1. *Based on Lab Tests dataset:*   DX is CN because HMT100_MCH is **HIGH**, HMT11_eosinophils, HMT15_neutrophilspercentage, HMT16_lymphocytespercentage, and RCT29_directbilirubin are **LOW**, and HMT3_RBC and RCT5_AST_SGOT are **MEDIUM**.   1. *Based on Neuropsychological Battery dataset:*   DX is CN because DigitTotalScore and RAVLT_immediate are **HIGH**, and  Trial6Total is **MEDIUM**.  **FURIA** explanations based on MRI, Cognitive Scores, Lab Tests, and Neuropsychological Battery modalities   1. *Based on MRI dataset:*   DX is CN because Entorhinal is **HIGH** and Whole_Brain_Volume is **LOW**.   1. *Based on Cognitive Scores dataset:*   DX is CN because CDRSB is **LOW**.   1. *Based on Lab Tests dataset:*   DX is CN because HMT40_hemoglobin is **HIGH**, RCT13_albumin is **LOW**, and RCT11_serumglucose is **MEDIUM**.   1. *Based on Neuropsychological Battery dataset:*   DX is CN because DigitTotalScore is **HIGH**, and Trial1Total is **MEDIUM**. |

| **Table T4**. Case 2 explainers |
| --- |
| **RID:** 2121. **Diagnosis:** MCI |
| **Patient characteristics:** Supplementary File 3 |
| **Decision tree** explanations based on MRI, Medical History, Lab Tests, Neuropsychological Battery, Neurological Exams, Physical Exams, Symptoms, and Vital Signs modalities   1. *Based on MRI dataset:*   DX is MCI because ICV is **HIGH**, Ventricles is **LOW**, and Hippocampus is **MEDIUM**.   1. *Based on Medical History dataset:*   DX is MCI because HasDermatologicConnectiveTissueProblem, HasHepaticProblem, and HasRenalGenitourinaryProblem are **NO**, APOE4 is **LOW**, and HasEndocrineMetabolicProblem is **YES**.   1. *Based on Lab Tests dataset:*   DX is MCI because HMT16_lymphocytespercentage is **HIGH**, HMT11_eosinophils, HMT15_neutrophilspercentage, RCT20_cholesterol, RCT5_AST_SGOT, RCT9_phosphorus, and RedBloodCellCount are **LOW**, and HMT12_basophils is **MEDIUM**.   1. *Based on Neuropsychological Battery dataset:*   DX is MCI because Trial5Total is **HIGH**, Traberrom_ErrorsOfOmission is **LOW**, and DigitTotalScore and RAVLT_Forgetting are **MEDIUM**.   1. *Based on Neurological Exams dataset:*   DX is MCI because HasCerebellarFingerToNose, HasGait, HasLevelOfConsciousness, and HasSignificantVisualImpairment are **NO**.   1. *Based on Physical Exams dataset:*   DX is MCI because ChestProblem, GeneralAppearanceProblem, MusculoskeletalProblem, and PeripheralVascularProblem are **NO**.   1. *Based on Symptoms dataset:*   DX is MCI because ChestPain, Crying, DepressedMood, Palpitations, Rash, Vomiting, and Wandering are **NO**.   1. *Based on Vital Signs dataset:*   DX is MCI because Height is **HIGH**, BMI, RespirationsPerMinute, and SeatedPulseRatePerMinute are **LOW**, and Temperature is **MEDIUM**.  **FURIA** explanations based on MRI, Cognitive Scores, Medical History, Neuropsychological Battery, and Symptoms modalities   1. *Based on MRI dataset:*   DX is MCI because MidTemp is **HIGH**, and Hippocampus is **MEDIUM**.   1. *Based on Cognitive Scores dataset:*   DX is MCI because FAQTOTAL is **LOW**.   1. *Based on Medical History dataset:*   DX is MCI because AGE is **LOW**, HasMusculoskeletalProblem is **YES**, and Gender is **FEMALE**.   1. *Based on Neuropsychological Battery dataset:*   DX is MCI because DigitTotalScore is **MEDIUM**.   1. *Based on Symptoms dataset:*   DX is MCI because UrinaryFrequencyProblem is **NO**, and Constipation is **YES**. |
| **Table T5**. Case 3 explainers |
| **RID:** 753. **Diagnosis:** AD |
| **Patient characteristics:** Supplementary File 3 |
| **Decision tree** explanations based on PET, MRI, Genetics, Medical History, Neuropsychological Battery, and Symptoms modalities   1. *Based on MRI dataset:*   DX is AD because Entorhinal and Hippocampus are **LOW**.   1. *Based on Medical History dataset:*   DX is AD because HasHepaticProblem is **NO**, APOE4 is **LOW** and HasDermatologicConnectiveTissueProblem and HasPsychiatric are **YES**.   1. *Based on Cognitive Scores dataset:*   DX is AD because CDGLOBAL is **LOW**, and CDRSB is **MEDIUM**.   1. *Based on PET dataset:*   DX is AD because HCI is **HIGH** and FDG and SROI are **LOW**.   1. *Based on Neuropsychological Battery dataset:*   DX is AD because DigitTotalScore, Trial3Total, and Trial6Total are **LOW**.   1. *Based on Genetics dataset:*   DX is AD because ABETA is **LOW** and PolygenicHazardScore and TAU are **MEDIUM**.   1. *Based on Symptoms dataset:*   DX is AD because AnkleSwelling and Wandering are **NO** and Crying and DepressedMood are **YES**.  **FURIA** explanations based on PET, Cognitive Scores, Genetics, and Neuropsychological Battery modalities   1. *Based on PET dataset:*   DX is AD because FDG is **LOW** and HCI is **MEDIUM**.   1. *Based on Cognitive scores dataset:*   DX is AD because CDGLOBAL is **LOW**, and CDRSB is **MEDIUM**.   1. *Based on Genetics dataset:*   DX is AD because PolygenicHazardScore is **HIGH**, ABETA is **LOW**, and PTAU is **MEDIUM**.   1. *Based on Neuropsychological Battery dataset:*   DX is AD because Trabscor_PartBTimeToComplete is **HIGH**, ListBTotal is **LOW**, and Trial2Total is **MEDIUM**. |

| **Table T6**. Case 4 explainers |
| --- |
| **RID:** 4849. **Diagnosis:** sMCI |
| **Patient characteristics:** Supplementary File 3 |
| **Decision tree** explanations based on Cognitive Scores, Genetics, Medical History, Lab Tests, Neuropsychological Battery, Neurological Exam, Physical Exams, and Vital Signs modalities   1. *Based on Cognitive Scores dataset:*   DX is sMCI because ADNI_MEM is **HIGH**.   1. *Based on Medical History dataset:*   DX is sMCI because APOE4 is **LOW** and DidMajorSurgicalProcedures and FatherHadAlzheimer are **YES**.   1. *Based on Genetics dataset:*   DX is sMCI because ABETA is **HIGH**.   1. *Based on Lab Tests dataset:*   DX is sMCI because GlucoseResults is **HIGH**, HMT19_basophilspercentage, RCT1408_ldh, RCT183_calcium_edta, RCT3_GGT, and RCT8_serumuricacid are **LOW**, and HMT8_neutrophils, HMT9_lymphocytes, and RedBloodCellCount are **MEDIUM**.   1. *Based on Neuropsychological Battery dataset:*   DX is sMCI because RAVLT_immediate is **HIGH** and RAVLT_forgetting is **LOW**.   1. *Based on Neurological Exams dataset:*   DX is sMCI because HasCerebellarFingerTonose, HasCranialNervesProblem, HasGait and HasPlantarReflexesProblem are **NO**.   1. *Based on Physical Exams dataset:*   DX is sMCI because GeneralAppearanceProblem and PeripheralVascularProblem are **NO** and AbdomenProblem is **YES**.   1. *Based on Vital Signs dataset:*   DX is sMCI because Weight is **MEDIUM**.  **FURIA** explanations based on MRI, Medical History, Neurological Exams, Cognitive Scores, Neuropsychological Battery, Genetics, and Physical Exams modalities   1. *Based on MRI dataset:*   DX is sMCI because Hippocampus is **HIGH** and ICV is **LOW**.   1. *Based on Medical History dataset:*   DX is sMCI because APOE4 is **LOW** and FatherHadAlzheimer is **YES**.   1. *Based on Physical Exams dataset:*   *DX is sMCI because HeadEyesEarsNoseAndThroatProblem is* ***NO*** *and AbdomenProblem is* ***YES****.*   1. *Based on Cognitive Scores dataset:*   DX is sMCI because ADNI_MEM is **HIGH**, and ADNI_EF is **MEDIUM**.   1. *Based on Genetics dataset:*   DX is sMCI because PTAU is **LOW,** and ABETA is **MEDIUM**.   1. *Based on Neuropsychological Battery dataset:*   DX is sMCI because RAVLT_immediate is **HIGH,** and RAVLT_forgetting is **LOW**.   1. *Based on Neurological Exams dataset:*   DX is sMCI because HasSignificantAuditoryImpairment is **NO**. |

| **Table T7**. Case 5 explainers |
| --- |
| **RID:** 4796. **Diagnosis:** pMCI |
| **Patient characteristics:** Supplementary File 3 |
| **Decision tree** explanations based on PET, MRI, Cognitive Scores, Genetics, Medical History, Lab Tests, Neuropsychological Battery, and Symptoms modalities   1. *Based on Cognitive Scores dataset:*   DX is pMCI because ADNI_MEM, FAQTOTAL, and MOCA are **LOW**, and ADAS 11 is **MEDIUM**.   1. *Based on PET dataset:*   DX is pMCI because FDG is **LOW,** and HCI is **MEDIUM**.   1. *Based on Genetics dataset:*   DX is pMCI because ABETA and PolygenicHazardScore are **LOW,** and PTAU is **MEDIUM**.   1. *Based on Lab Tests dataset:*   DX is pMCI because HMT17_monocytespercentage, HMT18_eosinophilspercentage, HMT9_lymphocytes, RCT13_albumin, RCT183_calcium_edta, and RCT3_GGT are **LOW**, and HMT3_RBC, HMT8_neutrophils, and RedBloodCellCount are **MEDIUM**.   1. *Based on Neuropsychological Battery dataset:*   DX is pMCI because Trial5Total is **HIGH**, CategoryFluencyAnimalsTotalCorrect, Clock_TotalScore and RAVLT_immediate are **LOW**, Clock_ApproximatelyCircularFace and Copy_ApproximatelyCircularFace are **YES,** and Trial2Total is **MEDIUM**.   1. *Based on Medical History dataset:*   DX is pMCI because FatherHadAlzheimer, HasHematopoieticLymphaticProblem and HasNeurologicOtherThanAlzheimer are **NO**, APOE4 is **LOW**, Marry_status is MARRIED and HasCardiovascularProblem, HasMalignancy, and MotherHadDementia are **YES**.   1. *Based on MRI dataset:*   DX is pMCI because Hippocampus is **LOW**.   1. *Based on Symptoms dataset:*   DX is pMCI because crying, DepressedMood, ElevatedMood, Headache, Insomnia, and Rash are **NO,** and Diarrhea is **YES**.  **FURIA** explanations based on PET, MRI, Cognitive Scores, Neuropsychological Battery, and Symptoms modalities   1. *Based on Cognitive Scores dataset:*   DX is pMCI because ADAS 13 is **HIGH,** and ADNI_EF is **LOW**.   1. *Based on PET dataset:*   DX is pMCI because FDG is **LOW**.   1. *Based on Neuropsychological Battery dataset:*   DX is pMCI because RecognitionScore is **HIGH,** and ListBTotal is **LOW**.   1. *Based on MRI dataset:*   DX is pMCI because Fusiform is **LOW**.   1. *Based on Symptoms dataset:*   DX is pMCI because Coughing is **NO**. |

**PART 3: CASE STUDIES DESCRIPTION**

**1. Case study 1: CN**

| **MRI** | | | | | | | | |  | | | | | | | | | | | | | | | | | | | | | | | | | | | | | | | | | |
| --- | --- | --- | --- | --- | --- | --- | --- | --- | --- | --- | --- | --- | --- | --- | --- | --- | --- | --- | --- | --- | --- | --- | --- | --- | --- | --- | --- | --- | --- | --- | --- | --- | --- | --- | --- | --- | --- | --- | --- | --- | --- | --- |
| **RID** | **Entorhinal** | **Fusiform** | **Hippocampus** | **MidTemp** | **Ventricles** | **Volume of Icv** | **Whole_brain_volume** | **DX** |  |  |  |  |  |  |  |  |  |  |  |  |  |  |  |  |  |  |  |  |  |  |  |  |  |  |  |  |  |  |  |  |  |  |
| 5282 | 4393 | 19227 | 7851 | 20552 | 39208 | 1498720 | 1051525 | CN |  |  |  |  |  |  |  |  |  |  |  |  |  |  |  |  |  |  |  |  |  |  |  |  |  |  |  |  |  |  |  |  |  |  |
| **Cognitive Scores** | | | | | | | | | | | | | | | | | | | | | | | | | | | | | | | | | | | | | | | | | | |
| **RID** | **ADAS 11** | **ADAS 13** | **ADNI_EF** | **ADNI_MEM** | **CDGLOBAL** | **CDRSB** | **FAQTOTAL** | **GDTOTAL** | **HMSCORE** | **MMSCORE** | **MOCA** | **NPISCORE** | **DX** |  |  |  |  |  |  |  |  |  |  |  |  |  |  |  |  |  |  |  |  |  |  |  |  |  |  |  |  |  |
| 5282.0 | 10.0 | 15.0 | 1.2 | .9 | .0 | .0 | .0 | 1.0 | .0 | 29.0 | 25.3 | .0 | CN |  |  |  |  |  |  |  |  |  |  |  |  |  |  |  |  |  |  |  |  |  |  |  |  |  |  |  |  |  |
| **Lab tests** | | | | | | | | | | | | | | | | | | | | | | | | | | | | | | | | | | | | | | | | | | |
| **RID** | **BAT126_VitaminB12** | **RedBloodCellCount** | **WhiteBloodCellCount** | **GlucoseResults** | **HMT2_Hematocrit** | **HMT3_RBC** | **HMT4_MCV** | **HMT7_WBC** | **HMT8_Neutrophils** | **HMT9_Lymphocytes** | **HMT10_Monocytes** | **HMT11_Eosinophils** | **HMT12_Basophils** | **HMT13_Platelets** | **HMT15_NeutrophilsPercentage** | **HMT16_LymphocytesPercentage** | **HMT17_MonocytesPercentage** | **HMT18_EosinophilsPercentage** | **HMT19_BasophilsPercentage** | **HMT40_Hemoglobin** | **HMT100_MCH** | **HMT102_MCHC** | **ProteinResults** | **RCT1_TotalBilirubin** | **RCT3_GGT** | **RCT4_ALT_SGPT** | **RCT5_AST_SGOT** | **RCT6_UreaNitrogen** | **RCT8_SerumUricAcid** | **RCT9_Phosphorus** | **RCT11_SerumGlucose** | **RCT12_TotalProtein** | **RCT13_Albumin** | **RCT14_CreatineKinase** | **RCT19_Triglycerides_GPO** | **RCT20_Cholesterol** | **RCT29_DirectBilirubin** | **RCT183_Calcium_EDTA** | **RCT392_Creatinine** | **RCT1407_AlkalinePhosphatase** | **RCT1408_LDH** | **DX** |
| 5282.0 | 508.5 | .0 | .0 | 70.0 | 40.6 | 4.7 | 86.0 | 5.2 | 3.2 | 1.4 | .4 | .2 | .0 | 277.1 | 60.5 | 27.8 | 7.3 | 3.4 | .9 | 14.2 | 29.8 | 34.8 | 47.0 | .4 | 21.0 | 21.4 | 22.6 | 19.0 | 4.6 | 3.7 | 99.7 | 7.1 | 4.1 | 110.2 | 149.0 | 201.0 | .1 | 9.7 | .9 | 81.0 | 176.0 | CN |
| **Neuropsychological Battery** | | | | | | | | | | | | | | | | | | | | | | | | | | | | | | | | | | | | | | | | | | |
| **RID** | **A30MinuteDelayTotal** | **RecognitionScore** | **Trial1Total** | **Trial2Total** | **Trial3Total** | **Trial4Total** | **Trial5Total** | **Trial6Total** | **ListBTotal** | **CategoryFluencyAnimalsTotalCorrect** | **CategoryFluencyAnimalsIntrusions** | **CategoryFluencyAnimalsPerseverations** | **Clock_ApproximatelyCircularFace** | **Clock_PresenceOfTheTwoHands** | **Clock_CorrectnessOfNumbers** | **Clock_TotalScore** | **Clock_SymmetryOfNumberPlacement** | **Clock_PresenceOfTheTwoHandsSetToTenAfterEleven** | **Copy_ApproximatelyCircularFace** | **Copy_PresenceOfTheTwoHands** | **Copy_CorrectnessOfNumbers** | **Copy_TotalScore** | **Copy_SymmetryOfNumberPlacement** | **Copy_PresenceOfTheTwoHandsSetToTenAfterEleven** | **DigitTotalScore** | **RAVLT_forgetting** | **RAVLT_immediate** | **RAVLT_learning** | **RAVLT_perc_forgetting** | **ErrorsOfCommission** | **ErrorsOfOmission** | **TRAASCOR_PartATimeToComplete** | **TRABERRCOM_ErrorsOfCommission** | **TRABERROM_ErrorsOfOmission** | **TRABSCOR_PartBTimeToComplete** | **DX** |  |  |  |  |  |  |
| 5282.0 | 8.0 | 14.0 | 5.0 | 7.0 | 9.0 | 11.0 | 10.0 | 9.0 | 4.0 | 15.0 | .0 | .0 | YES | YES | YES | 5.0 | YES | YES | YES | YES | YES | 5.0 | YES | YES | 50.0 | 2.0 | 42.0 | 5.0 | 20.0 | .0 | .0 | 28.0 | .0 | .0 | 57.0 | CN |  |  |  |  |  |  |

**2. Case study 2: MCI**

| **Medical history** | | | | | | | | | | | | | | | | | | | | | | | | | | | | | | | | | | | | | | | | | | | | | | | | | | | | | | | | | | | | | | | | | | | | | | |
| --- | --- | --- | --- | --- | --- | --- | --- | --- | --- | --- | --- | --- | --- | --- | --- | --- | --- | --- | --- | --- | --- | --- | --- | --- | --- | --- | --- | --- | --- | --- | --- | --- | --- | --- | --- | --- | --- | --- | --- | --- | --- | --- | --- | --- | --- | --- | --- | --- | --- | --- | --- | --- | --- | --- | --- | --- | --- | --- | --- | --- | --- | --- | --- | --- | --- | --- | --- | --- | --- | --- |
| **RID** | | **FatherHadDementia** | | **FatherHadAlzheimer** | | **MotherHadDementia** | | **MotherHadAlzheimer** | | **HasNeurologicOtherThanAlzheimer** | | **HasHeadEyesEarsNoseAndThroatProblem** | | **HasCardiovascularProblem** | | **HasRespiratoryProblem** | | **HasHepaticProblem** | | | **HasDermatologicConnectiveTissueProblem** | | **HasMusculoskeletalProblem** | | **HasEndocrineMetabolicProblem** | | **HasGastrointestinalProblem** | | **HasHematopoieticLymphaticProblem** | | **HasRenalGenitourinaryProblem** | | **HasAllergiesOrDrugSensitivities** | | **IsAlcoholAbuse** | | **IsDrugAbuse** | | **IsSmoker** | | **HasMalignancy** | | **DidMajorSurgicalProcedures** | | **HasPsychiatric** | | **AGE** | | **APOE4** | | **Marry_Status** | | **Gender** | | | **NumberOfYearsEducation** | | **DX** | |  |  |  |  |  |  |  |  |  |  |  |
| 2121 | | NO | | NO | | YES | | NO | | NO | | NO | | NO | | NO | | NO | | | NO | | YES | | YES | | NO | | NO | | NO | | YES | | NO | | NO | | YES | | NO | | YES | | NO | | 67.8 | | 1.0 | | Divorced | | Female | | | 14.0 | | MCI | |  |  |  |  |  |  |  |  |  |  |  |
| **Lab tests** | | | | | | | | | | | | | | | | | | | | | | | | | | | | | | | | | | | | | | | | | | | | | | | | | | | | | | | | | | | | | | | | | | | | | | |
| **RID** | **BAT126_VitaminB12** | | **RedBloodCellCount** | | **WhiteBloodCellCount** | | **GlucoseResults** | | **HMT2_Hematocrit** | | **HMT3_RBC** | | **HMT4_MCV** | | **HMT7_WBC** | | **HMT8_Neutrophils** | | **HMT9_Lymphocytes** | **HMT10_Monocytes** | | **HMT11_Eosinophils** | | **HMT12_Basophils** | | **HMT13_Platelets** | | **HMT15_NeutrophilsPercentage** | | **HMT16_LymphocytesPercentage** | | **HMT17_MonocytesPercentage** | | **HMT18_EosinophilsPercentage** | | **HMT19_BasophilsPercentage** | **HMT40_Hemoglobin** | **HMT100_MCH** | | **HMT102_MCHC** | | **ProteinResults** | | **RCT1_TotalBilirubin** | | **RCT3_GGT** | | **RCT4_ALT_SGPT** | | **RCT5_AST_SGOT** | | **RCT6_UreaNitrogen** | | **RCT8_SerumUricAcid** | **RCT9_Phosphorus** | | **RCT11_SerumGlucose** | | **RCT12_TotalProtein** | | **RCT13_Albumin** | **RCT14_CreatineKinase** | **RCT19_Triglycerides_GPO** | **RCT20_Cholesterol** | **RCT29_DirectBilirubin** | **RCT183_Calcium_EDTA** | **RCT392_Creatinine** | **RCT1407_AlkalinePhosphatase** | **RCT1408_LDH** | **DX** |
| 2121 | 408.0 | | 66.0 | | .0 | | 57.0 | | 41.0 | | 4.7 | | 87.0 | | 6.2 | | 3.3 | | 2.5 | .3 | | .1 | | .0 | | 279.0 | | 53.5 | | 40.0 | | 4.1 | | 1.8 | | .6 | 13.7 | 29.0 | | 34.0 | | 30.0 | | .2 | | 12.0 | | 11.0 | | 15.0 | | 12.0 | | 4.7 | 4.0 | | 92.0 | | 6.6 | | 4.1 | 60.0 | 156.0 | 225.0 | .1 | 9.6 | .8 | 80.0 | 171.0 | MCI |
| **Neuropsychological battery** | | | | | | | | | | | | | | | | | | | | | | | | | | | | | | | | | | | | | | | | | | | | | | | | | | | | | | | | | | | | | | | | | | | | | | |
| **RID** | **A30MinuteDelayTotal** | | **RecognitionScore** | | **Trial1Total** | | **Trial2Total** | | **Trial3Total** | | **Trial4Total** | | **Trial5Total** | | **Trial6Total** | | **ListBTotal** | | **CategoryFluencyAnimalsTotalCorrect** | **CategoryFluencyAnimalsIntrusions** | | **CategoryFluencyAnimalsPerseverations** | | **Clock_ApproximatelyCircularFace** | | **Clock_PresenceOfTheTwoHands** | | **Clock_CorrectnessOfNumbers** | | **Clock_TotalScore** | | **Clock_SymmetryOfNumberPlacement** | | **Clock_PresenceOfTheTwoHandsSetToTenAfterEleven** | | **Copy_ApproximatelyCircularFace** | **Copy_PresenceOfTheTwoHands** | **Copy_CorrectnessOfNumbers** | | **Copy_TotalScore** | | **Copy_SymmetryOfNumberPlacement** | | **Copy_PresenceOfTheTwoHandsSetToTenAfterEleven** | | **DigitTotalScore** | | **RAVLT_forgetting** | | **RAVLT_immediate** | | **RAVLT_learning** | | **RAVLT_perc_forgetting** | **ErrorsOfCommission** | | **ErrorsOfOmission** | | **TRAASCOR_PartATimeToComplete** | | **TRABERRCOM_ErrorsOfCommission** | **TRABERROM_ErrorsOfOmission** | **TRABSCOR_PartBTimeToComplete** | **DX** |  |  |  |  |  |  |
| 2121 | 6.0 | | 9.0 | | 3.0 | | 9.0 | | 9.0 | | 11.0 | | 9.0 | | 6.0 | | 6.0 | | 18.0 | .0 | | 1.0 | | YES | | YES | | YES | | 5.0 | | YES | | YES | | YES | YES | YES | | 5.0 | | YES | | YES | | 40.0 | | 3.0 | | 41.0 | | 6.0 | | 33.3 | 1.0 | | .0 | | 60.0 | | .0 | .0 | 120.0 | MCI |  |  |  |  |  |  |
| **Neurological exams** | | | | | | | | | | | | | | | | | | | | | | | | | | | | | | | | | | | | | | | | | | | | | | | | | | | | | | | | | | | | | | | | | | | | | | |
| **RID** | **HasSignificantAuditoryImpairment** | | **HasLevelofConsciousness** | | **HasCerebellarFingerToNose** | | **HasGait** | | **HasCerebellarHeelToShin** | | **HasMotorStrengthProblem** | | **HasCranialNervesProblem** | | **HasPlantarReflexesProblem** | | **HasSensoryProblem** | | **HasDeepTendonReflexesProblem** | **HasTremorProblem** | | **HasSignificantVisualImpairment** | | **DX** | |  |  |  |  |  |  |  |  |  |  |  |  |  |  |  |  |  |  |  |  |  |  |  |  |  |  |  |  |  |  |  |  |  |  |  |  |  |  |  |  |  |  |  |  |  |
| 2121 | NO | | NO | | NO | | NO | | NO | | NO | | NO | | NO | | NO | | NO | NO | | NO | | MCI | |  |  |  |  |  |  |  |  |  |  |  |  |  |  |  |  |  |  |  |  |  |  |  |  |  |  |  |  |  |  |  |  |  |  |  |  |  |  |  |  |  |  |  |  |  |
| **Physical exams** | | | | | | | | | | | | | | | | | | | | | | | | | | | | | | | | | | | | | | | | | | | | | | | | | | | | | | | | | | | | | | | | | | | | | | |
| **RID** | **AbdomenProblem** | | **ChestProblem** | | **ExtremitiesProblem** | | **GeneralAppearanceProblem** | | **HeadEyesEarsNoseandThroatProblem** | | **HeartProblem** | | **MusculoskeletalProblem** | | **NeckProblem** | | **PeripheralVascularProblem** | | **SkinAndAppendagesProblem** | **DX** | |  |  |  |  |  |  |  |  |  |  |  |  |  |  |  |  |  |  |  |  |  |  |  |  |  |  |  |  |  |  |  |  |  |  |  |  |  |  |  |  |  |  |  |  |  |  |  |  |  |
| 2121 | NO | | NO | | NO | | NO | | NO | | NO | | NO | | NO | | NO | | NO | MCI | |  |  |  |  |  |  |  |  |  |  |  |  |  |  |  |  |  |  |  |  |  |  |  |  |  |  |  |  |  |  |  |  |  |  |  |  |  |  |  |  |  |  |  |  |  |  |  |  |  |
| **Symptoms** | | | | | | | | | | | | | | | | | | | | | | | | | | | | | | | | | | | | | | | | | | | | | | | | | | | | | | | | | | | | | | | | | | | | | | |
| **RID** | **AbdominalDiscomfort** | | **AnkleSwelling** | | **ShortnessOfBreath** | | **ChestPain** | | **Constipation** | | **Coughing** | | **Crying** | | **Diarrhea** | | **Dizziness** | | **DepressedMood** | **Drowsiness** | | **DryMouth** | | **ElevatedMood** | | **LowEnergy** | | **Fall** | | **Headache** | | **Insomnia** | | **MusculoskeletalPain** | | **Nausea** | **Palpitations** | **Rash** | | **Sweating** | | **UrinaryDiscomfort** | | **UrinaryFrequencyProblem** | | **BlurredVision** | | **Vomiting** | | **Wandering** | | **DX** | |  |  |  |  |  |  |  |  |  |  |  |  |  |  |  |  |  |
| 2121 | NO | | NO | | NO | | NO | | YES | | NO | | NO | | NO | | NO | | NO | NO | | NO | | NO | | NO | | NO | | NO | | NO | | YES | | NO | NO | NO | | NO | | NO | | NO | | NO | | NO | | NO | | MCI | |  |  |  |  |  |  |  |  |  |  |  |  |  |  |  |  |  |
| **Vital signs** | | | | | | | | | | | | | | | | | | | | | | | | | | | | | | | | | | | | | | | | | | | | | | | | | | | | | | | | | | | | | | | | | | | | | | |
| **RID** | **BMI** | | **DiastolicBloodPressure** | | **SystolicBloodPressure** | | **Height** | | **SeatedPulseRatePerMinute** | | **RespirationsPerMinute** | | **Temperature** | | **Weight** | | **DX** | |  |  |  |  |  |  |  |  |  |  |  |  |  |  |  |  |  |  |  |  |  |  |  |  |  |  |  |  |  |  |  |  |  |  |  |  |  |  |  |  |  |  |  |  |  |  |  |  |  |  |  |  |
| 2121 | 26.3 | | 84.0 | | 154.0 | | 161.9 | | 67.0 | | 16.0 | | 36.0 | | 69.0 | | MCI | |  |  |  |  |  |  |  |  |  |  |  |  |  |  |  |  |  |  |  |  |  |  |  |  |  |  |  |  |  |  |  |  |  |  |  |  |  |  |  |  |  |  |  |  |  |  |  |  |  |  |  |  |
| **MRI** | | | | | | | | | | | | | | | | | | | | | | | | | | | | | | | | | | | | | | | | | | | | | | | | | | | | | | | | | | | | | | | | | | | | | | |
| **RID** | **Entorhinal** | | **Fusiform** | | **Hippocampus** | | **MidTemp** | | **Ventricles** | | **Volume of Icv** | | **Whole_brain_volume** | | **DX** | |  |  |  |  |  |  |  |  |  |  |  |  |  |  |  |  |  |  |  |  |  |  |  |  |  |  |  |  |  |  |  |  |  |  |  |  |  |  |  |  |  |  |  |  |  |  |  |  |  |  |  |  |  |  |
| 2121 | 3541 | | 22103 | | 6835 | | 22165 | | 22677 | | 1578870 | | 1142447 | | MCI | |  |  |  |  |  |  |  |  |  |  |  |  |  |  |  |  |  |  |  |  |  |  |  |  |  |  |  |  |  |  |  |  |  |  |  |  |  |  |  |  |  |  |  |  |  |  |  |  |  |  |  |  |  |  |

**3. Case Study 3: AD**

| **PET** | | | | |
| --- | --- | --- | --- | --- |
| **RID** | **FDG** | **HCI** | **SROI** | **DX** |
| 753 | 5.2 | 24.4 | 1.1 | AD |

**MRI**

| **RID** | **Entorhinal** | **Fusiform** | **Hippocampus** | **MidTemp** | **Ventricles** | **Volume of Icv** | **Whole_brain_volume** | **DX** |
| --- | --- | --- | --- | --- | --- | --- | --- | --- |
| 753 | 2243 | 15700 | 5789 | 20003 | 95613 | 1772560 | 1012450 | AD |

**Cognitive Scores**

| **RID** | **ADAS 11** | **ADAS 13** | **ADNI_EF** | **ADNI_MEM** | **CDGLOBAL** | **CDRSB** | **FAQTOTAL** | **GDTOTAL** | **HMSCORE** | **MMSCORE** | **MOCA** | **NPISCORE** | **DX** |
| --- | --- | --- | --- | --- | --- | --- | --- | --- | --- | --- | --- | --- | --- |
| 753 | 20.3 | 32.3 | -.6 | -1.3 | 1.0 | 4.5 | 7.0 | 5.0 | 2.0 | 24.0 | 17.2 | 2.0 | AD |

**Genetics**

| **RID** | **ABETA** | **PTAU** | **TAU** | **PolygenicHazardScore** | **CumulativeIncidenceRate** | **DX** |
| --- | --- | --- | --- | --- | --- | --- |
| 753 | 547.6 | 32.1 | 338.4 | 1.7 | .1 | AD |

**Medical history**

| **RID** | **FatherHadDementia** | **FatherHadAlzheimer** | **MotherHadDementia** | **MotherHadAlzheimer** | **HasNeurologicOtherThanAlzheimer** | **HasHeadEyesEarsNoseAndThroatProblem** | **HasCardiovascularProblem** | **HasRespiratoryProblem** | **HasHepaticProblem** | **HasDermatologicConnectiveTissueProblem** | **HasMusculoskeletalProblem** | **HasEndocrineMetabolicProblem** | **HasGastrointestinalProblem** | **HasHematopoieticLymphaticProblem** | **HasRenalGenitourinaryProblem** | **HasAllergiesOrDrugSensitivities** | **IsAlcoholAbuse** | **IsDrugAbuse** | **IsSmoker** | **HasMalignancy** | **DidMajorSurgicalProcedures** | **HasPsychiatric** | **AGE** | **APOE4** | **Marry_Status** | **Gender** | **NumberOfYearsEducation** | **DX** |
| --- | --- | --- | --- | --- | --- | --- | --- | --- | --- | --- | --- | --- | --- | --- | --- | --- | --- | --- | --- | --- | --- | --- | --- | --- | --- | --- | --- | --- |
| 753 | NO | NO | YES | YES | YES | YES | NO | NO | NO | YES | YES | YES | NO | NO | YES | NO | NO | NO | NO | NO | NO | YES | 65.5 | 2.0 | Married | Male | 16.0 | AD |

**Neuropsychological Battery**

| **RID** | **A30MinuteDelayTotal** | **RecognitionScore** | **Trial1Total** | **Trial2Total** | **Trial3Total** | **Trial4Total** | **Trial5Total** | **Trial6Total** | **ListBTotal** | **CategoryFluencyAnimalsTotalCorrect** | **CategoryFluencyAnimalsIntrusions** | **CategoryFluencyAnimalsPerseverations** | **Clock_ApproximatelyCircularFace** | **Clock_PresenceOfTheTwoHands** | **Clock_CorrectnessOfNumbers** | **Clock_TotalScore** | **Clock_SymmetryOfNumberPlacement** | **Clock_PresenceOfTheTwoHandsSetToTenAfterEleven** | **Copy_ApproximatelyCircularFace** | **Copy_PresenceOfTheTwoHands** | **Copy_CorrectnessOfNumbers** | **Copy_TotalScore** | **Copy_SymmetryOfNumberPlacement** | **Copy_PresenceOfTheTwoHandsSetToTenAfterEleven** | **DigitTotalScore** | **RAVLT_forgetting** | **RAVLT_immediate** | **RAVLT_learning** | **RAVLT_perc_forgetting** | **ErrorsOfCommission** | **ErrorsOfOmission** | **TRAASCOR_PartATimeToComplete** | **TRABERRCOM_ErrorsOfCommission** | **TRABERROM_ErrorsOfOmission** | **TRABSCOR_PartBTimeToComplete** | **DX** |
| --- | --- | --- | --- | --- | --- | --- | --- | --- | --- | --- | --- | --- | --- | --- | --- | --- | --- | --- | --- | --- | --- | --- | --- | --- | --- | --- | --- | --- | --- | --- | --- | --- | --- | --- | --- | --- |
| 753 | .0 | 6.0 | 4.0 | 4.0 | 3.0 | 5.0 | 2.0 | 1.0 | 5.0 | 21.0 | .0 | 2.0 | YES | YES | YES | 4.0 | YES | NO | YES | NO | YES | 4.0 | YES | YES | 15.0 | 2.0 | 18.0 | -2.0 | 100.0 | .0 | .0 | 37.0 | 4.0 | 5.0 | 300.0 | AD |

**Symptoms**

| **RID** | **AbdominalDiscomfort** | **AnkleSwelling** | **ShortnessOfBreath** | **ChestPain** | **Constipation** | **Coughing** | **Crying** | **Diarrhea** | **Dizziness** | **DepressedMood** | **Drowsiness** | **DryMouth** | **ElevatedMood** | **LowEnergy** | **Fall** | **Headache** | **Insomnia** | **MusculoskeletalPain** | **Nausea** | **Palpitations** | **Rash** | **Sweating** | **UrinaryDiscomfort** | **UrinaryFrequencyProblem** | **BlurredVision** | **Vomiting** | **Wandering** | **DX** |
| --- | --- | --- | --- | --- | --- | --- | --- | --- | --- | --- | --- | --- | --- | --- | --- | --- | --- | --- | --- | --- | --- | --- | --- | --- | --- | --- | --- | --- |
| 753 | NO | NO | NO | NO | NO | NO | YES | NO | YES | YES | NO | NO | NO | NO | NO | NO | NO | NO | NO | NO | NO | NO | NO | NO | NO | NO | NO | AD |

**Vital signs**

| **RID** | **BMI** | **DiastolicBloodPressure** | **SystolicBloodPressure** | **Height** | **SeatedPulseRatePerMinute** | **RespirationsPerMinute** | **Temperature** | **Weight** | **DX** |
| --- | --- | --- | --- | --- | --- | --- | --- | --- | --- |
| 753 | 22.8 | 95.0 | 174.0 | 172.7 | 50.0 | 16.0 | 35.6 | 68.0 | AD |

**4. Case Study 4: sMCI**

**Cognitive Scores**

| **RID** | **ADAS 11** | **ADAS 13** | **ADNI_EF** | **ADNI_MEM** | **CDGLOBAL** | **CDRSB** | **FAQTOTAL** | **GDTOTAL** | **HMSCORE** | **MMSCORE** | **MOCA** | **NPISCORE** | **DX** |
| --- | --- | --- | --- | --- | --- | --- | --- | --- | --- | --- | --- | --- | --- |
| 4849 | 4.0 | 9.0 | 1.3 | .9 | .5 | 1.5 | .0 | 4.0 | 1.0 | 29.0 | 28.0 | .0 | sMCI |

**Genetics**

| **RID** | **ABETA** | **PTAU** | **TAU** | **PolygenicHazardScore** | **CumulativeIncidenceRate** | **DX** |
| --- | --- | --- | --- | --- | --- | --- |
| 4849 | 691.3 | 26.9 | 277.3 | 1.9 | .1 | sMCI |

**Medical history**

| **RID** | **FatherHadDementia** | **FatherHadAlzheimer** | **MotherHadDementia** | **MotherHadAlzheimer** | **HasNeurologicOtherThanAlzheimer** | **HasHeadEyesEarsNoseAndThroatProblem** | **HasCardiovascularProblem** | **HasRespiratoryProblem** | **HasHepaticProblem** | **HasDermatologicConnectiveTissueProblem** | **HasMusculoskeletalProblem** | **HasEndocrineMetabolicProblem** | **HasGastrointestinalProblem** | **HasHematopoieticLymphaticProblem** | **HasRenalGenitourinaryProblem** | **HasAllergiesOrDrugSensitivities** | **IsAlcoholAbuse** | **IsDrugAbuse** | **IsSmoker** | **HasMalignancy** | **DidMajorSurgicalProcedures** | **HasPsychiatric** | **AGE** | **APOE4** | **Marry_Status** | **Gender** | **NumberOfYearsEducation** | **DX** |
| --- | --- | --- | --- | --- | --- | --- | --- | --- | --- | --- | --- | --- | --- | --- | --- | --- | --- | --- | --- | --- | --- | --- | --- | --- | --- | --- | --- | --- |
| 4849 | NO | YES | NO | YES | NO | NO | YES | NO | NO | NO | YES | NO | NO | NO | YES | NO | NO | NO | NO | NO | NO | YES | 65.9 | 2.0 | Divorced | Female | 18.0 | sMCI |

**Lab tests**

| **RID** | **BAT126_VitaminB12** | **RedBloodCellCount** | **WhiteBloodCellCount** | **GlucoseResults** | **HMT2_Hematocrit** | **HMT3_RBC** | **HMT4_MCV** | **HMT7_WBC** | **HMT8_Neutrophils** | **HMT9_Lymphocytes** | **HMT10_Monocytes** | **HMT11_Eosinophils** | **HMT12_Basophils** | **HMT13_Platelets** | **HMT15_NeutrophilsPercentage** | **HMT16_LymphocytesPercentage** | **HMT17_MonocytesPercentage** | **HMT18_EosinophilsPercentage** | **HMT19_BasophilsPercentage** | **HMT40_Hemoglobin** | **HMT100_MCH** | **HMT102_MCHC** | **ProteinResults** | **RCT1_TotalBilirubin** | **RCT3_GGT** | **RCT4_ALT_SGPT** | **RCT5_AST_SGOT** | **RCT6_UreaNitrogen** | **RCT8_SerumUricAcid** | **RCT9_Phosphorus** | **RCT11_SerumGlucose** | **RCT12_TotalProtein** | **RCT13_Albumin** | **RCT14_CreatineKinase** | **RCT19_Triglycerides_GPO** | **RCT20_Cholesterol** | **RCT29_DirectBilirubin** | **RCT183_Calcium_EDTA** | **RCT392_Creatinine** | **RCT1407_AlkalinePhosphatase** | **RCT1408_LDH** | **DX** |
| --- | --- | --- | --- | --- | --- | --- | --- | --- | --- | --- | --- | --- | --- | --- | --- | --- | --- | --- | --- | --- | --- | --- | --- | --- | --- | --- | --- | --- | --- | --- | --- | --- | --- | --- | --- | --- | --- | --- | --- | --- | --- | --- |
| 4849 | 730.0 | 75.0 | .0 | 52.0 | 38.0 | 4.1 | 92.0 | 4.9 | 2.9 | 1.5 | .3 | .3 | .0 | 221.0 | 58.4 | 29.7 | 6.1 | 5.1 | .7 | 13.6 | 33.0 | 36.0 | 24.0 | .5 | 18.0 | 22.0 | 32.0 | 17.0 | 8.8 | 4.1 | 98.0 | 7.2 | 4.3 | 151.0 | 77.0 | 180.0 | .1 | 10.4 | .9 | 49.0 | 177.0 | sMCI |

**Neuropshychological Battery**

| **RID** | **A30MinuteDelayTotal** | **RecognitionScore** | **Trial1Total** | **Trial2Total** | **Trial3Total** | **Trial4Total** | **Trial5Total** | **Trial6Total** | **ListBTotal** | **CategoryFluencyAnimalsTotalCorrect** | **CategoryFluencyAnimalsIntrusions** | **CategoryFluencyAnimalsPerseverations** | **Clock_ApproximatelyCircularFace** | **Clock_PresenceOfTheTwoHands** | **Clock_CorrectnessOfNumbers** | **Clock_TotalScore** | **Clock_SymmetryOfNumberPlacement** | **Clock_PresenceOfTheTwoHandsSetToTenAfterEleven** | **Copy_ApproximatelyCircularFace** | **Copy_PresenceOfTheTwoHands** | **Copy_CorrectnessOfNumbers** | **Copy_TotalScore** | **Copy_SymmetryOfNumberPlacement** | **Copy_PresenceOfTheTwoHandsSetToTenAfterEleven** | **DigitTotalScore** | **RAVLT_forgetting** | **RAVLT_immediate** | **RAVLT_learning** | **RAVLT_perc_forgetting** | **ErrorsOfCommission** | **ErrorsOfOmission** | **TRAASCOR_PartATimeToComplete** | **TRABERRCOM_ErrorsOfCommission** | **TRABERROM_ErrorsOfOmission** | **TRABSCOR_PartBTimeToComplete** | **DX** |
| --- | --- | --- | --- | --- | --- | --- | --- | --- | --- | --- | --- | --- | --- | --- | --- | --- | --- | --- | --- | --- | --- | --- | --- | --- | --- | --- | --- | --- | --- | --- | --- | --- | --- | --- | --- | --- |
| 4849 | 5.0 | 14.0 | 6.0 | 6.0 | 13.0 | 13.0 | 13.0 | 10.0 | 4.0 | 27.0 | .0 | 1.0 | YES | YES | YES | 5.0 | YES | YES | YES | YES | YES | 5.0 | YES | YES | 34.0 | 8.0 | 51.0 | 7.0 | 61.5 | .0 | .0 | 29.0 | .0 | .0 | 61.0 | sMCI |

**Neurological Exams**

| **RID** | **HasSignificantAuditoryImpairment** | **HasLevelofConsciousness** | **HasCerebellarFingerToNose** | **HasGait** | **HasCerebellarHeelToShin** | **HasMotorStrengthProblem** | **HasCranialNervesProblem** | **HasPlantarReflexesProblem** | **HasSensoryProblem** | **HasDeepTendonReflexesProblem** | **HasTremorProblem** | **HasSignificantVisualImpairment** | **DX** |
| --- | --- | --- | --- | --- | --- | --- | --- | --- | --- | --- | --- | --- | --- |
| 4849 | NO | NO | NO | NO | NO | NO | NO | NO | NO | NO | NO | NO | sMCI |

**Physical exams**

| **RID** | **AbdomenProblem** | **ChestProblem** | **ExtremitiesProblem** | **GeneralAppearanceProblem** | **HeadEyesEarsNoseandThroatProblem** | **HeartProblem** | **MusculoskeletalProblem** | **NeckProblem** | **PeripheralVascularProblem** | **SkinAndAppendagesProblem** | **DX** |
| --- | --- | --- | --- | --- | --- | --- | --- | --- | --- | --- | --- |
| 4849 | NO | NO | NO | NO | NO | NO | NO | NO | NO | NO | sMCI |

**Vital Signs**

| **RID** | **BMI** | **DiastolicBloodPressure** | **SystolicBloodPressure** | **Height** | **SeatedPulseRatePerMinute** | **RespirationsPerMinute** | **Temperature** | **Weight** | **DX** |
| --- | --- | --- | --- | --- | --- | --- | --- | --- | --- |
| 4849 | 23.6 | 64.0 | 122.0 | 154.9 | 72.0 | 16.0 | 35.5 | 56.7 | sMCI |

**MRI**

| **RID** | **Entorhinal** | **Fusiform** | **Hippocampus** | **MidTemp** | **Ventricles** | **Volume of Icv** | **Whole_brain_volume** | **DX** |
| --- | --- | --- | --- | --- | --- | --- | --- | --- |
| 4849 | 2918 | 17360 | 6707 | 16434 | 16521 | 1299290 | 947601 | sMCI |

**Symptoms**

| **RID** | **AbdominalDiscomfort** | **AnkleSwelling** | **ShortnessOfBreath** | **ChestPain** | **Constipation** | **Coughing** | **Crying** | **Diarrhea** | **Dizziness** | **DepressedMood** | **Drowsiness** | **DryMouth** | **ElevatedMood** | **LowEnergy** | **Fall** | **Headache** | **Insomnia** | **MusculoskeletalPain** | **Nausea** | **Palpitations** | **Rash** | **Sweating** | **UrinaryDiscomfort** | **UrinaryFrequencyProblem** | **BlurredVision** | **Vomiting** | **Wandering** | **DX** |
| --- | --- | --- | --- | --- | --- | --- | --- | --- | --- | --- | --- | --- | --- | --- | --- | --- | --- | --- | --- | --- | --- | --- | --- | --- | --- | --- | --- | --- |
| 4849.0 | NO | NO | NO | NO | NO | NO | NO | NO | NO | NO | NO | NO | NO | NO | NO | NO | NO | NO | NO | NO | NO | NO | NO | NO | NO | NO | NO | sMCI |

**5. Case Study 5: pMCI**

**PET**

| **RID** | **FDG** | **HCI** | **SROI** | **DX** |
| --- | --- | --- | --- | --- |
| 4796.0 | 5.5 | 15.2 | 1.2 | pMCI |

**MRI**

| **RID** | **Entorhinal** | **Fusiform** | **Hippocampus** | **MidTemp** | **Ventricles** | **Volume of Icv** | **Whole_brain_volume** | **DX** |
| --- | --- | --- | --- | --- | --- | --- | --- | --- |
| 4796 | 2860 | 13975 | 6198 | 15172 | 33754 | 1431710 | 963477 | pMCI |

**Cognitive Scores**

| **RID** | **ADAS 11** | **ADAS 13** | **ADNI_EF** | **ADNI_MEM** | **CDGLOBAL** | **CDRSB** | **FAQTOTAL** | **GDTOTAL** | **HMSCORE** | **MMSCORE** | **MOCA** | **NPISCORE** | **DX** |
| --- | --- | --- | --- | --- | --- | --- | --- | --- | --- | --- | --- | --- | --- |
| 4796.0 | 18.0 | 31.0 | -.9 | -1.1 | .5 | 2.0 | 6.0 | 3.0 | .0 | 25.0 | 17.0 | 4.0 | pMCI |

**Genetics**

| **RID** | **ABETA** | **PTAU** | **TAU** | **PolygenicHazardScore** | **CumulativeIncidenceRate** | **DX** |
| --- | --- | --- | --- | --- | --- | --- |
| 4796.0 | 755.2 | 74.5 | 670.1 | -.3 | .0 | pMCI |

**Medical History**

| **RID** | **FatherHadDementia** | **FatherHadAlzheimer** | **MotherHadDementia** | **MotherHadAlzheimer** | **HasNeurologicOtherThanAlzheimer** | **HasHeadEyesEarsNoseAndThroatProblem** | **HasCardiovascularProblem** | **HasRespiratoryProblem** | **HasHepaticProblem** | **HasDermatologicConnectiveTissueProblem** | **HasMusculoskeletalProblem** | **HasEndocrineMetabolicProblem** | **HasGastrointestinalProblem** | **HasHematopoieticLymphaticProblem** | **HasRenalGenitourinaryProblem** | **HasAllergiesOrDrugSensitivities** | **IsAlcoholAbuse** | **IsDrugAbuse** | **IsSmoker** | **HasMalignancy** | **DidMajorSurgicalProcedures** | **HasPsychiatric** | **AGE** | **APOE4** | **Marry_Status** | **Gender** | **NumberOfYearsEducation** | **DX** |
| --- | --- | --- | --- | --- | --- | --- | --- | --- | --- | --- | --- | --- | --- | --- | --- | --- | --- | --- | --- | --- | --- | --- | --- | --- | --- | --- | --- | --- |
| 4796.0 | NO | YES | NO | YES | NO | YES | NO | NO | NO | NO | YES | NO | YES | NO | NO | NO | NO | NO | NO | NO | YES | YES | 72.6 | .0 | Married | Female | 16.0 | pMCI |

**Lab Tests**

| **RID** | **BAT126_VitaminB12** | **RedBloodCellCount** | **WhiteBloodCellCount** | **GlucoseResults** | **HMT2_Hematocrit** | **HMT3_RBC** | **HMT4_MCV** | **HMT7_WBC** | **HMT8_Neutrophils** | **HMT9_Lymphocytes** | **HMT10_Monocytes** | **HMT11_Eosinophils** | **HMT12_Basophils** | **HMT13_Platelets** | **HMT15_NeutrophilsPercentage** | **HMT16_LymphocytesPercentage** | **HMT17_MonocytesPercentage** | **HMT18_EosinophilsPercentage** | **HMT19_BasophilsPercentage** | **HMT40_Hemoglobin** | **HMT100_MCH** | **HMT102_MCHC** | **ProteinResults** | **RCT1_TotalBilirubin** | **RCT3_GGT** | **RCT4_ALT_SGPT** | **RCT5_AST_SGOT** | **RCT6_UreaNitrogen** | **RCT8_SerumUricAcid** | **RCT9_Phosphorus** | **RCT11_SerumGlucose** | **RCT12_TotalProtein** | **RCT13_Albumin** | **RCT14_CreatineKinase** | **RCT19_Triglycerides_GPO** | **RCT20_Cholesterol** | **RCT29_DirectBilirubin** | **RCT183_Calcium_EDTA** | **RCT392_Creatinine** | **RCT1407_AlkalinePhosphatase** | **RCT1408_LDH** | **DX** |
| --- | --- | --- | --- | --- | --- | --- | --- | --- | --- | --- | --- | --- | --- | --- | --- | --- | --- | --- | --- | --- | --- | --- | --- | --- | --- | --- | --- | --- | --- | --- | --- | --- | --- | --- | --- | --- | --- | --- | --- | --- | --- | --- |
| 4796.0 | 553.0 | .0 | .0 | 65.0 | 43.0 | 5.0 | 87.0 | 7.8 | 5.8 | 1.4 | .4 | .2 | .1 | 289.0 | 74.1 | 17.7 | 5.0 | 2.5 | .6 | 13.9 | 28.0 | 32.0 | 39.0 | .8 | 17.0 | 12.0 | 18.0 | 18.0 | 4.3 | 4.2 | 99.0 | 7.2 | 4.0 | 42.0 | 112.0 | 203.0 | .2 | 9.2 | .8 | 124.0 | 155.0 | pMCI |

**Neuropsychological Battery**

| **RID** | **A30MinuteDelayTotal** | **RecognitionScore** | **Trial1Total** | **Trial2Total** | **Trial3Total** | **Trial4Total** | **Trial5Total** | **Trial6Total** | **ListBTotal** | **CategoryFluencyAnimalsTotalCorrect** | **CategoryFluencyAnimalsIntrusions** | **CategoryFluencyAnimalsPerseverations** | **Clock_ApproximatelyCircularFace** | **Clock_PresenceOfTheTwoHands** | **Clock_CorrectnessOfNumbers** | **Clock_TotalScore** | **Clock_SymmetryOfNumberPlacement** | **Clock_PresenceOfTheTwoHandsSetToTenAfterEleven** | **Copy_ApproximatelyCircularFace** | **Copy_PresenceOfTheTwoHands** | **Copy_CorrectnessOfNumbers** | **Copy_TotalScore** | **Copy_SymmetryOfNumberPlacement** | **Copy_PresenceOfTheTwoHandsSetToTenAfterEleven** | **DigitTotalScore** | **RAVLT_forgetting** | **RAVLT_immediate** | **RAVLT_learning** | **RAVLT_perc_forgetting** | **ErrorsOfCommission** | **ErrorsOfOmission** | **TRAASCOR_PartATimeToComplete** | **TRABERRCOM_ErrorsOfCommission** | **TRABERROM_ErrorsOfOmission** | **TRABSCOR_PartBTimeToComplete** | **DX** |
| --- | --- | --- | --- | --- | --- | --- | --- | --- | --- | --- | --- | --- | --- | --- | --- | --- | --- | --- | --- | --- | --- | --- | --- | --- | --- | --- | --- | --- | --- | --- | --- | --- | --- | --- | --- | --- |
| 4796.0 | .0 | 15.0 | 3.0 | 3.0 | 3.0 | 6.0 | 5.0 | .0 | 2.0 | 19.0 | .0 | 1.0 | YES | YES | YES | 4.0 | YES | NO | YES | YES | YES | 5.0 | YES | YES | 34.0 | 5.0 | 20.0 | 2.0 | 100.0 | .0 | .0 | 50.0 | 3.0 | .0 | 285.0 | pMCI |

**Symptoms**

| **RID** | **AbdominalDiscomfort** | **AnkleSwelling** | **ShortnessOfBreath** | **ChestPain** | **Constipation** | **Coughing** | **Crying** | **Diarrhea** | **Dizziness** | **DepressedMood** | **Drowsiness** | **DryMouth** | **ElevatedMood** | **LowEnergy** | **Fall** | **Headache** | **Insomnia** | **MusculoskeletalPain** | **Nausea** | **Palpitations** | **Rash** | **Sweating** | **UrinaryDiscomfort** | **UrinaryFrequencyProblem** | **BlurredVision** | **Vomiting** | **Wandering** | **DX** |
| --- | --- | --- | --- | --- | --- | --- | --- | --- | --- | --- | --- | --- | --- | --- | --- | --- | --- | --- | --- | --- | --- | --- | --- | --- | --- | --- | --- | --- |
| 4796.0 | NO | NO | NO | NO | NO | NO | NO | NO | NO | NO | NO | YES | NO | NO | NO | NO | NO | YES | NO | NO | NO | NO | NO | NO | NO | NO | NO | pMCI |

**PART 4: ETHICS COMMITTEES/INSTITUTIONAL REVIEW BOARDS**

The Ethics committees/institutional review boards that approved the ADNI study are: Albany Medical Center Committee on Research Involving Human Subjects Institutional Review Board, Boston University Medical Campus and Boston Medical Center Institutional Review Board, Butler Hospital Institutional Review Board, Cleveland Clinic Institutional Review Board, Columbia University Medical Center Institutional Review Board, Duke University Health System Institutional Review Board, Emory Institutional Review Board, Georgetown University Institutional Review Board, Health Sciences Institutional Review Board, Houston Methodist Institutional Review Board, Howard University Office of Regulatory Research Compliance, Icahn School of Medicine at Mount Sinai Program for the Protection of Human Subjects, Indiana University Institutional Review Board, Institutional Review Board of Baylor College of Medicine, Jewish General Hospital Research Ethics Board, Johns Hopkins Medicine Institutional Review Board, Lifespan - Rhode Island Hospital Institutional Review Board, Mayo Clinic Institutional Review Board, Mount Sinai Medical Center Institutional Review Board, Nathan Kline Institute for Psychiatric Research & Rockland Psychiatric Center Institutional Review Board, New York University Langone Medical Center School of Medicine Institutional Review Board, Northwestern University Institutional Review Board, Oregon Health and Science University Institutional Review Board, Partners Human Research Committee Research Ethics, Board Sunnybrook Health Sciences Centre, Roper St. Francis Healthcare Institutional Review Board, Rush University Medical Center Institutional Review Board, St. Joseph's Phoenix Institutional Review Board, Stanford Institutional Review Board, The Ohio State University Institutional Review Board, University Hospitals Cleveland Medical Center Institutional Review Board, University of Alabama Office of the IRB, University of British Columbia Research Ethics Board, University of California Davis Institutional Review Board Administration, University of California Los Angeles Office of the Human Research Protection Program, University of California San Diego Human Research Protections Program, University of California San Francisco Human Research Protection Program, University of Iowa Institutional Review Board, University of Kansas Medical Center Human Subjects Committee, University of Kentucky Medical Institutional Review Board, University of Michigan Medical School Institutional Review Board, University of Pennsylvania Institutional Review Board, University of Pittsburgh Institutional Review Board, University of Rochester Research Subjects Review Board, University of South Florida Institutional Review Board, University of Southern, California Institutional Review Board, UT Southwestern Institution Review Board, VA Long Beach Healthcare System Institutional Review Board, Vanderbilt University Medical Center Institutional Review Board, Wake Forest School of Medicine Institutional Review Board, Washington University School of Medicine Institutional Review Board, Western Institutional Review Board, Western University Health Sciences Research Ethics Board, and Yale University Institutional Review Board.
